# AVATAR versus cognitive-behavioral therapy for medication-resistant auditory hallucination: a systematic review and network meta-analysis

**DOI:** 10.1017/S0033291726104127

**Published:** 2026-04-13

**Authors:** Tien-Wei Hsu, Chih-Sung Liang, Te-Chang Changchien, Ping-Tao Tseng, Andre F. Carvalho, Brendon Stubbs, Trevor Thompson, Kerem Böge, Chih-Wei Hsu, Fu-Chi Yang, Yu-Kang Tu, Yu-Hsuan Lin

**Affiliations:** 1 Department of Psychiatry, E-DA Dachang Hospital, I-Shou University, Kaohsiung, Taiwan; 2 Department of Psychiatry, E-DA Hospital, I-Shou University, Kaohsiung, Taiwan; 3 Graduate Institute of Clinical Medicine, College of Medicine, Kaohsiung Medical University, Kaohsiung, Taiwan; 4 School of Medicine, College of Medicine, I-Shou University, Kaohsiung, Taiwan; 5 Department of Psychiatry, Tri-Service General Hospital, National Defense Medical Center, Taipei, Taiwan; 6 Department of Psychiatry, Beitou Branch, Tri-Service General Hospital, Taipei, Taiwan; 7 Institute of Biomedical Sciences, National Sun Yat-sen University, Kaohsiung, Taiwan; 8 Department of Psychology, College of Medical and Health Science, Asia University, Taichung, Taiwan; 9 Prospect Clinic for Otorhinolaryngology & Neurology, Kaohsiung, Taiwan; 10 Institute of Precision Medicine, National Sun Yat-sen University, Kaohsiung City, Taiwan; 11 IMPACT (Innovation in Mental and Physical Health and Clinical Treatment) Strategic Research Centre, School of Medicine, Barwon Health, Deakin University, Geelong, VIC, Australia; 12 Institute of Psychiatry, Psychology and Neuroscience, King’s College London; 13 Centre for Chronic Illness and Ageing, University of Greenwich, London, UK; 14 Charité Universitätsmedizin Berlin, Department for Psychiatry and Psychotherapy, Campus Charité Mitte (CCM), Berlin, Germany; 15 Department of Psychiatry, Kaohsiung Chang Gung Memorial Hospital and Chang Gung University College of Medicine, Kaohsiung, Taiwan; 16 Department of Neurology, Tri-Service General Hospital, National Defense Medical Centre, Taipei, Taiwan; 17 Institute of Epidemiology & Preventive Medicine, College of Public Health, National Taiwan University, Taipei, Taiwan; 18 Department of Dentistry, National Taiwan University Hospital, Taipei, Taiwan; 19 Institute of Population Health Sciences, National Health Research Institutes, Miaoli County, Taiwan; 20 Department of Psychiatry, College of Medicine, National Taiwan University, Taipei, Taiwan; 21 Department of Biomedical Sciences and Engineering, National Central University, Taoyuan City, Taiwan

**Keywords:** auditory hallucinations, AVATAR, CBT, psychosis, schizophrenia spectrum disorder, treatment resistance, network meta-analysis

## Abstract

Auditory hallucinations (AH) frequently persist in schizophrenia spectrum disorder despite antipsychotic treatment. Cognitive behavioral therapy (CBT) remains an established psychological intervention, whereas AVATAR (Audio Visual Assisted Therapy Aid for Refractory auditory hallucinations) therapy has recently been introduced as a novel approach integrating interactive digital avatars. This meta-analysis compared the efficacy of AVATAR therapy with CBT for medication-resistant AH. A systematic search of five major databases up to June 1, 2025 identified randomized controlled trials (RCTs) that evaluated either therapy. The primary outcome was AH severity, and secondary outcomes included psychotic symptoms, mood measures, and all-cause discontinuation. Twenty-six RCTs (n = 2273; 65.0% male; mean age 39.3 [SD 4.1] years) met inclusion criteria. Compared with CBT, AVATAR therapy showed no significantly greater reduction in AH severity (standardized mean difference [SMD] = −0.23, 95% confidence interval [CI] = −0.55 to 0.10). However, it demonstrated superior sustained improvement three months post-treatment (SMD = −0.37, 95% CI = −0.69 to −0.05) and greater reduction in overall psychotic symptoms (SMD = −0.41, 95% CI = −0.75 to −0.06). No significant differences were observed in positive, negative, depressive, anxiety, or quality-of-life outcomes, and discontinuation rates were comparable. Interpretation should be cautious given evidence of small-study effects (Egger’s tests *p* < 0.01 for AH severity) and predominantly moderate-to-high risk of bias across included trials. AVATAR therapy therefore shows lasting efficacy, comparable or slightly superior to CBT, and may serve as an alternative for patients with medication-resistant AH.

## Introduction

Schizophrenia spectrum disorders (SSD) affect approximately 24 million people globally and are associated with profound morbidity and disability (Solmi et al., 2023). Auditory hallucinations (AH), such as hearing voices, are among the most prevalent symptoms of SSD, reported by as many as 75% of patients (Waters & Fernyhough, 2017). Despite treatment with antipsychotic medications, a significant proportion of individuals continue to experience persistent AH. Research indicates that about 30% of patients with psychotic symptoms experienced medication-resistant AH (Sommer et al., 2012). However, patients with persistent hallucinations are associated with poor prognosis, depressive and anxiety symptoms, increase suicide risk, higher hospitalization rate, and higher healthcare costs, highlighting the need for improved therapeutic strategies therapeutic strategies (Birchwood et al., 2004; Fivel, Mondino, Brunelin, & Haesebaert, 2023; Kjelby et al., 2015).

**Figure 1.**
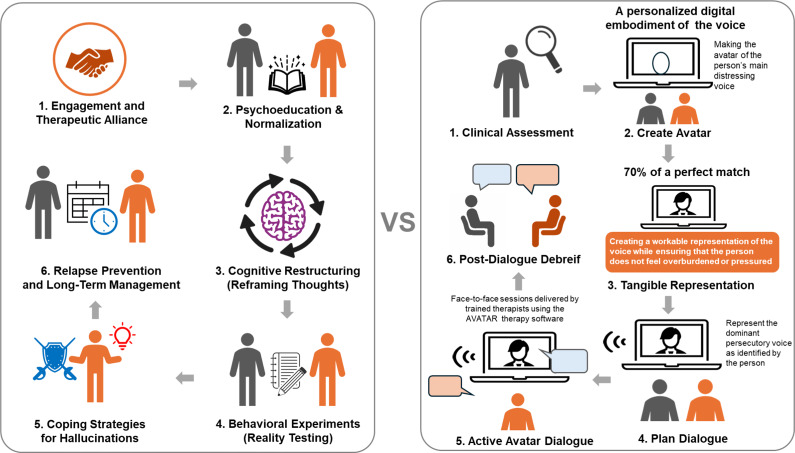
Illustration of CBT and AVATAR for treatment-resistant auditory hallucinations.

With advancements in technology, there has been an increase in the use of psychotherapeutic treatments that incorporate virtual reality and avatars (computer-generated human-like figures designed to interact with users). In 2013, Leff et al. developed AVATAR therapy (Audio Visual Assisted Therapy Aid for Refractory Auditory Hallucinations; AVATAR), a virtual reality-based modification of CBT designed for patients with medication-resistant AH with SSD. This novel approach enables patients to engage in face-to-face dialogue with a digital representation that matches their persecutory voice in pitch and tone. During sessions, the therapist alternates between speaking as the avatar and as themselves, facilitating a therapeutic process where patients gradually gain increased power and control over their voice experiences. Through this innovative integration of digital technology with psychological intervention, patients can externalize the hallucinated voice and engage in structured role plays, ultimately reducing distress and emotional reactivity (Leff et al., 2013). Recently, Dellazizzo et al. conducted a randomized controlled trial comparing CBT and AVATAR therapy for medication-resistant AH in schizophrenia (Dellazizzo, Potvin, Phraxayavong, & Dumais, 2021). Despite the lack of statistically significant differences between treatments, AVATAR therapy produced a large effect size for AH, in contrast to the medium effect size observed with CBT (Dellazizzo et al., 2021) (Figure 1).

CBT in schizophrenia has been shown to reduce positive symptoms, depression, and overall symptom severity, with modest effects on negative symptoms. It improves coping, adherence, and symptom management, can be safely delivered by trained professionals in various settings, and shows durable benefits when combined with antipsychotic medication and other psychosocial interventions (Turkington, Dudley, Warman, & Beck, 2004). Many clinical guidelines recommend cognitive behavioral therapy (CBT) for medication-resistant schizophrenia, especially for positive symptoms. The Royal Australian and New Zealand College of Psychiatrists (RANZCP) guidelines (Galletly et al., 2016) suggest that CBT should be provided to individuals experiencing persistent psychotic symptoms, particularly when these symptoms do not respond to antipsychotic treatment. The National Institute for Health and Care Excellence (NICE) guidelines state that CBT is effective in reducing the severity of positive symptoms (Kuipers, Yesufu-Udechuku, Taylor, & Kendall, 2014). The American Psychiatric Association guidelines recommend that CBT should be given to patients with schizophrenia, as it may help decrease the frequency, severity, and distress of positive symptoms (Keepers et al., 2020). In addition, a recent meta-analysis (Salahuddin et al., 2024) found that CBT had a significantly greater effect on the positive symptoms of schizophrenia compared to treatment-as-usual (TAU) (effect size = −0.31, 95% confidence intervals [CI] = −0.43 to −0.19).

Despite the emerging popularity of AVATAR therapy and the established use of conventional CBT, direct comparative evidence between these two approaches remains limited. Therefore, we conducted a network meta-analysis comparing the efficacy of CBT and AVATAR therapy for treating medication-resistant AH through direct and indirect comparisons. Additionally, we also assessed its long-term efficacy after discontinuation to determine the durability of treatment benefits.

## Materials and methods

Prior to data analysis, we registered our protocol on PROSPERO (CRD420251056279). The study adhered to the Preferred Reporting Items for Systematic Reviews and Meta analyses (PRISMA) (Page et al., 2021), which can be found in Supplementary Appendix 1. To ensure transparency, we confirm that none of the authors of this network meta-analysis were investigators in the original RCTs included.

### Eligibility criteria

We included randomized controlled trials (RCTs) of adult patients (>18 years old) with SSD who had persistent medication-resistant auditory hallucinations. The included studies were required to compare AVATAR therapy or CBT with control interventions. The control group could receive either passive (e.g., treatment as usual or waitlist) or active (e.g. CBT or other active interventions, such as supportive psychotherapy or psychoeducation) treatments. The exclusion criteria were: (i) non-randomized studies, (ii) studies without specific measurements for auditory hallucination, (iii) studies with patients who had not received adequate antipsychotic treatment, and (iv) studies not published in peer-reviewed journals. If the study includes a small proportion of mood disorder with auditory hallucinations, it is acceptable; however, participants with SSD must account for more than 90% of the sample.

### Data sources and search

Two reviewers independently searched MEDLINE, the Cochrane Central Register of Controlled Trials (CENTRAL), EMBASE, ClinicalTrial.gov, and PubMed without language restrictions to May 31, 2025. We also reviewed the reference lists of the included studies and related systematic reviews.

### Study selection

Two reviewers independently screened titles, abstracts, and full-text articles. Disagreements were resolved through discussion and, if necessary, by consulting the corresponding authors. Supplementary Appendix 2 demonstrates the complete search strategies and Supplementary appendix 3 shows the reasons for exclusion.

### Data extraction and outcome definition

Two reviewers independently extracted the data and discrepancies were resolved by consensus and, when necessary, by consulting the corresponding authors. WebPlot Digitizer (https://apps.automeris.io/wpd/) was used to extract numerical data from the figures. The following data were extracted from each study: authorship, year of publication, country of origin, and study design. For participant characteristics, we collected information on diagnosis type, sample size, mean age with standard deviation (SD), and sex distribution (percentage of females). Intervention details included the number and duration of AVATAR therapy sessions. For comparison groups, we documented the type of control interventions, such as treatment as usual, CBT, or waitlist control.

The primary outcome was the severity of AH, as measured by the score of Psychotic Symptom Rating Scale-Auditory Hallucination or the validated measurement tools. The secondary outcomes were the overall psychotic symptoms, positive symptoms, negative symptoms, quality of life, depression, anxiety, and all-cause discontinuation. If the study provided the follow-up data after the interventions discontinuation, we also extracted the follow-up data.

### Quality assessment

The risk of bias (RoB) was assessed using the Cochrane risk-of-bias tool 2 (RoB-2) for randomized trials. This tool evaluates five domains of potential bias: bias arising from the randomization process, bias due to deviations from intended interventions, bias due to missing outcome data, bias in outcome measurement, and bias in the selection of reported results. Two authors independently conducted this assessment, and discrepancies were resolved by consulting the corresponding authors.

### Data synthesis

We conducted a random-effects network meta-analysis (NMA) within a frequentist framework using the package netmeta R, version 3.2–0. Standardized mean differences (SMDs) for continuous outcomes and risk ratios (RRs) for binary outcomes were calculated and presented with corresponding 95% confidence intervals (Cis). Given evidence that waiting-list controls—and, to a lesser extent, treatment-as-usual—can inflate psychotherapy effect sizes, we prespecified psychoeducation or supportive psychotherapies as the primary control interventions (Cuijpers et al., 2024; Furukawa et al., 2014). A common heterogeneity parameter was assumed across all treatment comparisons, and the between-study variance (τ^2^) was reported for each outcome. We assessed the transitivity assumption by comparing the distribution of potential effect modifiers across treatment comparisons. Narrow inclusion criteria were also applied to ensure that studies comparing different sets of interventions were sufficiently similar to allow for valid indirect comparisons. Inconsistency assessment was conducted both globally and locally. Global inconsistency was examined using design-by-treatment interaction model, while local inconsistency was assessed by node-splitting methods. To explore potential sources of heterogeneity or inconsistency, we conducted a pre-specified network meta-regression for the primary outcome using the following potential effect modifiers: age, female proportion, treatment duration, and treatment resistance levels. Publication bias and small-study effects were assessed using comparison-adjusted funnel plots and Egger’s test when more than ten studies were available. We also assessed the RoB due to missing evidence in the estimates for the primary outcome (Chiocchia et al., 2021). Two sensitivity analyses were performed: one excluding studies involving group therapy, and another restricting the primary outcome analysis to studies with an actual 3-month follow-up.

## Results

### Study characteristics

Our searches resulted in 5518 potentially relevant citations (Supplementary eFigure 1). The complete search strategy and reasons for the exclusion of certain studies can be found in Supplementary appendix 2 and 3. After removing duplicates, we included 26 RCTs (Cather et al., 2005; Craig et al., 2018; Dellazizzo et al., 2021; Durham et al., 2003; Freeman et al., 2015; Garety et al., 2024; Haddock et al., 1998, 2009; Husain et al., 2017; Krakvik, Grawe, Hagen, & Stiles, 2013; Lee et al., 2013; Leff et al., 2013; Lewis et al., 2002; Liang et al., 2022; McLeod, Morris, Birchwood, & Dovey, 2007; Morrison et al., 2018; Mortan Sevi et al., 2020; Penn et al., 2009; Percie du Sert et al., 2018; Rathod et al., 2013; Shawyer et al., 2012; Trower et al., 2004; Valmaggia et al., 2005; Wahass & Kent, 1997; Wong, Ting, & Chen, 2019; Wykes et al., 2005). Supplementary eTable 1 shows the characteristics of included studies. These studies included 2273 participants with mean age of 39.32 years (4.09), and 65.02% were male. There were 396 participants in the AVATAR therapy arm (number of intervention arms [k] = 7) with mean age of 39.65 years (4.61), and 824 participants (k = 24) in the CBT arm with mean age of 38.70 years (4.51). The mean number of sessions was 7.3 (SD = 2.2) for AVATAR therapy and 15.4 (SD = 5.2) for CBT. The mean treatment duration was 10.7 (SD = 3.7) weeks for AVATAR therapy and 17.7 (SD = 9.0) weeks for CBT. Among the 26 RCTs, 7 studies (Dellazizzo et al., 2021; Krakvik et al., 2013; Liang et al., 2022; McLeod et al., 2007; Percie du Sert et al., 2018; Wahass & Kent, 1997; Wong et al., 2019) were open-label design and 19 studies were single blinded design (Cather et al., 2005; Craig et al., 2018; Durham et al., 2003; Freeman et al., 2015; Garety et al., 2024; Haddock et al., 1998, 2009; Husain et al., 2017; Lee et al., 2013; Leff et al., 2013; Lewis et al., 2002; Morrison et al., 2018; Mortan Sevi et al., 2020; Penn et al., 2009; Rathod et al., 2013; Shawyer et al., 2012; Trower et al., 2004; Valmaggia et al., 2005; Wykes et al., 2005). Other interventions included in this NMA were supportive psychotherapy (k = 7, n = 301), psychoeducation (k = 2, n = 36), social activity therapy (k = 1, n = 39), and treatment-as-usual (k = 16, n = 668). Overall, 17 studies provided post-intervention follow-up data for the primary outcome (AH severity). Among them, 11 studies (Craig et al., 2018; Dellazizzo et al., 2021; Durham et al., 2003; Freeman et al., 2015; Garety et al., 2024; Husain et al., 2017; Leff et al., 2013; Liang et al., 2022; Mortan Sevi et al., 2020; Penn et al., 2009; Percie du Sert et al., 2018) reported 3-month follow-up data, while the remaining 6 did not (Haddock et al., 2009; Lewis et al., 2002; Morrison et al., 2018; Shawyer et al., 2012; Trower et al., 2004; Valmaggia et al., 2005); for those studies, we used the data from the time point closest to 3 months. Among the 26 included studies, 21 (80.8%) defined treatment resistance at Level 1, 3 (11.5%) at Level 2, and 2 (7.7%) at Level 3.

### Quality of evidence

Overall risk of bias was Moderate in 13 studies and High in 7 studies. Ratings for individual domains for each study are provide in Supplementary eFigure 2 and 3. The proportions of studies with high, some concerns, and low ROB for the individual items of AVATAR trials were as follows: 0/26, 8/26, and 18/26 for randomization; 6/26, 20/26, and 0/6 for deviations from intended interventions; 1/26, 5/26, and 20/26 for missing outcome data; 0/26, 8/26, and 18/26 for measurements of outcomes; 0/26, 9/26, and 17/26 for selection of reported results. The potential risk of bias due to missing evidence was high for CBV versus TAU (Supplementary eFigure 43). The potential effect modifiers across treatment comparisons were assessed by visual inspection (Supplementary eFigure 35 to 38), suggesting that the transitivity assumption was reasonably met.

### Primary outcome: auditory hallucination severity

**Figure 2.**
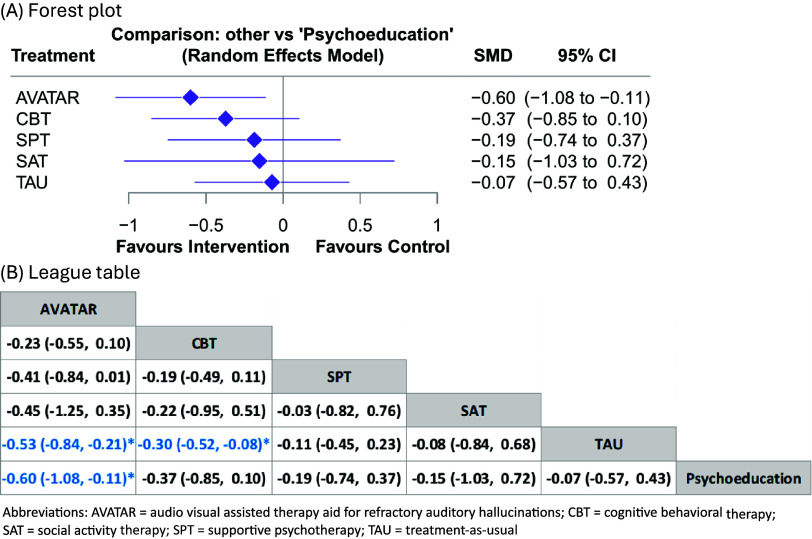
Comparisons of different treatments for severity of treatment-resistant auditory hallucinations. (a) Forest plot, (b) League table. Abbreviations: AVATAR, audio visual assisted therapy aid for refractory auditory hallucinations; CBT, cognitive behavioral therapy; SAT, social activity therapy; SPT, supportive psychotherapy; TAU, treatment-as-usual.

The network plots for all outcomes were provided in Supplementary eFigure 4 to 12. A total of 26 studies provided data for the NMA of the outcome of AH severity. When compared with psychoeducation (the reference arm), AVATAR therapy was associated with a significant improvement in AH severity (SMD = −0.60, 95% CI = −1.08 to −0.11, Figure 2a). In contrast, other interventions, including CBT, social activity therapy, treatment-as-usual, and supportive psychotherapy, did not show a significant difference compared to psychoeducation. However, when compared with CBT, AVATAR therapy did not demonstrate significantly higher efficacy (SMD = −0.23, 95% CI = −0.55 to 0.10, Figure 2b). Details of the network meta-analysis estimates are provided in Supplementary eTable 2 and 11. Overall, AVATAR therapy had an 81.0% probability of being the best intervention among the six, while CBT had a 4.0% probability (rank second) (Supplementary eFigure 17). A small study effect was detected (Egger’s test, *p* < 0.01, Supplementary eFigure 26). No global and local inconsistency were found (Supplementary Appendix 4).

**Figure 3.**
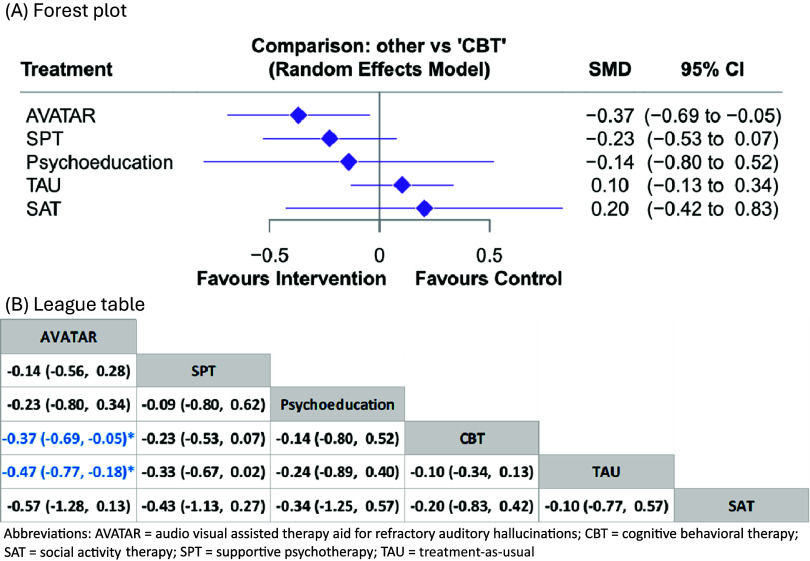
Comparisons of the long-term efficacy of different treatments for severity of treatment-resistant auditory hallucinations (3-month after treatment discontinuation). (a) Forest plot, (b) League table. Abbreviations: AVATAR, audio visual assisted therapy aid for refractory auditory hallucinations; CBT, cognitive behavioral therapy; SAT, social activity therapy; SPT, supportive psychotherapy; TAU, treatment-as-usual.

Seventeen studies provided data for the NMA of the outcome of post-intervention efficacy in AH severity (3-month after treatment discontinuation). AVATAR therapy showed a significantly better long-term efficacy in AH severity compared with CBT (SMD = −0.37, 95% CI = −0.69 to −0.05, Figure 3a, b). Other interventions, including supportive psychotherapy, psychoeducation, social activity therapy, and treatment-as-usual did not show a significant difference compared with CBT. Details of the network meta-analysis estimates are provided in Supplementary eTable 3 and 12. Overall, AVATAR therapy had a 58% probability of being the best intervention among the six interventions. (Supplementary eFigure 18). However, a small study effect was noted (Egger’s test, *p* < 0.01, Supplementary eFigure 27). No global and local inconsistency were found (Supplementary Appendix 5).

In meta-regression analyses (Supplementary eFigure 39 to 42), neither age, female proportion, study duration, nor resistance level was a potential effect modifier.

In sensitivity analysis excluding group therapy, AVATAR therapy was still associated with a significant improvement in AH severity when compared with psychoeducation (SMD = −0.55, 95% CI = −1.09 to −0.01, Supplementary eFigure 44). While, other interventions, including CBT, social activity therapy, treatment-as-usual, and supportive psychotherapy, did not show a significant difference compared to psychoeducation. Another sensitivity analysis of including studies with actual 3-month follow-up data for AH severity, we obtained a similar result (SMD = −0.46, 95%CI = −0.75 to −0.17, Supplementary efigure 45).

### Secondary outcomes

**Figure 4.**
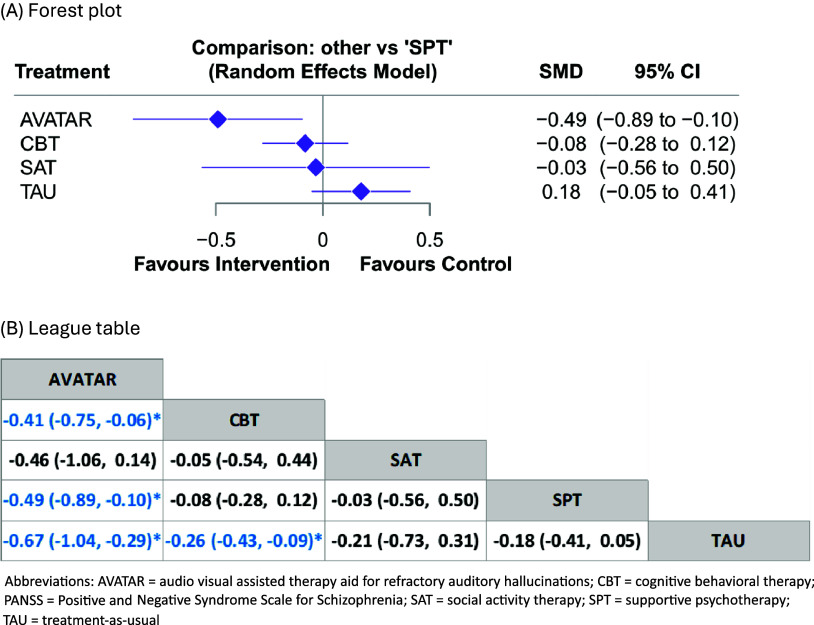
Comparisons of different treatments for overall psychotic symptoms measured by PANSS total score. (a) Forest plot, (b) League table. Abbreviations: AVATAR, audio visual assisted therapy aid for refractory auditory hallucinations; CBT, cognitive behavioral therapy; PANSS, Positive and Negative Syndrome Scale for Schizophrenia; SAT, social activity therapy; SPT, supportive psychotherapy; TAU, treatment-as-usual.

**Figure 5.**
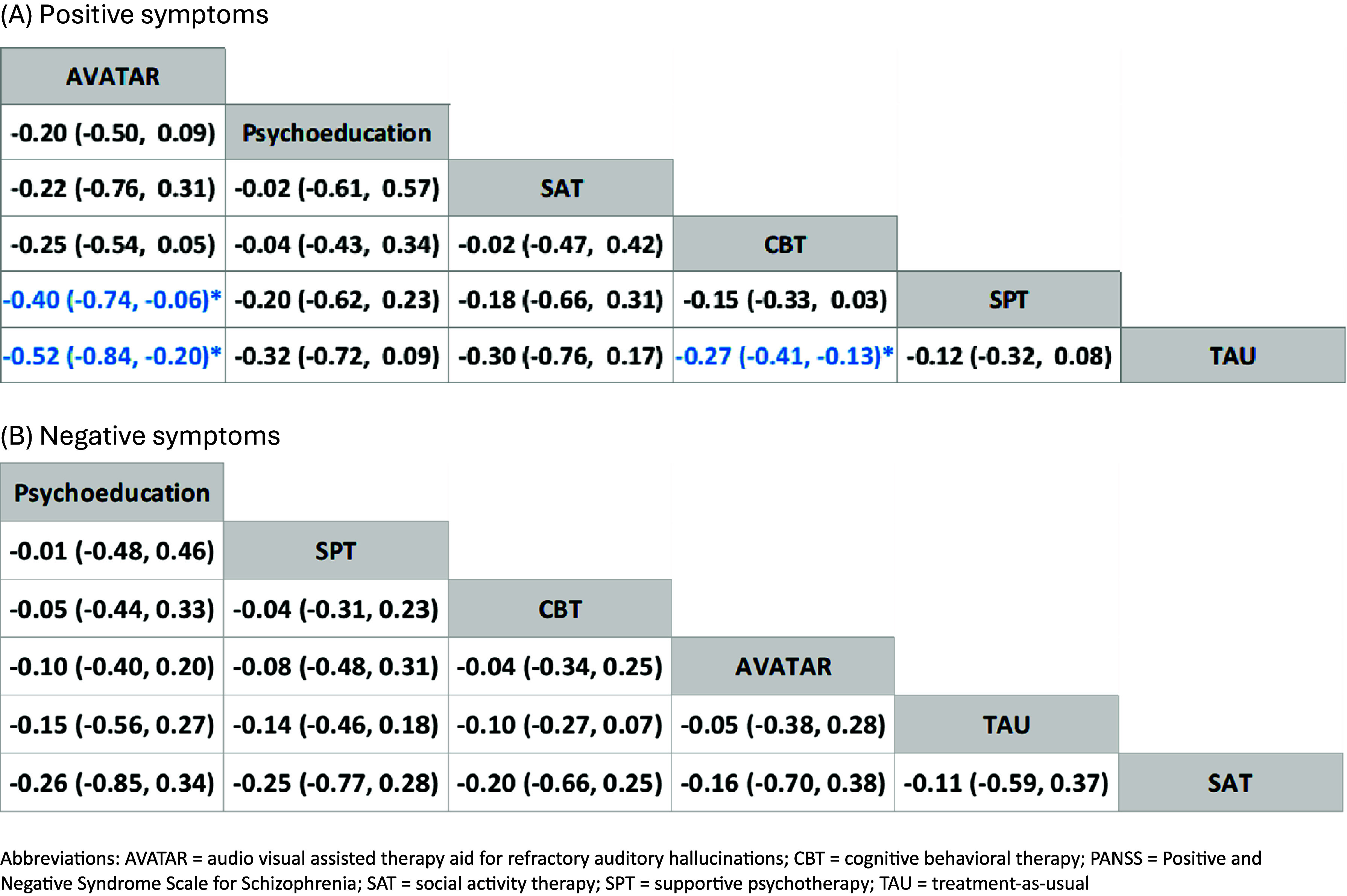
Comparisons of different treatments for positive and negative psychotic symptoms measured by PANSS. (a) Positive symptoms, (b) Negative symptoms. Abbreviations: AVATAR, audio visual assisted therapy aid for refractory auditory hallucinations; CBT, cognitive behavioral therapy; PANSS, Positive and Negative Syndrome Scale for Schizophrenia; SAT, social activity therapy; SPT, supportive psychotherapy; TAU, treatment-as-usual.

For overall psychotic symptoms, AVATAR therapy was associated with significant improvement in overall psychotic symptoms measured by PANSS total score (Figure 4a, SMD = −0.49, 95%CI = −0.89 to −0.10) than supportive psychotherapy, and further outperformed than other interventions, particularly CBT (SMD = −0.41, 95% CI = −0.75 to −0.06) (Figure 4a, b). Regarding positive symptoms, AVATAR therapy was associated with significantly better efficacy than supportive psychotherapy (Figure 5a, SMD = −0.40, 95%CI = −0.74 to −0.06) and TAU (Figure 5a, SMD = −0.52, 95%CI = −0.84 to −0.20). In addition, CBT was also associated with significantly better efficacy than TAU (Figure 5a, SMD = −0.27, 95%CI = −0.41 to −0.13). When assessing negative symptoms, no significant difference was found between these six interventions (Figure 5b). Details of the network meta-analysis estimates are provided in Supplementary eTable 4 to 6 and Supplementary eTable 13 to 15. AVATAR therapy had the highest probability of being the best intervention regarding overall psychotic symptoms (92.0%, Supplementary eFigure 19) and positive symptoms (67.0%, Supplementary eFigure 20), but not negative symptoms (Supplementary eFigure 21).

Regarding depressive symptoms, anxiety symptoms, and quality of life (Supplementary eFigure 13 to 15), when comparing to psychoeducation, no intervention demonstrated a significant difference in efficacy. Compared to CBT, AVATAR therapy did not have a significant better efficacy in depressive symptoms, anxiety symptoms, nor quality of life. Details of the network meta-analysis estimates are provided in Supplementary eTable 7 to 9 and 16 to 18. The probabilities of being the best intervention regarding depressive symptoms, anxiety symptoms, quality of life were presented in Supplementary eFigure 22 to 24.

Regarding all-cause discontinuation (Supplementary eFigure 16, Supplementary eTable 10, and Supplementary eTable 19), no significant difference was noted among these interventions. AVATAR therapy has the lowest probability (4.2%) of being the highest-risk treatment for all-cause discontinuation (Supplementary eFigure 25). No obvious publication bias, global inconsistency, or local inconsistency was noted for all secondary outcomes (Supplementary eFigure 28 to 34, Supplementary Appendix 6 to 12).

## Discussion

In this network meta-analytic study, it was found that AVATAR therapy had a relatively better effect on AH severity compared to CBT based on ranking, although it was not statistically significant when considering effect estimates. Among the other six non-pharmacological interventions included in our analysis, AVATAR therapy and CBT ranked first and second, respectively, with their effect sizes showing a clear difference from the other interventions, including psychoeducation, supportive psychotherapy, social activity therapy, and treatment as usual. Regarding postintervention efficacy in AH severity, AVATAR therapy demonstrated a significantly better efficacy than CBT. In addition, AVATAR therapy also outperformed CBT in overall psychotic symptoms. Regarding positive symptoms, negative symptoms, depressive symptoms, anxiety symptoms, and quality of life, no significant difference was noted between AVATAR therapy and CBT. Finally, both AVATAR therapy and CBT were well-accepted.

Avatar therapy is also based on CBT principles, incorporating a personalized digital “hallucinated voice” and a therapist-controlled avatar character to interact with the patient, helping them learn how to confront or engage with their hallucinations. Compared to CBT, which relies on cognitive restructuring and behavioral changes and involves more abstract concepts, Avatar therapy provides more immediate and tangible feedback for patients. This is especially important as individuals with medication-resistant AH often have comorbid negative symptoms or cognitive impairments that limit their ability to engage with abstract therapies (Tarrier & Wykes, 2004; Thomas et al., 2011). AVATAR therapy addresses this challenge by providing not only greater reduction in AH severity than CBT at treatment endpoint, but also sustained benefits following treatment discontinuation. Importantly, AVATAR therapy typically requires 5–12 fewer sessions than CBT, suggesting potential advantages in both treatment efficiency and cost-effectiveness.

In terms of overall psychotic symptoms measured by PANSS, Avatar therapy has demonstrated greater efficacy compared to CBT. For positive symptoms, although Avatar therapy primarily focuses on AH, it still shows a small to moderate effect size compared to CBT. This suggests that during the Avatar therapy process, not only AH but also related delusions and behaviors may be addressed and improved. Notably, when compared with TAU, AVATAR therapy showed a moderate effect size with SMD of −0.52, and CBT only had a small effect size with SMD of 0.23. The effect size of CBT estimated in our study is very close to the effect size reported in previous meta-analytic studies (SMD = −0.31, 95%CI = −0.43 to −0.19), which further strengthens the credibility of our findings. Looking back to previous RCTs for SSD the pooled effect size of antipsychotics for positive symptoms was 0.45 (SMD = −0.45, 95% credible intervals = −0.40 to −0.50) (Leucht et al., 2017). Although the results of psychological interventions and pharmacological interventions cannot be directly compared due to differences in the populations (psychological interventions are add-ons to medication, while participants in pharmacological RCTs are not medication-resistant), (Bighelli et al., 2020) we can still use this to anticipate the extent of improvement in patients with medication-resistant AH after receiving AVATAR therapy or CBT. In future clinical trials of AVATAR therapy, it may be designed to target delusions or overall positive symptoms to evaluate the effectiveness of actual interactions and feedback on these psychotic symptoms. Regarding negative symptoms, since neither therapy in its current form specifically targets them, there is no significant difference between AVATAR therapy and CBT. In fact, neither Avatar therapy nor CBT ranks among the most effective interventions for negative symptoms. Given the strong association between negative symptoms and long-term functional outcomes (Marder & Galderisi, 2017), future adaptations of AVATAR therapy could extend beyond voice-dialogue work to target avolition, anhedonia, asociality, and diminished expressivity by integrating behavioral activation (goal-setting and graded tasks), avatar-supported reinforcement, and avatar-based social skills role-play with repeated low-threat social exposures. Future trials could also emphasize functional/ecological endpoints (e.g., social participation, vocational engagement, daily activity) using real-world behavioral metrics, while tailoring session structure for prominent negative symptoms (shorter, more structured sessions with reduced cognitive load and frequent behavioral prompts) to improve engagement and generalization. Interestingly, research indicates that the frequency of dyadic interactions between the avatar (who is also the therapist) and the patient is positively correlated with therapeutic outcomes (Hudon et al., 2024). The establishment of a therapeutic alliance and frequent interaction (suggesting a lower level of negative symptoms) are associated with better treatment effects on positive symptoms (Hudon et al., 2024). A lower level of negative symptoms is also associated with better treatment outcomes for psychotic symptoms, as observed in CBT research (Tarrier & Wykes, 2004; Thomas et al., 2011). Nowadays, although AVATAR therapy is not specifically used for treating negative symptoms, several studies have been published on virtual reality-assisted therapy for negative symptoms and social function, showing positive outcomes (Cella et al., 2022; Hosgelen, Guneri, Erdeniz, & Alptekin, 2024).

Although AVATAR therapy ranked favorably, the magnitude of between-intervention differences was generally small. Most effect estimates were in the small range and several comparisons did not reach statistical significance, suggesting that clinically meaningful improvement may occur for some patients but should not be assumed to be large or universal.Several explanations may account for the generally small observed effects. First, the included samples largely comprised individuals with medication-resistant AH, in whom residual symptom severity, chronicity, and cognitive/negative symptom burden may constrain the achievable effect size (i.e., a “ceiling” on symptom change within short trials). Second, despite differing formats, many psychological interventions for AH may share overlapping active ingredients and non-specific therapeutic factors—such as normalization of voice experiences, cognitive reappraisal, emotion regulation, exposure to feared voice-related cues, and a structured therapeutic alliance—thereby yielding only modest incremental differences between treatments. Clinically, this supports a cautious interpretation: AVATAR therapy may be considered a promising alternative or adjunct, but decisions should prioritize feasibility, patient preference, and access, and avoid overgeneralizing modest average effects to all patients.

## Limitations

Our study has some limitations. First, we detected small study effects for the primary outcome. In addition, the potential high RoB due to missing evidence was observed for the comparison between CBT versus TAU. Therefore, the inflated effect estimate for CBT may biased indirect comparisons involving CBT. Second, due to the characteristics of psychotherapy-related clinical trials, blinding was typically restricted to single-blind or assessor-blind methodologies. Expectancy and novelty effects may also have influenced outcomes. Because participants and therapists cannot be blinded in psychotherapy trials, VR-based interventions such as AVATAR therapy may elicit higher treatment credibility and expectations (“novelty effect”), which can enhance engagement and perceived improvement. This could inflate post-intervention effects on outcomes that are more expectation-sensitive (e.g., ratings of voice severity/distress) and may partially contribute to differences observed between AVATAR therapy and CBT. Although several trials used assessor-blinded outcome ratings, expectancy-related effects cannot be fully ruled out. Third, although CBT or AVATAR therapy follows certain principles, the way these therapies are implemented, and their effectiveness can vary across different regions. This variation may also contribute to heterogeneity. Fourth, the degree of medication-resistant AH included in our analysis may vary, contributing to heterogeneity. In most trials, descriptions of medication resistance were relatively limited. Generally, participants were categorized as level 1 if they remained stable under outpatient follow-up and medication treatment but continued to experience AH. A few trials included individuals who had persistent AH despite undergoing full trials with two different medications (level 2) or those with clozapine resistance (level 3). Fifth, the number of trials and sample sizes for AVATAR therapy are relatively smaller compared to CBT (396 vs 1,877 patients). While our inconsistency assessments (design-by-treatment interaction model and node-splitting method) confirmed that this imbalance did not significantly bias the point estimates (all *p*-values >0.05 for inconsistency tests), the smaller sample size resulted in wider confidence intervals for AVATAR-related comparisons, which may reduce statistical power to detect potential differences. Sixth, trials of CBT generally target positive symptoms as a whole, which include AH but also other symptoms (Fassler et al., 2024). However, these trials do include AH-related measurements, the treatment approach may differ slightly due to the broader focus. This difference in treatment targets could be a potential factor influencing the comparison of AH severity outcomes between AVATAR therapy and CBT. Finally, our analysis revealed no significant differences in depressive symptoms, anxiety symptoms, or quality of life between active interventions and psychoeducation. This null finding may be attributed to the fact that AVATAR therapy and CBT protocols in the included studies primarily focused on AD rather than emotional symptoms in medication-resistant patients.

## Conclusion

AVATAR therapy, a CBT-based intervention enhanced with virtual reality, shows comparable or slightly greater efficacy than CBT for medication-resistant auditory hallucinations, with sustained benefits after treatment. Clinically, it offers a promising adjunct to pharmacotherapy. Future research should confirm its long-term effectiveness, evaluate cost-efficiency, and develop scalable remote delivery models to improve accessibility.

## Supporting information

10.1017/S0033291726104127.sm001 Hsu et al. supplementary material Hsu et al. supplementary material

## Supplementary material

The supplementary material for this article can be found at http://doi.org/10.1017/S0033291726104127.
