# Supplementary material for: AVATAR versus cognitive-behavioral therapy for medication-resistant auditory hallucination: a systematic review and network meta-analysis
Source: Psychol Med. 2026 Apr 13;56:e107. doi: 10.1017/S0033291726104127 (PMC13079228; doi:10.1017/S0033291726104127)
Supplement: Hsu et al. supplementary material [file S0033291726104127sup001.docx]

**AVATAR versus cognitive-behavioral therapy for treatment-resistant auditory hallucination: systematic review and meta-analysis**

eFigure 1. Study flow chart

eFigure 2. Risk of bias plot

eFigure 3. Summary of risk of bias

eFigure 4. Network plot for severity of treatment-resistant auditory hallucinations

eFigure 5. Network plot for long-term follow-up effects on treatment-resistant auditory hallucinations

eFigure 6. Network plot for overall psychotic symptoms measured by PANSS total score

eFigure 7. Network plot for positive psychotic symptoms measured by PANSS

eFigure 8. Network plot for negative psychotic symptoms measured by PANSS

eFigure 9. Network plot for depressive symptoms

eFigure 10. Network plot for anxiety symptoms

eFigure 11. Network plot for quality of life

eFigure 12. Network plot for all-cause discontinuation

eFigure 13. Comparisons of different treatments for depressive symptoms

eFigure 14. Comparisons of different treatments for anxiety symptoms

eFigure 15. Comparisons of different treatments for quality of life

eFigure 16. Comparisons of different treatments for all-cause discontinuation

eFigure 17. Probability of being best treatment for severity of treatment-resistant auditory hallucinations

eFigure 18. Probability of being best treatment for long-term follow-up effects on treatment-resistant auditory hallucinations

eFigure 19. Probability of being best treatment for overall psychotic symptoms measured by PANSS

eFigure 20. Probability of being best treatment for positive psychotic symptoms measured by PANSS

eFigure 21. Probability of being best treatment for negative psychotic symptoms measured by PANSS

eFigure 22. Probability of being best treatment for depressive symptoms

eFigure 23. Probability of being best treatment for anxiety symptoms

eFigure 24. Probability of being best treatment for quality of life

eFigure 25. Probability of being best treatment for all-cause discontinuation

eFigure 26. Funnel plot of severity of auditory hallucinations

eFigure 27. Funnel plot of long-term follow-up effects on treatment-resistant auditory hallucinations

eFigure 28. Funnel plot of overall psychotic symptoms

eFigure 29. Funnel plot of positive psychotic symptoms

eFigure 30. Funnel plot of negative psychotic symptoms

eFigure 31. Funnel plot of depressive symptoms

eFigure 32. Funnel plot of anxiety symptoms

eFigure 33. Funnel plot of quality of life

eFigure 34. Funnel plot of all-cause discontinuation

eFigure 35. Visual inspection of transitivity assumption for potential effect modifier: age

eFigure 36. Visual inspection of transitivity assumption for potential effect modifier: female proportion

eFigure 37. Visual inspection of transitivity assumption for potential effect modifier: study duration

eFigure 38. Visual inspection of transitivity assumption for potential effect modifier: resistance level

eFigure 39. Network meta-regression for potential effect modifier: age

eFigure 40. Network meta-regression for potential effect modifier: female proportion

eFigure 41. Network meta-regression for potential effect modifier: study duration

eFigure 42. Network meta-regression for potential effect modifier: resistance level

eFigure 43. The evaluation of risk of bias due to missing evidence for the primary outcome

eFigure 44. Sensitivity analysis of excluding group therapy

eFigure 45. Sensitiviy analysis of the studies with acutal 3-month follow-up data on the primary outcome

eTable 1. Demographics and clinical characteristics of the included studies

eTable 2. Details of network meta-analysis estimates for severity of treatment-resistant auditory hallucinations

eTable 3. Details of network meta-analysis estimates for long-term follow-up effects on treatment-resistant auditory hallucinations

eTable 4. Details of network meta-analysis estimates for overall psychotic symptoms measured by PANSS

eTable 5. Details of network meta-analysis estimates for positive psychotic symptoms measured by PANSS

eTable 6. Details of network meta-analysis estimates for negative psychotic symptoms measured by PANSS

eTable 7. Details of network meta-analysis estimates for depressive symptoms

eTable 8. Details of network meta-analysis estimates for anxiety symptoms

eTable 9. Details of network meta-analysis estimates for quality of life

eTable 10. Details of network meta-analysis estimates for all-cause discontinuation

eTable 11. SUCRA value of each treatment for severity of auditory hallucinations

eTable 12. SUCRA value of each treatment for long-term follow-up effects on treatment-resistant auditory hallucinations

eTable 13. SUCRA value of each treatment for overall psychotic symptoms measured by PANSS

eTable 14. SUCRA value of each treatment for positive psychotic symptoms measured by PANSS

eTable 15. SUCRA value of each treatment for negative psychotic symptoms measured by PANSS

eTable 16. SUCRA value of each treatment for depressive symptoms

eTable 17. SUCRA value of each treatment for anxiety symptoms

eTable 18. SUCRA value of each treatment for quality of life

eTable 19. SUCRA value of each treatment for all-cause discontinuation

Appendix 1. The PRISMA

Appendix 2. The complete search strategies

Appendix 3. Reasons for exclusion

Appendix 4. Node-splitting and global inconsistency results for severity of auditory hallucinations

Appendix 5. Node-splitting and global inconsistency results for long-term follow-up effects on treatment-resistant auditory hallucinations

Appendix 6. Node-splitting and global inconsistency results for overall psychotic symptoms measured by PANSS

Appendix 7. Node-splitting and global inconsistency results for positive psychotic symptoms measured by PANSS

Appendix 8. Node-splitting and global inconsistency results for negative psychotic symptoms measured by PANSS

Appendix 9. Node-splitting and global inconsistency results for depressive symptoms

Appendix 10. Node-splitting and global inconsistency results for anxiety symptoms

Appendix 11. Node-splitting and global inconsistency results for quality of life

Appendix 12. Node-splitting and global inconsistency results for all-cause discontinuation

eFigure 1. PRISMA 2020 flow diagram for new systematic reviews which included searches of databases and registers only.

**Identification of studies via databases and registers**

Records identified from

Embase (n= 926)

PubMed (n= 2912)

CENTRAL (n= 1098)

PsycINFO (n= 571)

ClinicalTrials.gov (n= 11) :

---------------------------------

Databases (n = 5518)

Records removed *before screening*: Duplicate records removed (n = 1430)

**Identification**

Records excluded title and abstract (n = 4008)

Records screened

(n = 4088)

**Screening**

Reports not retrieved

(n = 0)

Reports sought for retrieval

(n = 80)

Reports excluded: (n=61)

No outcome of interest (n=25)

No outcome report or incomplete data (n=5)

Conference abstract, protocol, review, or comment (n=19)

Not meet inclusive criteria (n=7)

Not directly interact with therapists (n=4)

Duplicated data (n=1)

Reports assessed for eligibility

(n = 80)

**Included**

Studies included in review

(n = 26)

Reports of included studies

(n = 26)

Hand search (n = 7)

*From:*  Page MJ, McKenzie JE, Bossuyt PM, Boutron I, Hoffmann TC, Mulrow CD, et al. The PRISMA 2020 statement: an updated guideline for reporting systematic reviews. BMJ 2021;372:n71. doi: 10.1136/bmj.n71

eFigure 2. Risk of bias plot


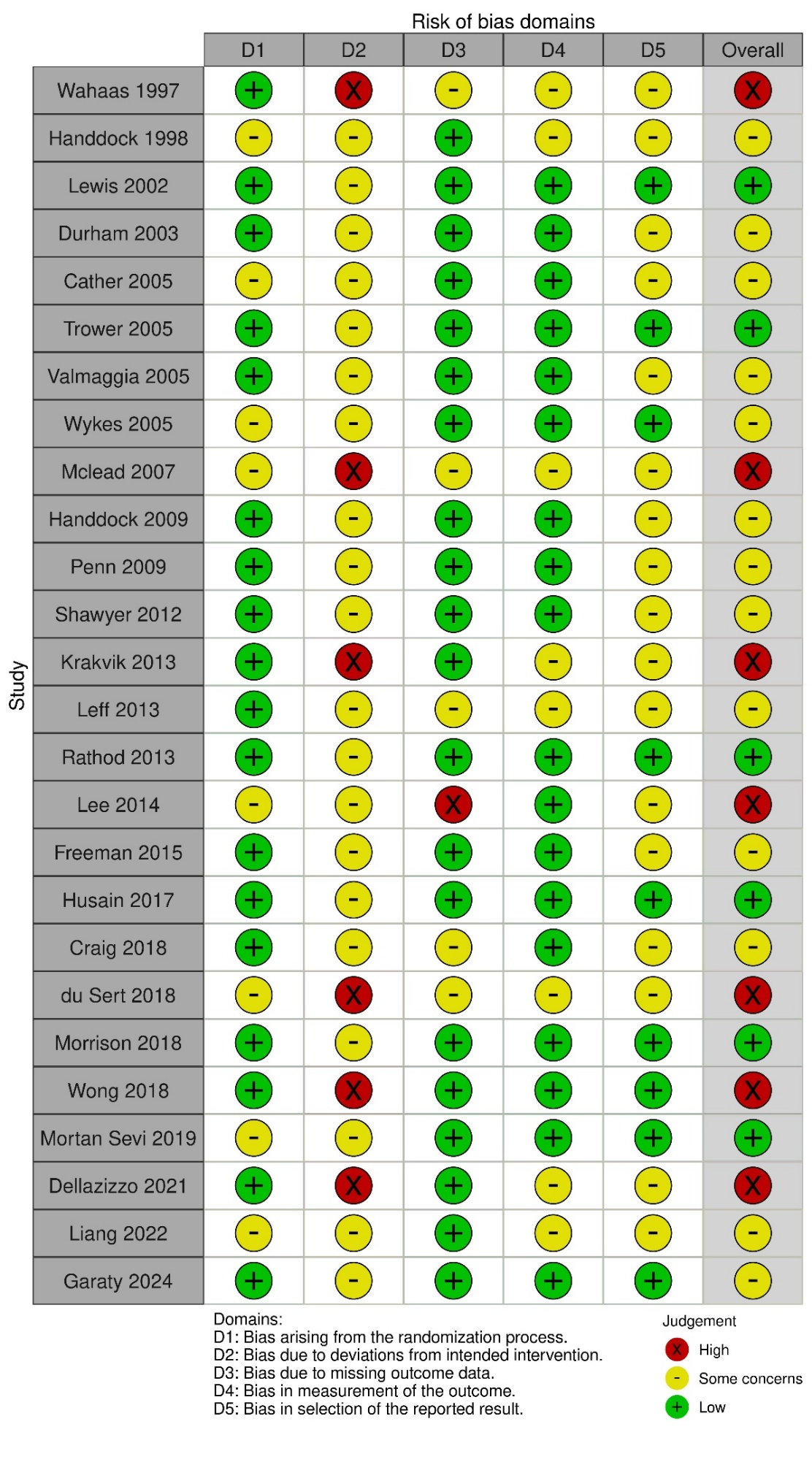


eFigure 3. Summary of risk of bias


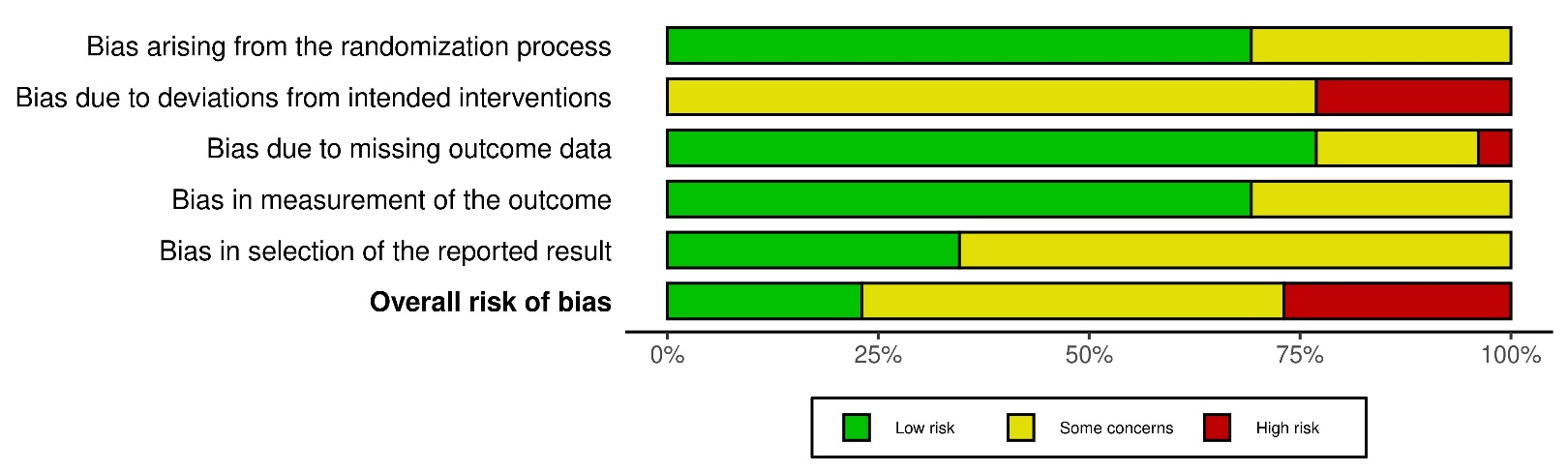


eFigure 4. Network plot for severity of treatment-resistant auditory hallucinations


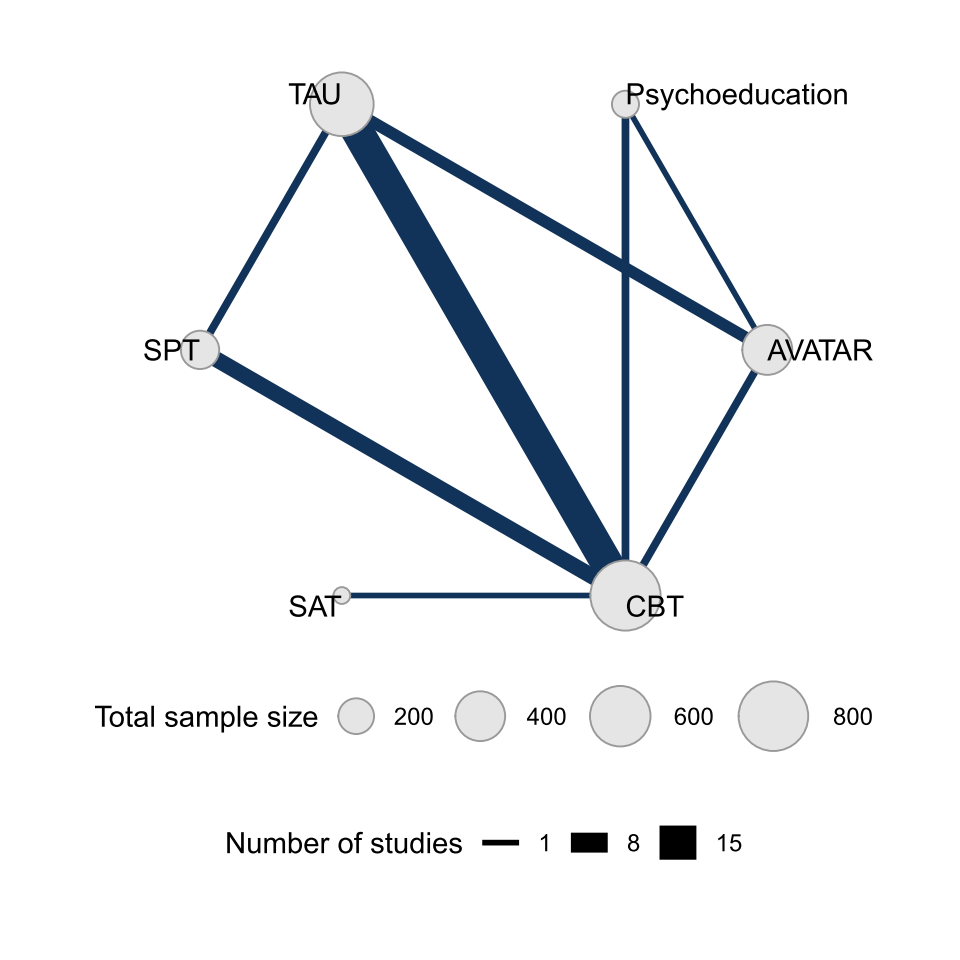


Abbreviations: AVATAR = audio visual assisted therapy aid for refractory auditory hallucinations; CBT = cognitive behavioral therapy; SAT = social activity therapy; SPT = supportive psychotherapy; TAU = treatment-as-usual

eFigure 5. Network plot for long-term follow-up effects on treatment-resistant auditory hallucinations


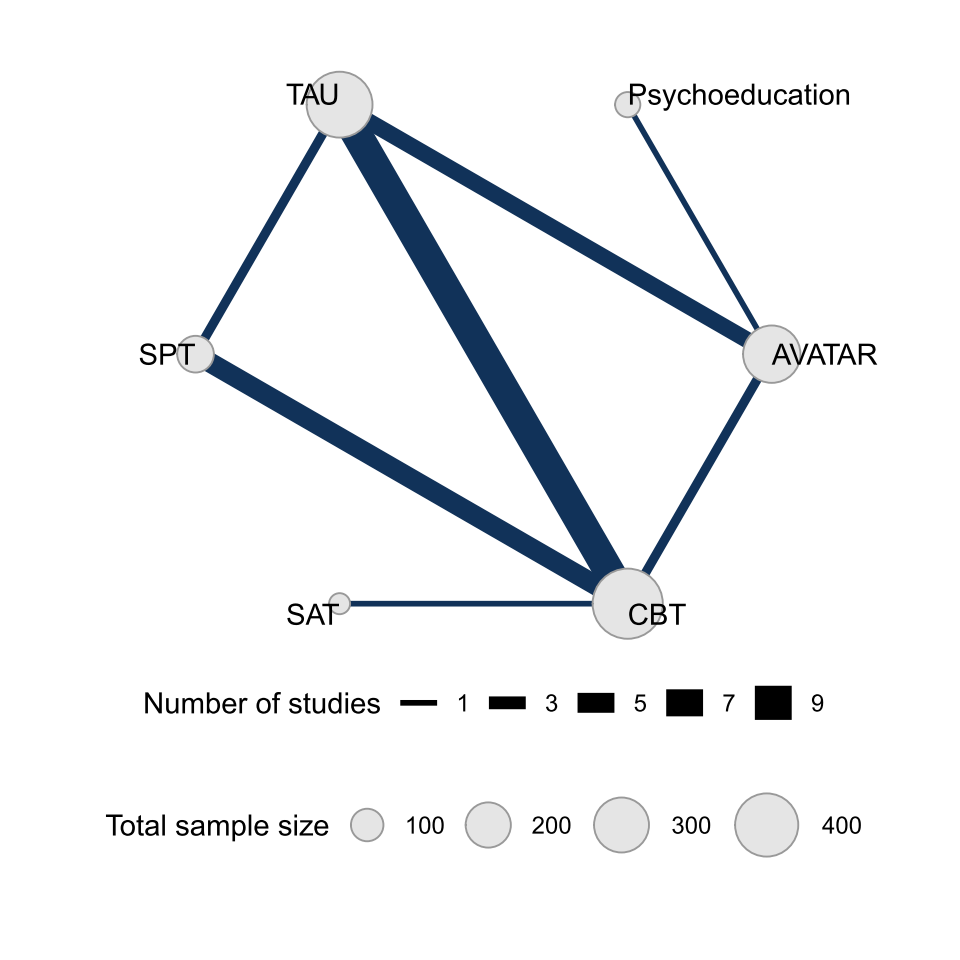


Abbreviations: AVATAR = audio visual assisted therapy aid for refractory auditory hallucinations; CBT = cognitive behavioral therapy; SAT = social activity therapy; SPT = supportive psychotherapy; TAU = treatment-as-usual

eFigure 6. Network plot for overall psychotic symptoms measured by PANSS total score


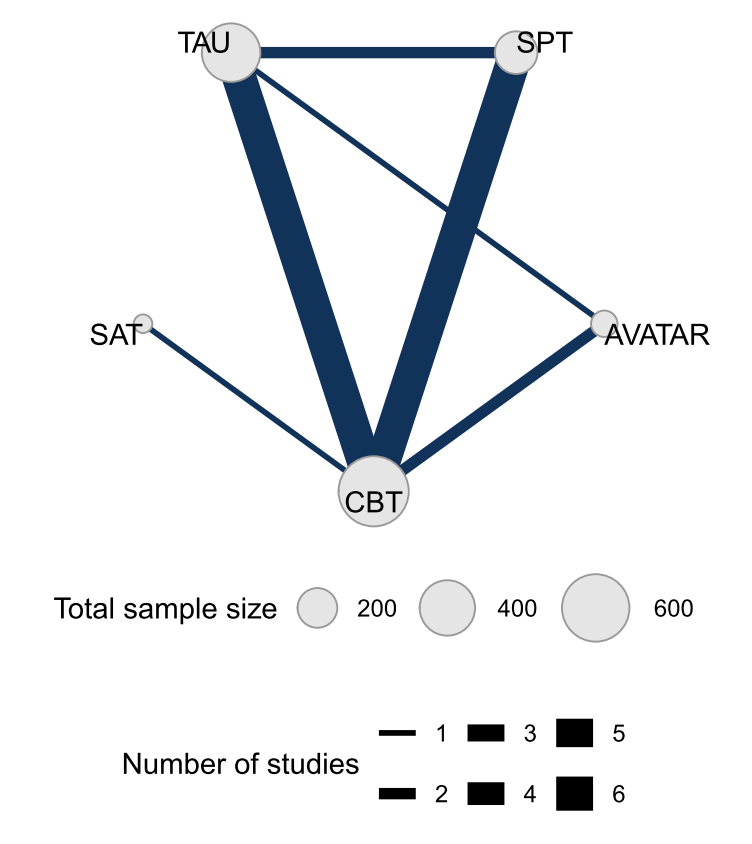


Abbreviations: AVATAR = audio visual assisted therapy aid for refractory auditory hallucinations; CBT = cognitive behavioral therapy; PANSS = Positive and Negative Syndrome Scale for Schizophrenia; SAT = social activity therapy; SPT = supportive psychotherapy; TAU = treatment-as-usual

eFigure 7. Network plot for positive psychotic symptoms measured by PANSS


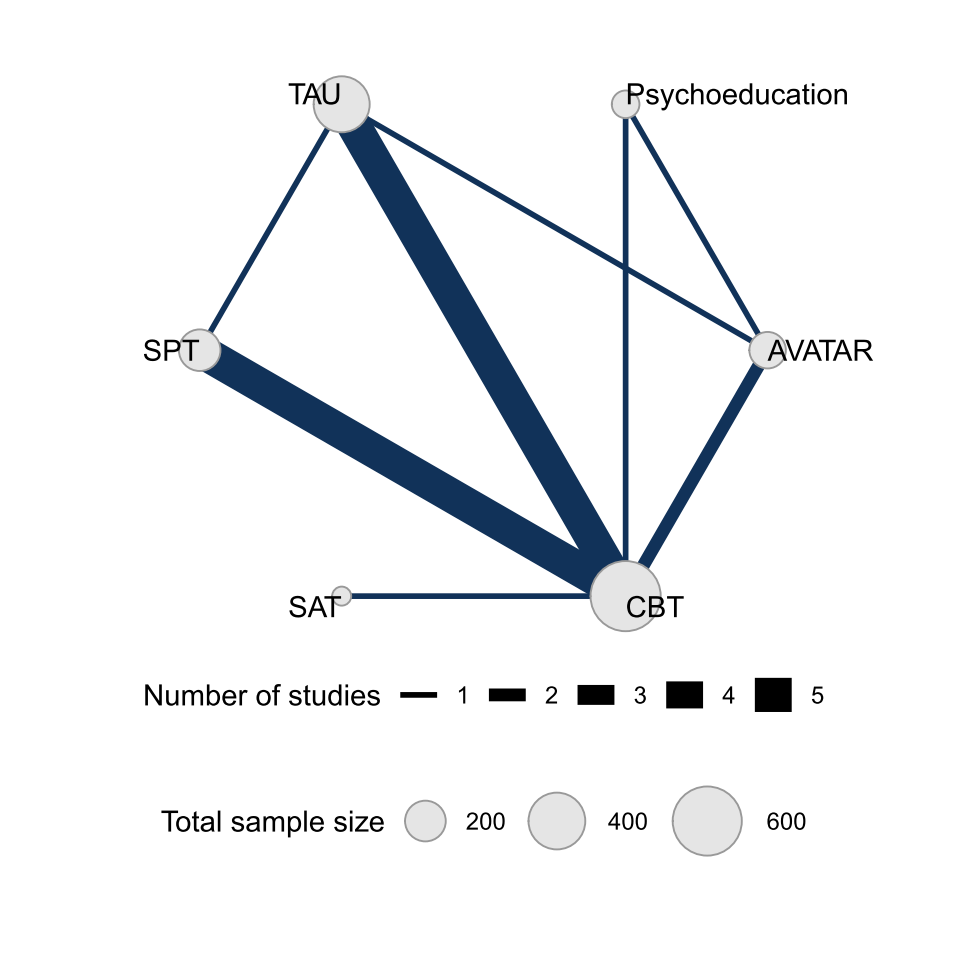


Abbreviations: AVATAR = audio visual assisted therapy aid for refractory auditory hallucinations; CBT = cognitive behavioral therapy; PANSS = Positive and Negative Syndrome Scale for Schizophrenia; SAT = social activity therapy; SPT = supportive psychotherapy; TAU = treatment-as-usual

eFigure 8. Network plot for negative psychotic symptoms measured by PANSS


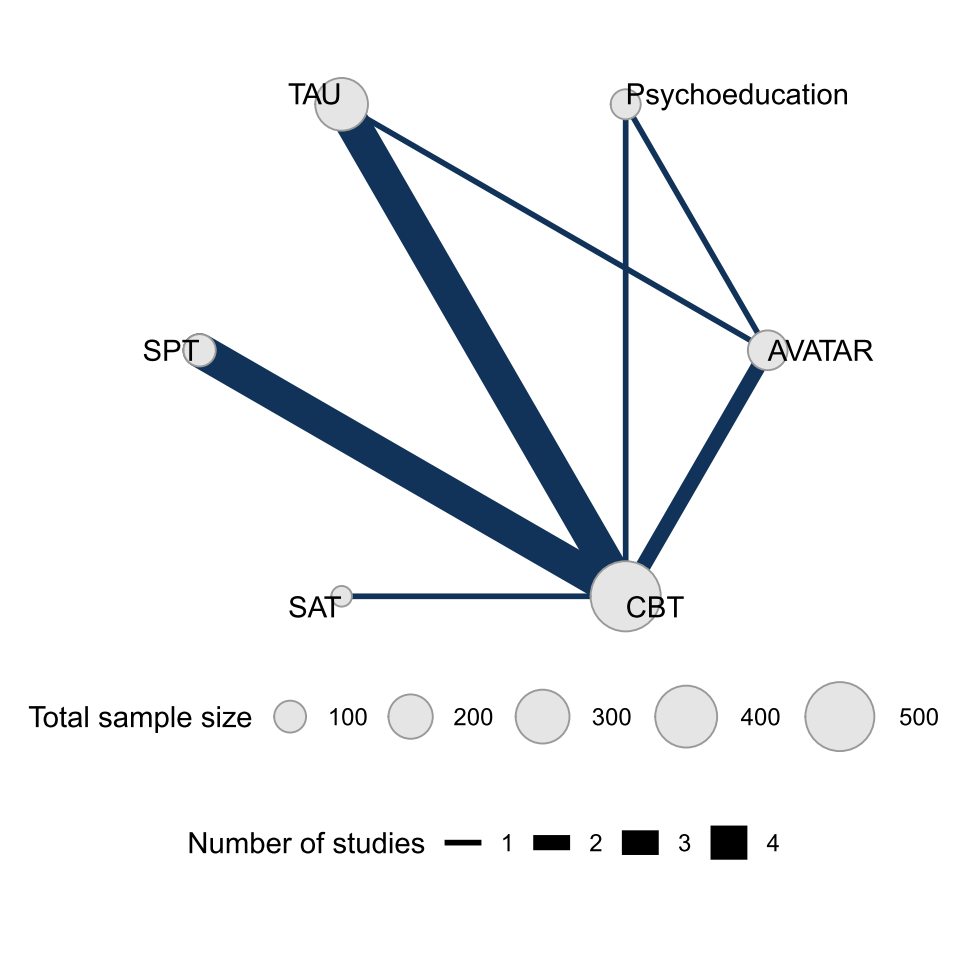


Abbreviations: AVATAR = audio visual assisted therapy aid for refractory auditory hallucinations; CBT = cognitive behavioral therapy; PANSS = Positive and Negative Syndrome Scale for Schizophrenia; SAT = social activity therapy; SPT = supportive psychotherapy; TAU = treatment-as-usual

eFigure 9. Network plot for depressive symptoms


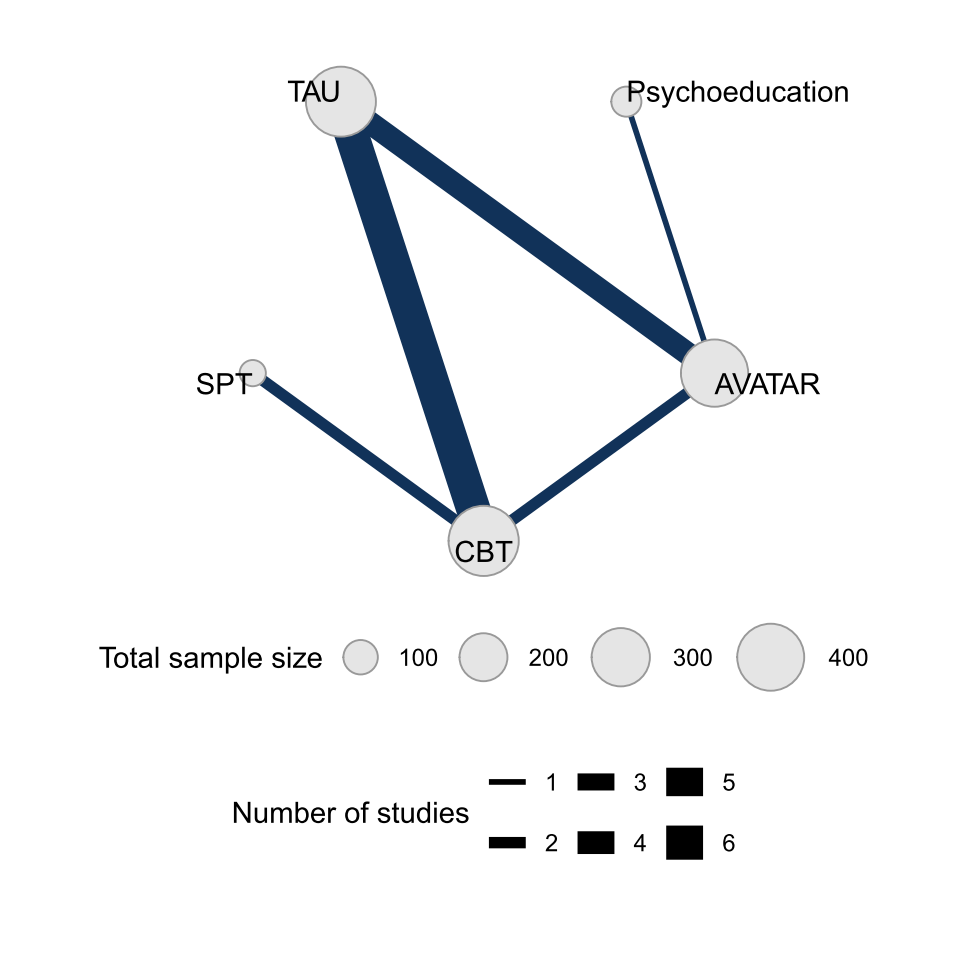


Abbreviations: AVATAR = audio visual assisted therapy aid for refractory auditory hallucinations; CBT = cognitive behavioral therapy; SAT = social activity therapy; SPT = supportive psychotherapy; TAU = treatment-as-usual

eFigure 10. Network plot for anxiety symptoms


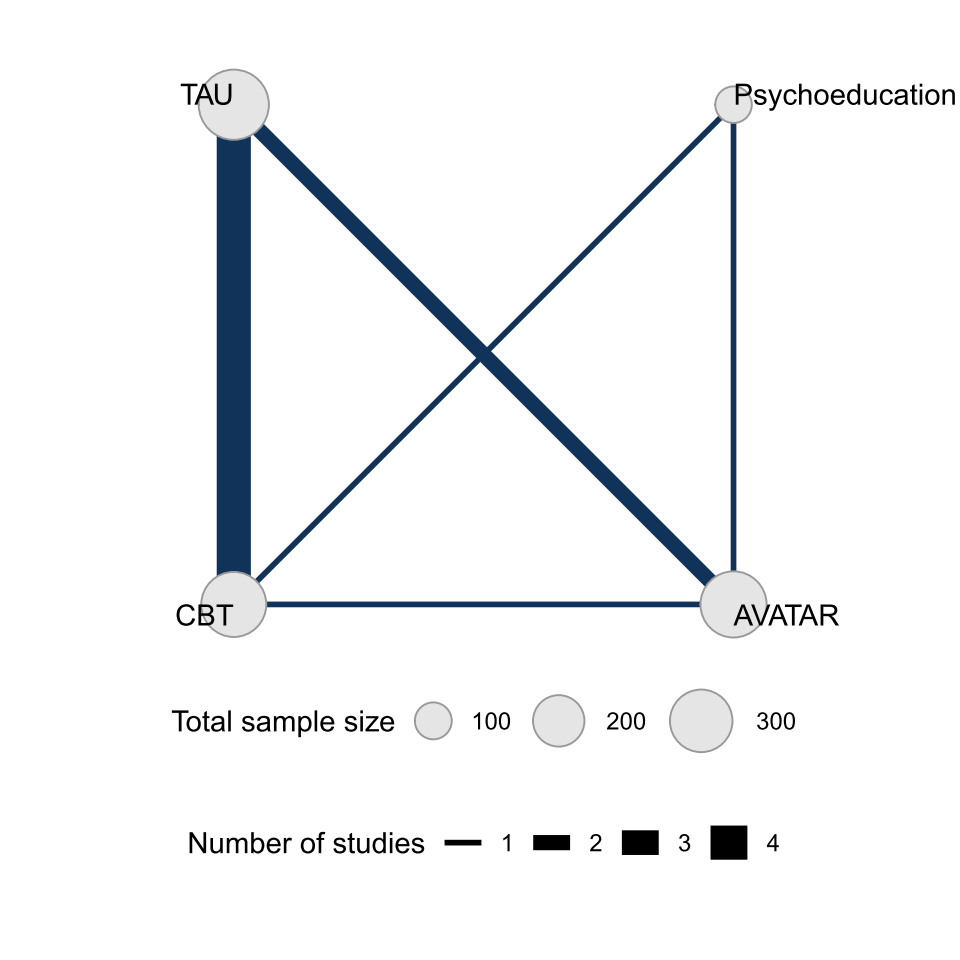


Abbreviations: AVATAR = audio visual assisted therapy aid for refractory auditory hallucinations; CBT = cognitive behavioral therapy; TAU = treatment-as-usual

eFigure 11. Network plot for quality of life


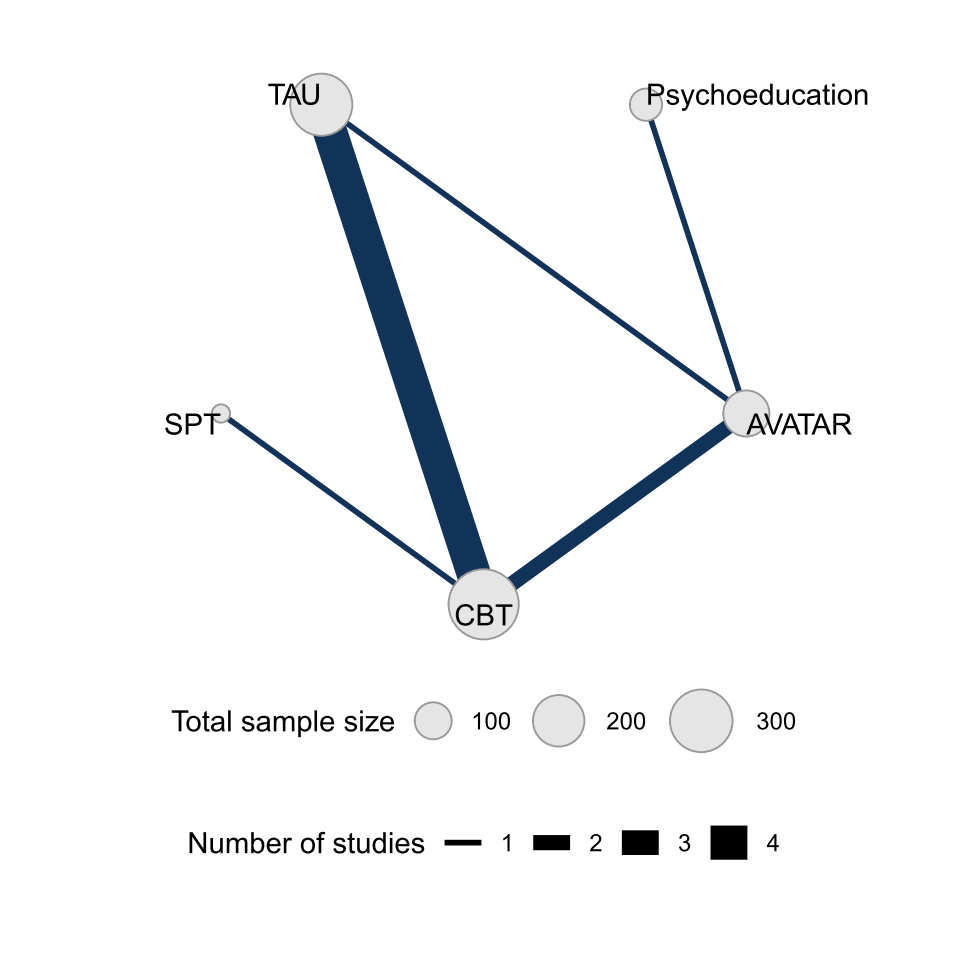


Abbreviations: AVATAR = audio visual assisted therapy aid for refractory auditory hallucinations; CBT = cognitive behavioral therapy; TAU = treatment-as-usua

eFigure 12. Network plot for all-cause discontinuation


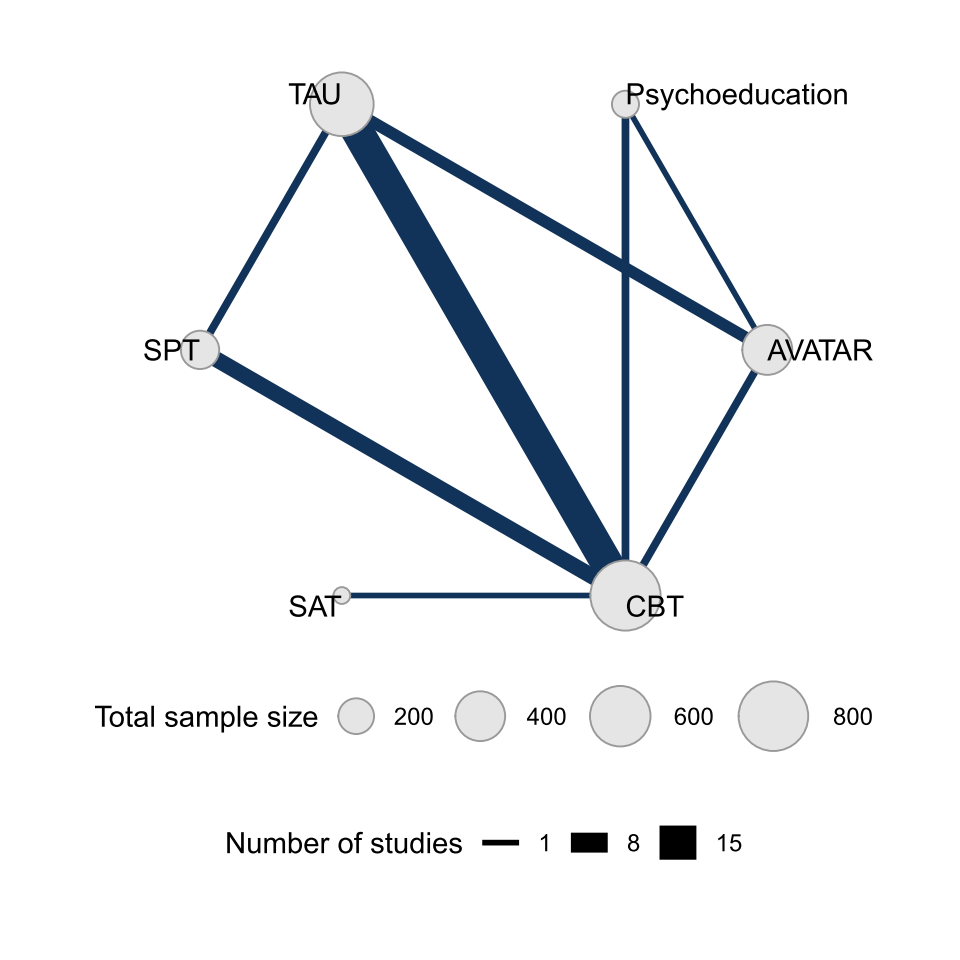


Abbreviations: AVATAR = audio visual assisted therapy aid for refractory auditory hallucinations; CBT = cognitive behavioral therapy; SAT = social activity therapy; SPT = supportive psychotherapy; TAU = treatment-as-usual

eFigure 13. Comparisons of different treatments for depressive symptoms

| 1. Forest plot |
| --- |
| 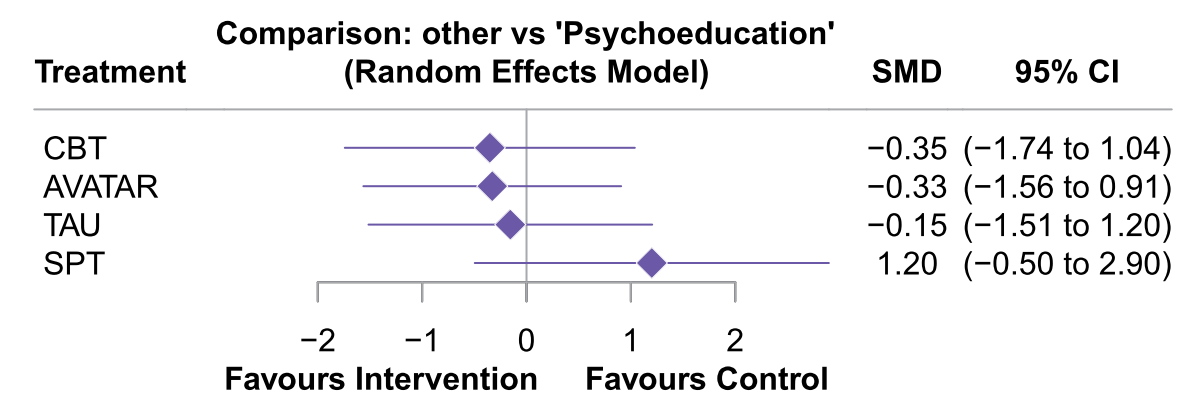 |
| 1. League table |
| 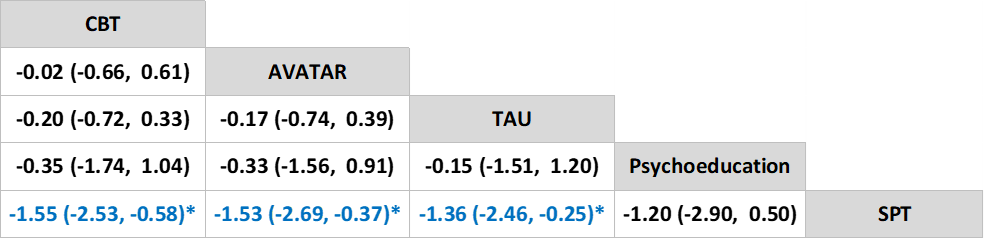 |

eFigure 14. Comparisons of different treatments for anxiety symptoms

| 1. Forest plot |
| --- |
| 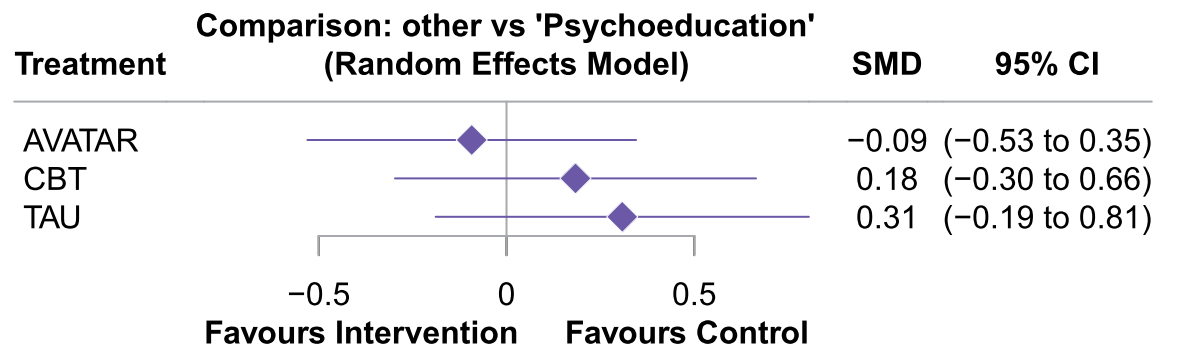 |
| 1. League table |
| 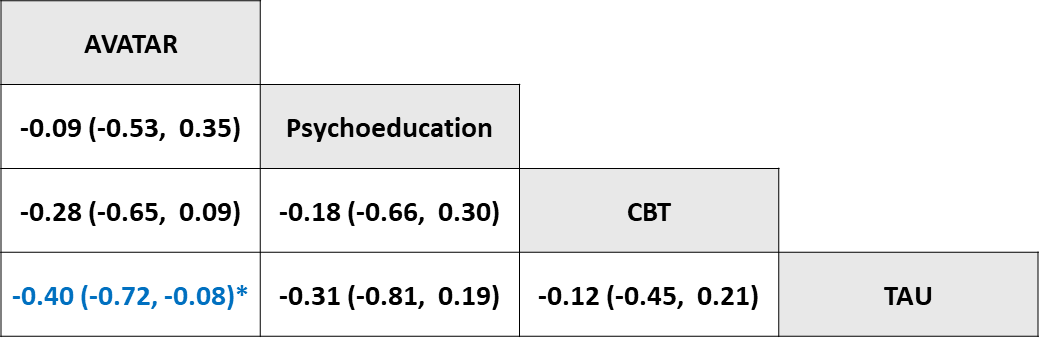 |

eFigure 15. Comparisons of different treatments for quality of life

| 1. Forest plot |
| --- |
| 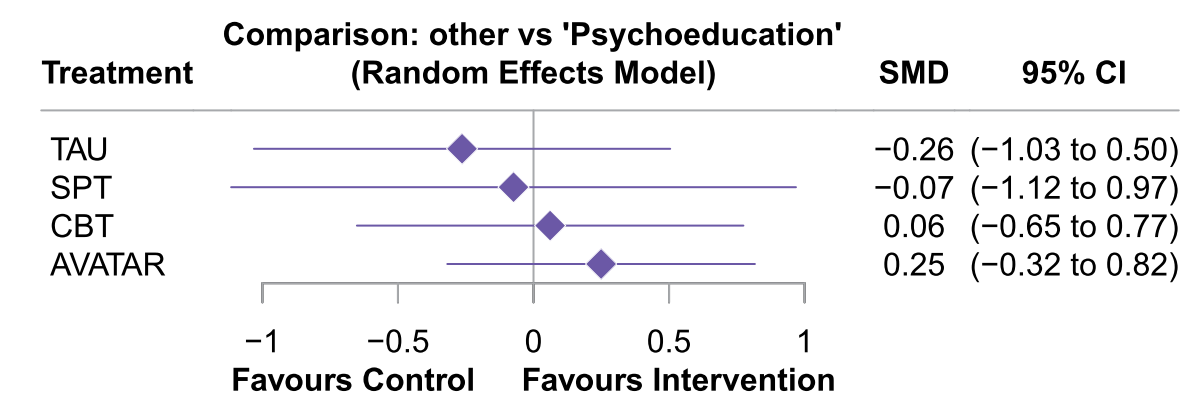 |
| 1. League table |
| 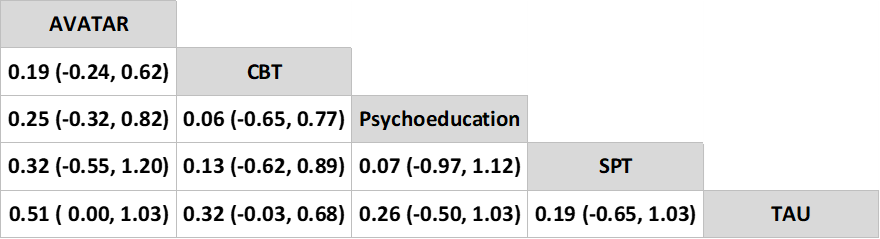 |

eFigure 16. Comparisons of different treatments for all-cause discontinuation

| 1. Forest plot |
| --- |
| 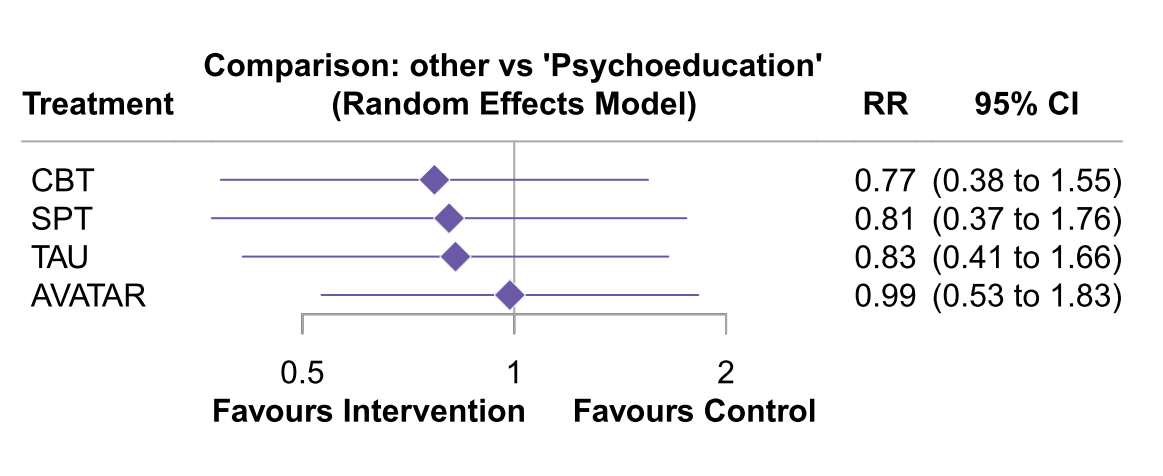 |
| 1. League table |
| 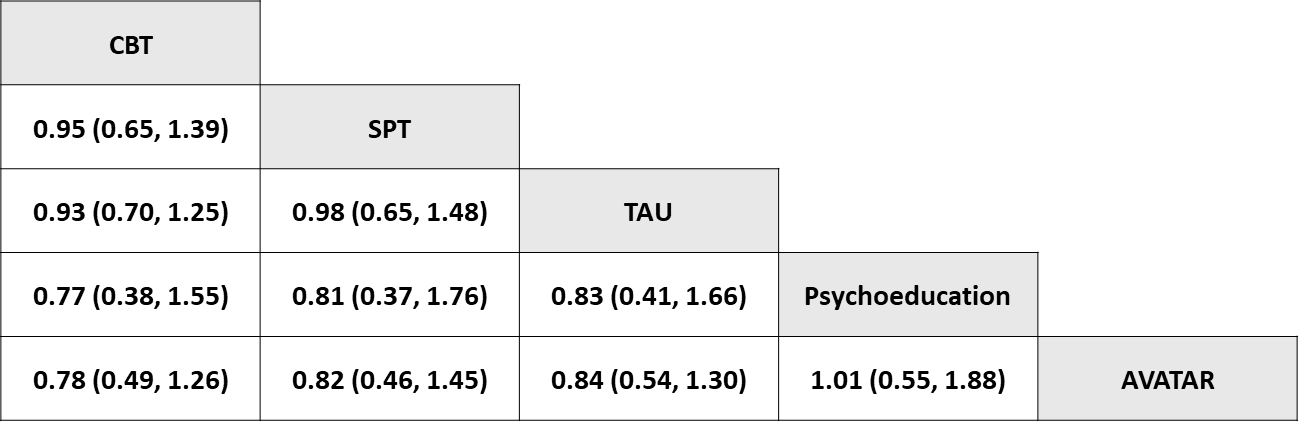 |

eFigure 17. Probability of being best treatment for severity of treatment-resistant auditory hallucinations


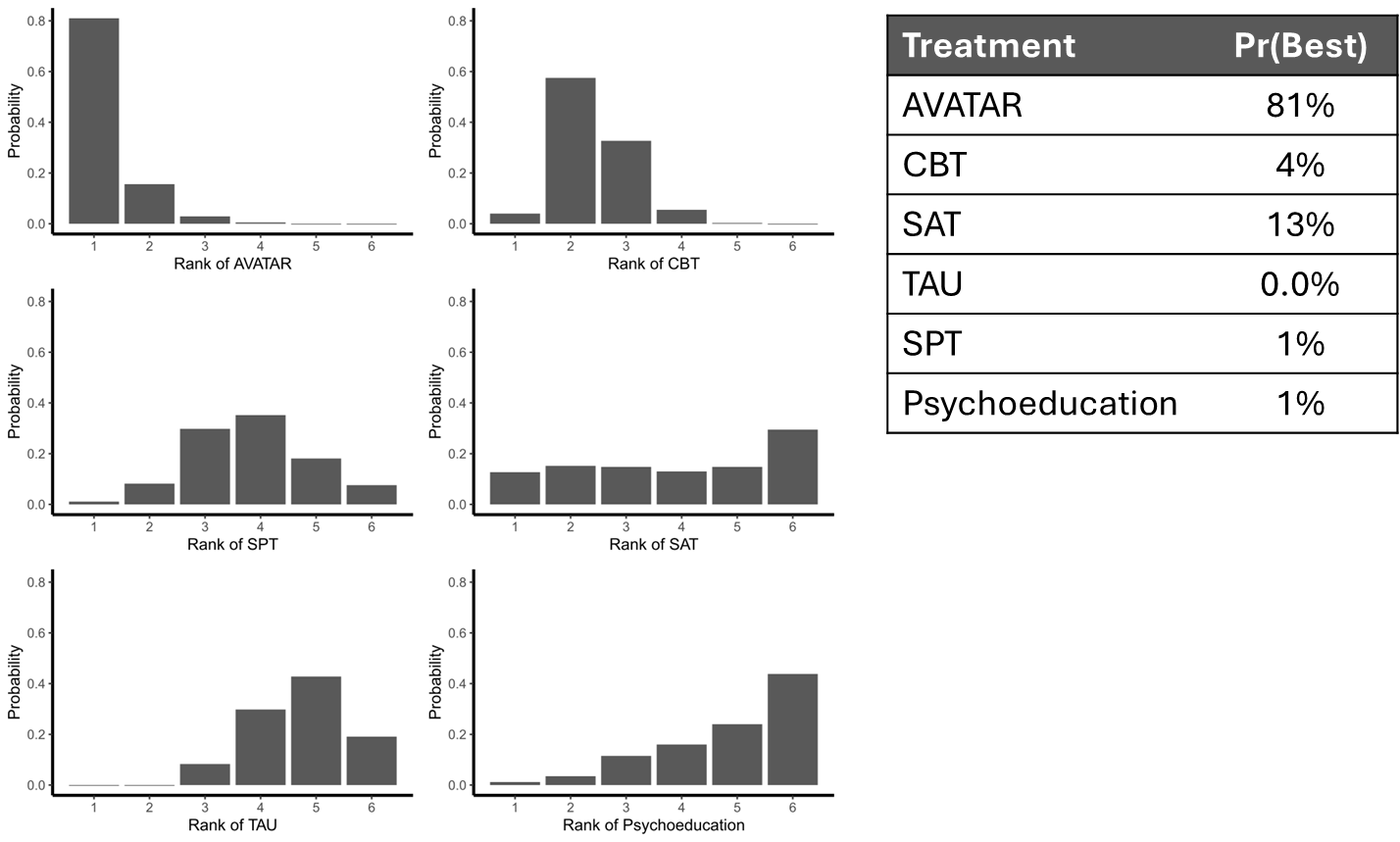


Abbreviations: AVATAR = audio visual assisted therapy aid for refractory auditory hallucinations; CBT = cognitive behavioral therapy; SAT = social activity therapy; SPT = supportive psychotherapy; TAU = treatment-as-usual

eFigure 18. Probability of being best treatment for long-term follow-up effects on treatment-resistant auditory hallucinations


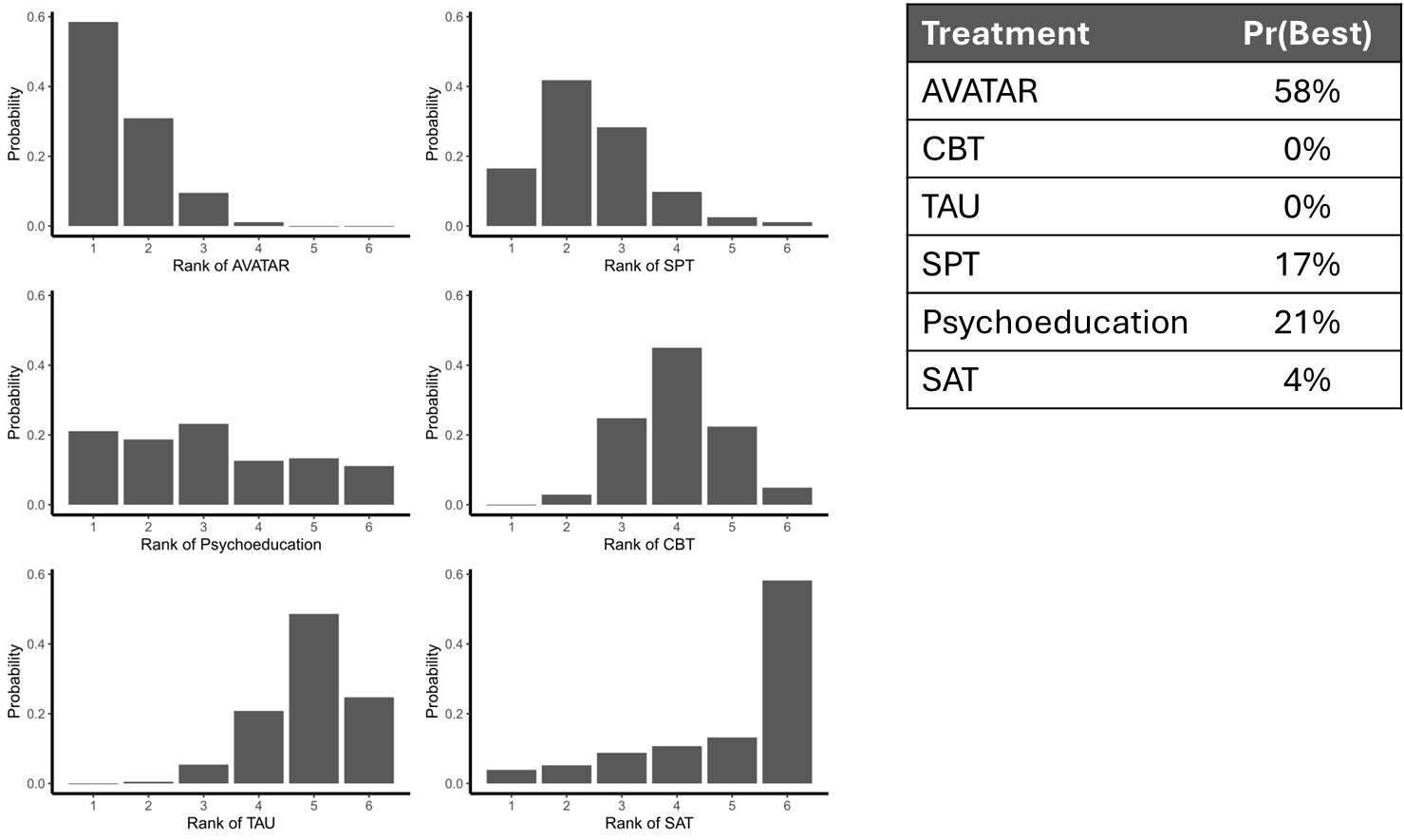


Abbreviations: AVATAR = audio visual assisted therapy aid for refractory auditory hallucinations; CBT = cognitive behavioral therapy; SAT = social activity therapy; SPT = supportive psychotherapy; TAU = treatment-as-usual

eFigure 19. Probability of being best treatment for overall psychotic symptoms measured by PANSS


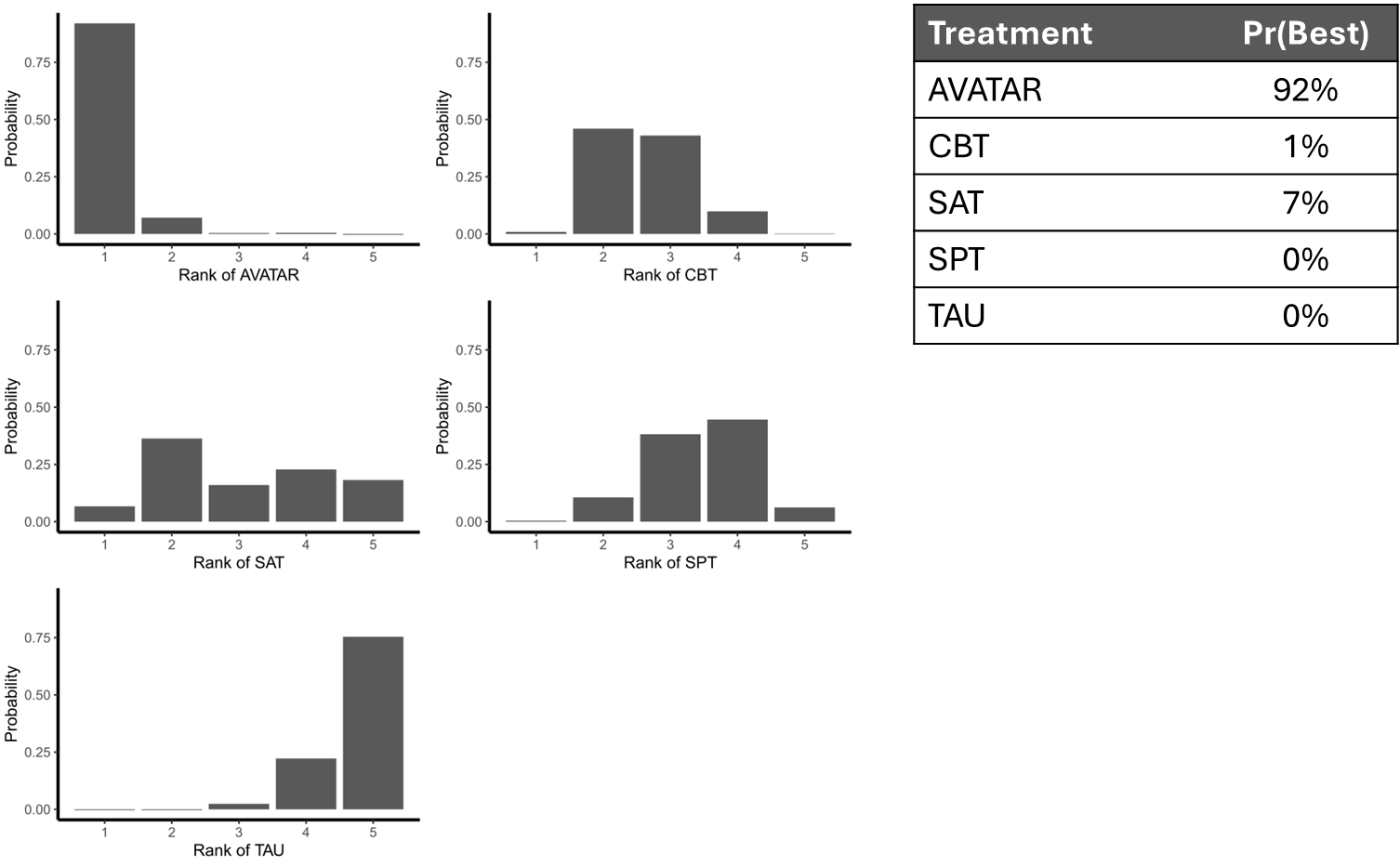


Abbreviations: AVATAR = audio visual assisted therapy aid for refractory auditory hallucinations; CBT = cognitive behavioral therapy; PANSS = Positive and Negative Syndrome Scale for Schizophrenia; SAT = social activity therapy; SPT = supportive psychotherapy; TAU = treatment-as-usual

eFigure 20. Probability of being best treatment for positive psychotic symptoms measured by PANSS


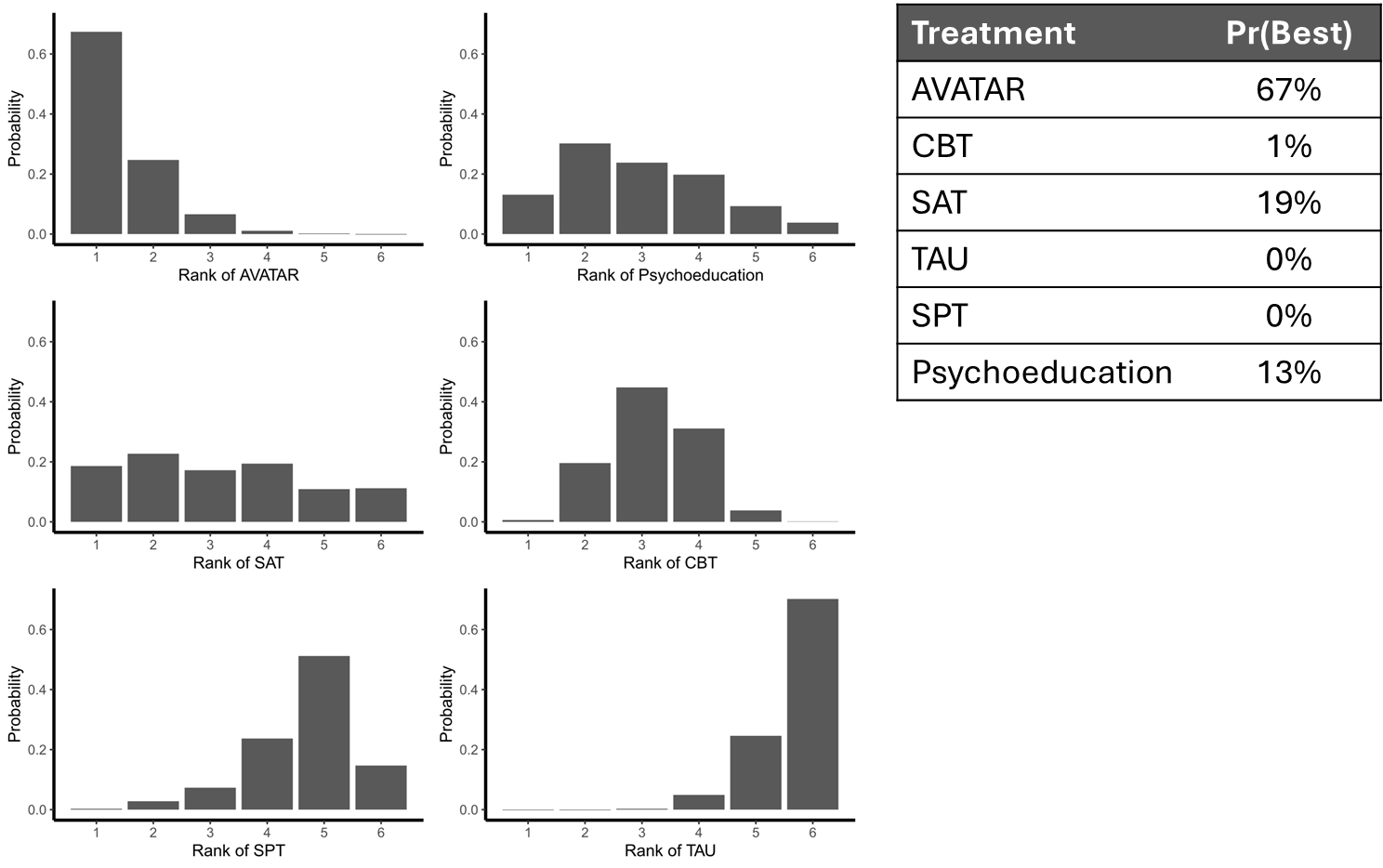


Abbreviations: AVATAR = audio visual assisted therapy aid for refractory auditory hallucinations; CBT = cognitive behavioral therapy; PANSS = Positive and Negative Syndrome Scale for Schizophrenia; SAT = social activity therapy; SPT = supportive psychotherapy; TAU = treatment-as-usua

eFigure 21. Probability of being best treatment for negative psychotic symptoms measured by PANSS


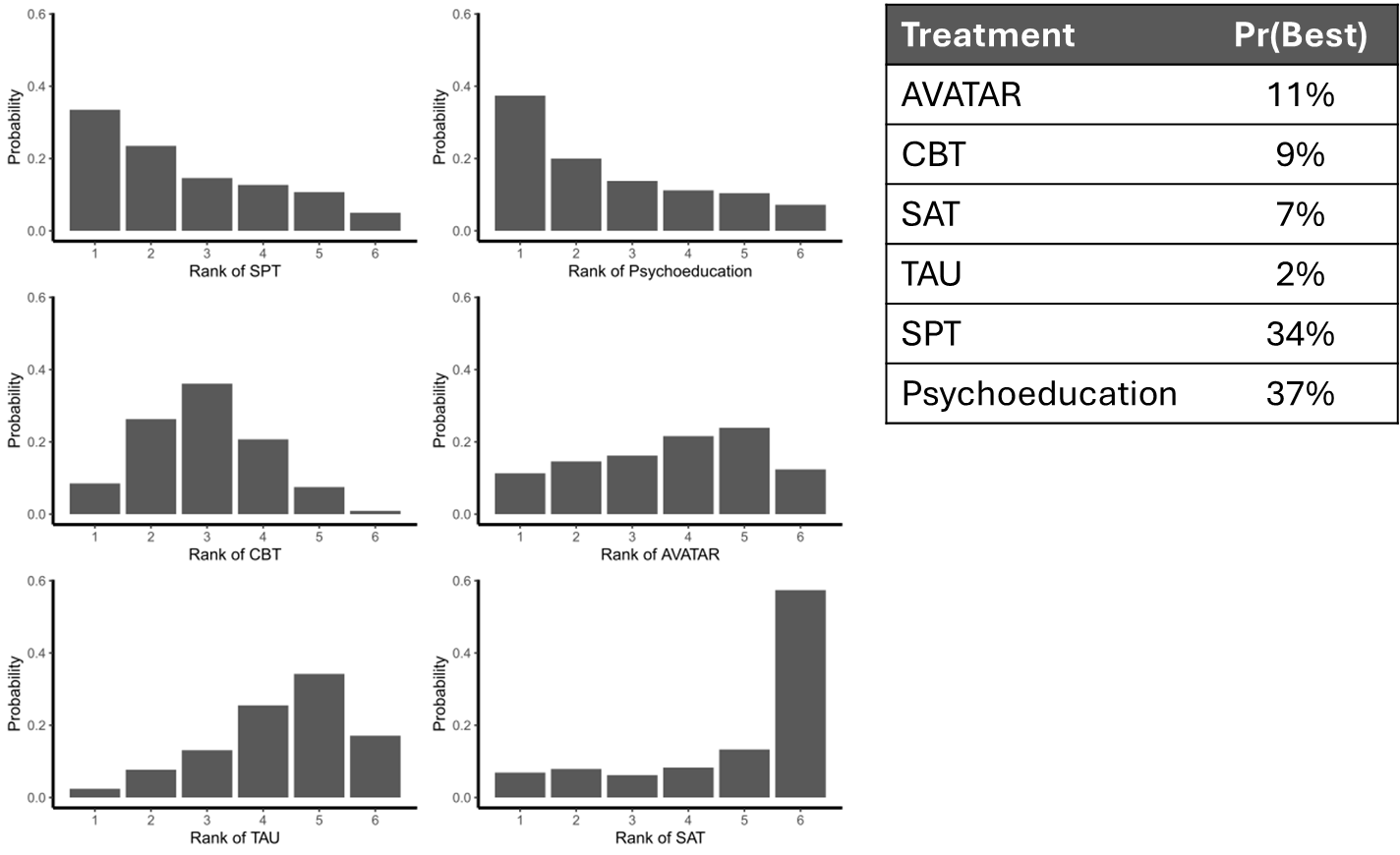


Abbreviations: AVATAR = audio visual assisted therapy aid for refractory auditory hallucinations; CBT = cognitive behavioral therapy; PANSS = Positive and Negative Syndrome Scale for Schizophrenia; SAT = social activity therapy; SPT = supportive psychotherapy; TAU = treatment-as-usua

eFigure 22. Probability of being best treatment for depressive symptoms


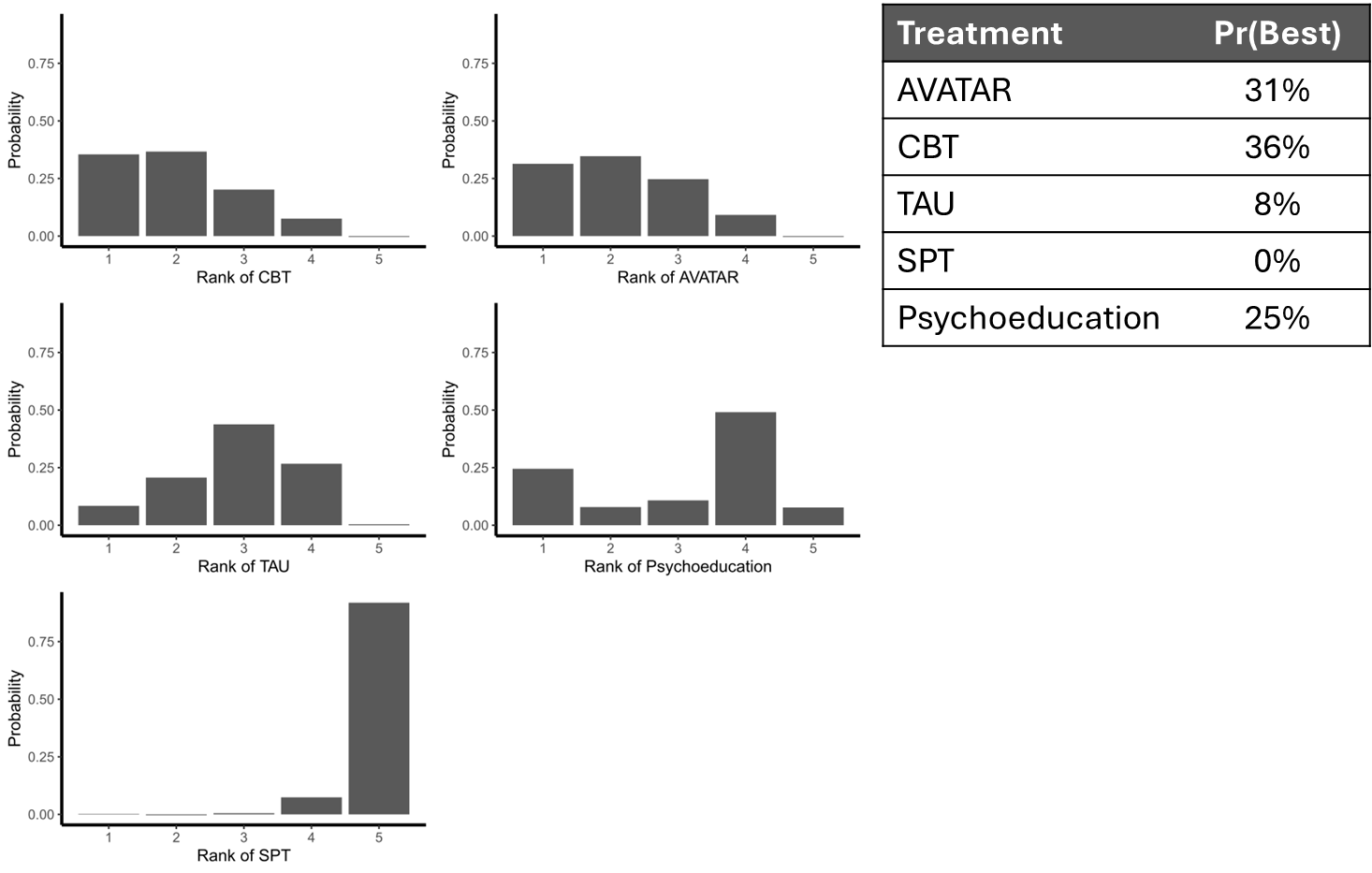


Abbreviations: AVATAR = audio visual assisted therapy aid for refractory auditory hallucinations; CBT = cognitive behavioral therapy; SAT = social activity therapy; SPT = supportive psychotherapy; TAU = treatment-as-usual

eFigure 23. Probability of being best treatment for anxiety symptoms


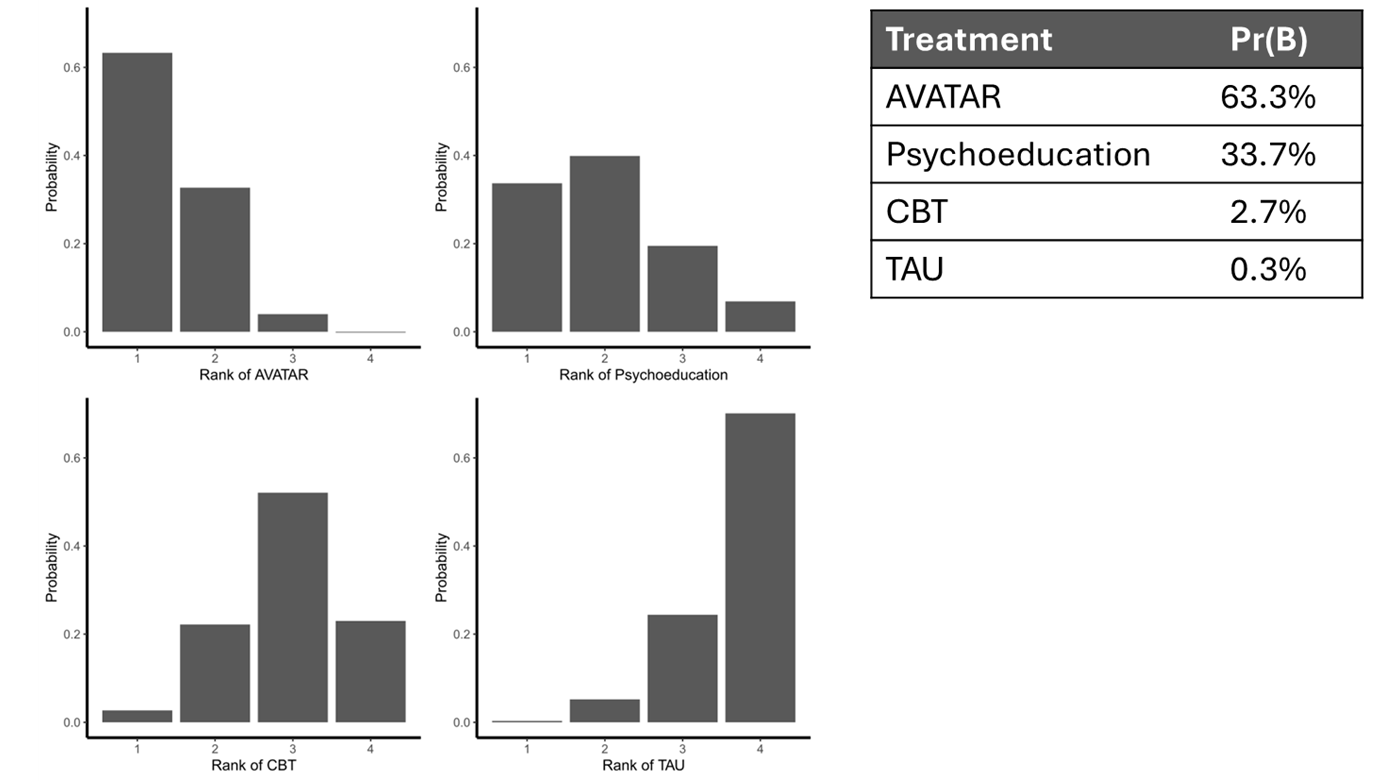


Abbreviations: AVATAR = audio visual assisted therapy aid for refractory auditory hallucinations; CBT = cognitive behavioral therapy; SAT = social activity therapy; TAU = treatment-as-usual

eFigure 24. Probability of being best treatment for quality of life


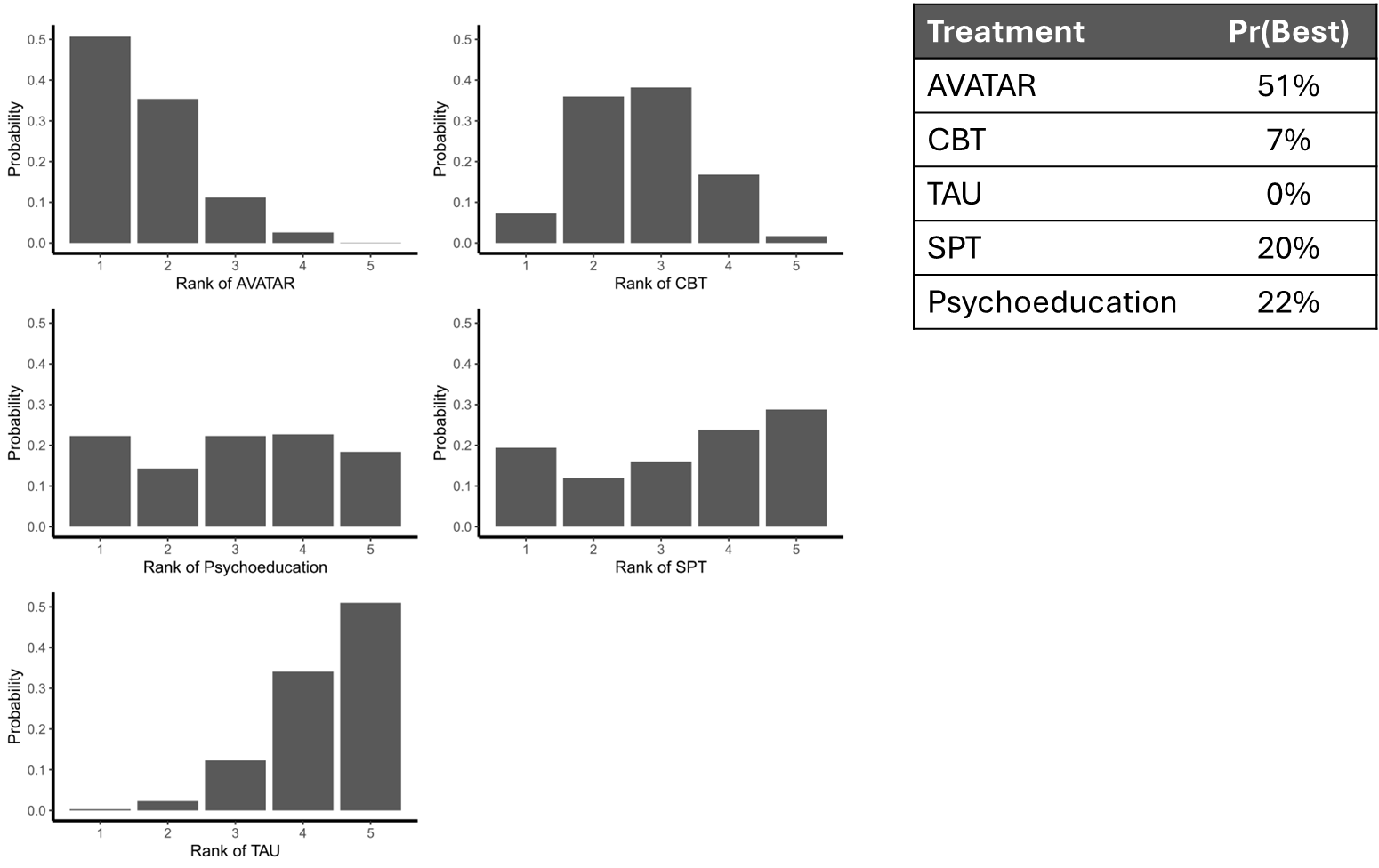


Abbreviations: AVATAR = audio visual assisted therapy aid for refractory auditory hallucinations; CBT = cognitive behavioral therapy; SAT = social activity therapy; TAU = treatment-as-usual

eFigure 25. Probability of being best treatment for all-cause discontinuation


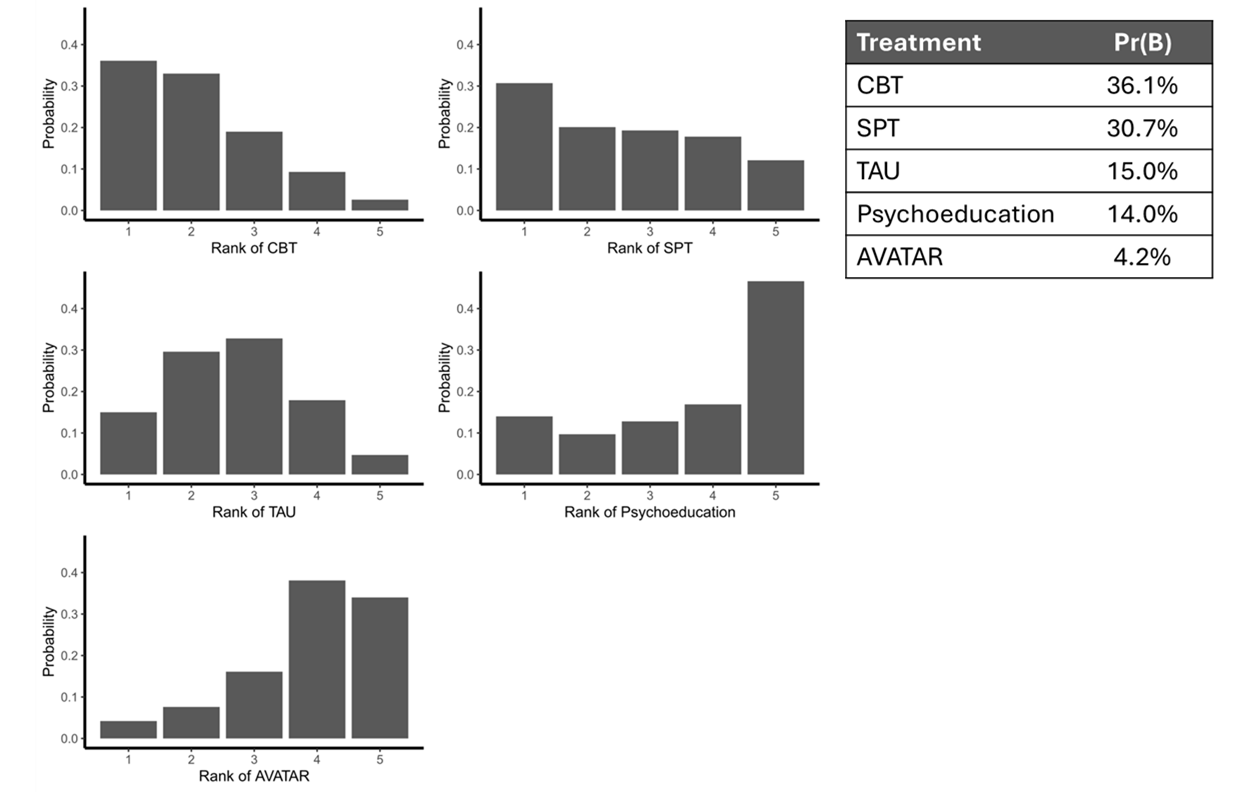


Abbreviations: AVATAR = audio visual assisted therapy aid for refractory auditory hallucinations; CBT = cognitive behavioral therapy; SPT = supportive psychotherapy; TAU = treatment-as-usual

eFigure 26. Funnel plot of severity of auditory hallucinations: order by treatment-as-usual


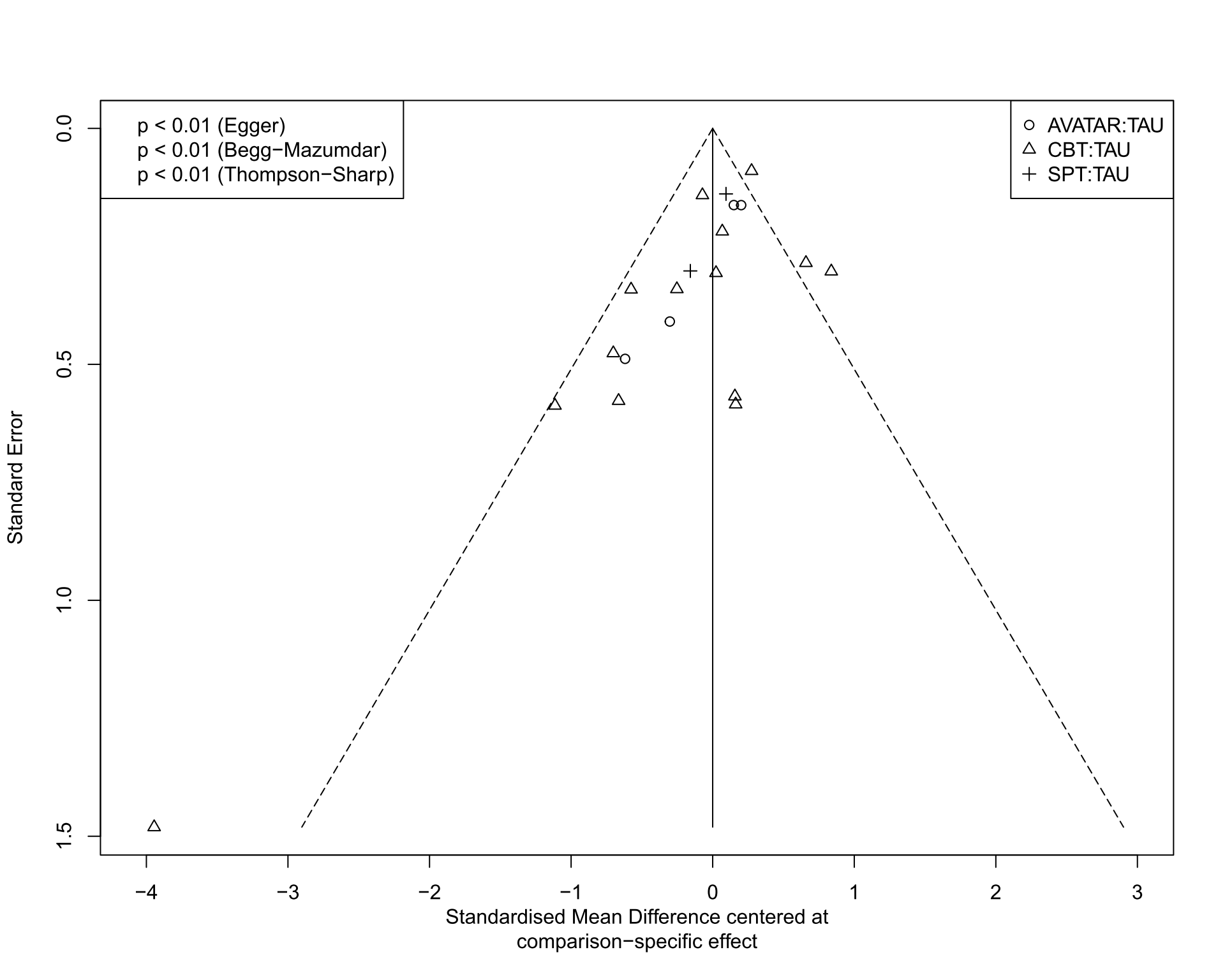


eFigure 27. Funnel plot of long-term follow-up effects on treatment-resistant auditory hallucinations: order by treatment-as-usual


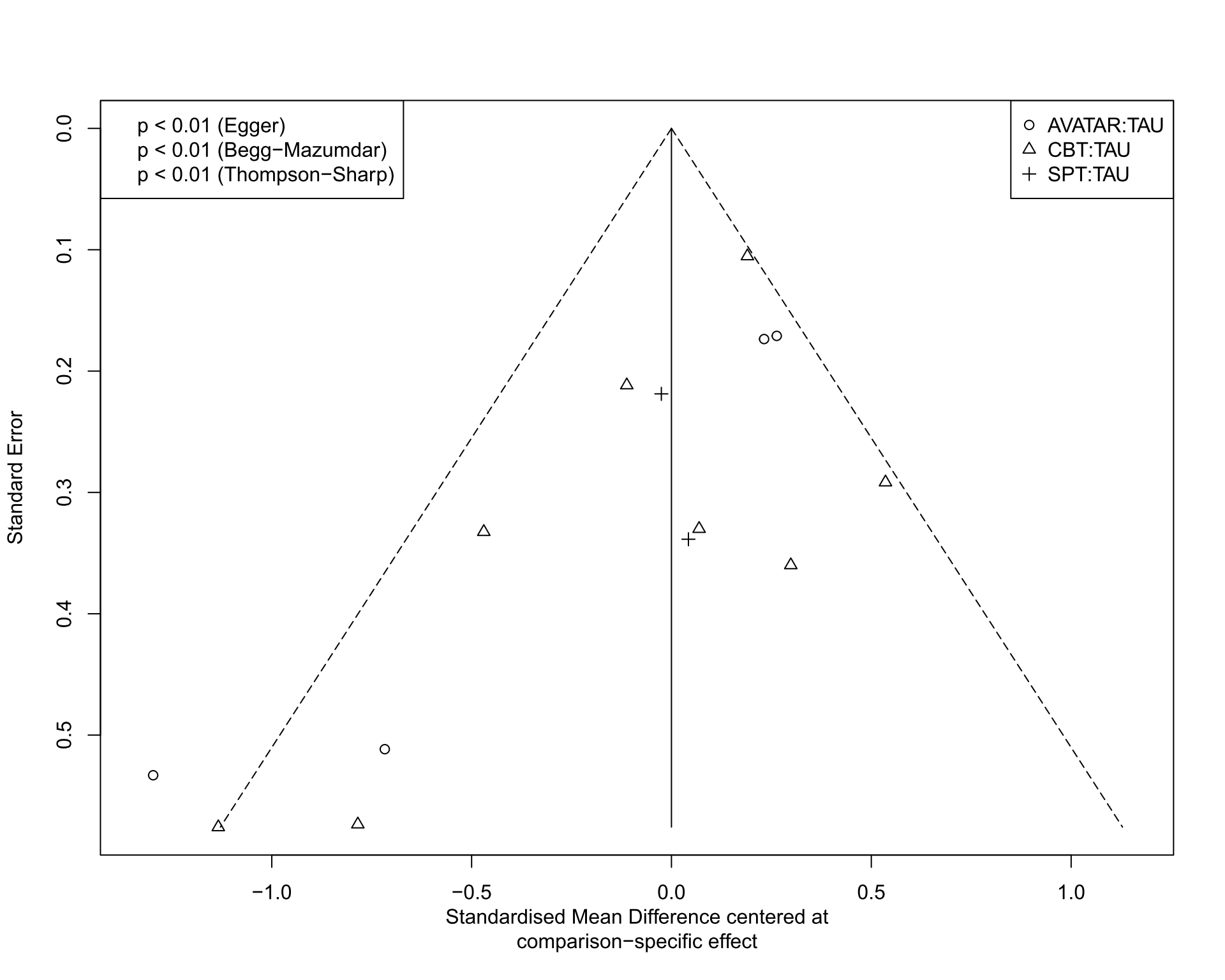


eFigure 28. Funnel plot of overall psychotic symptoms: order by treatment-as-usual


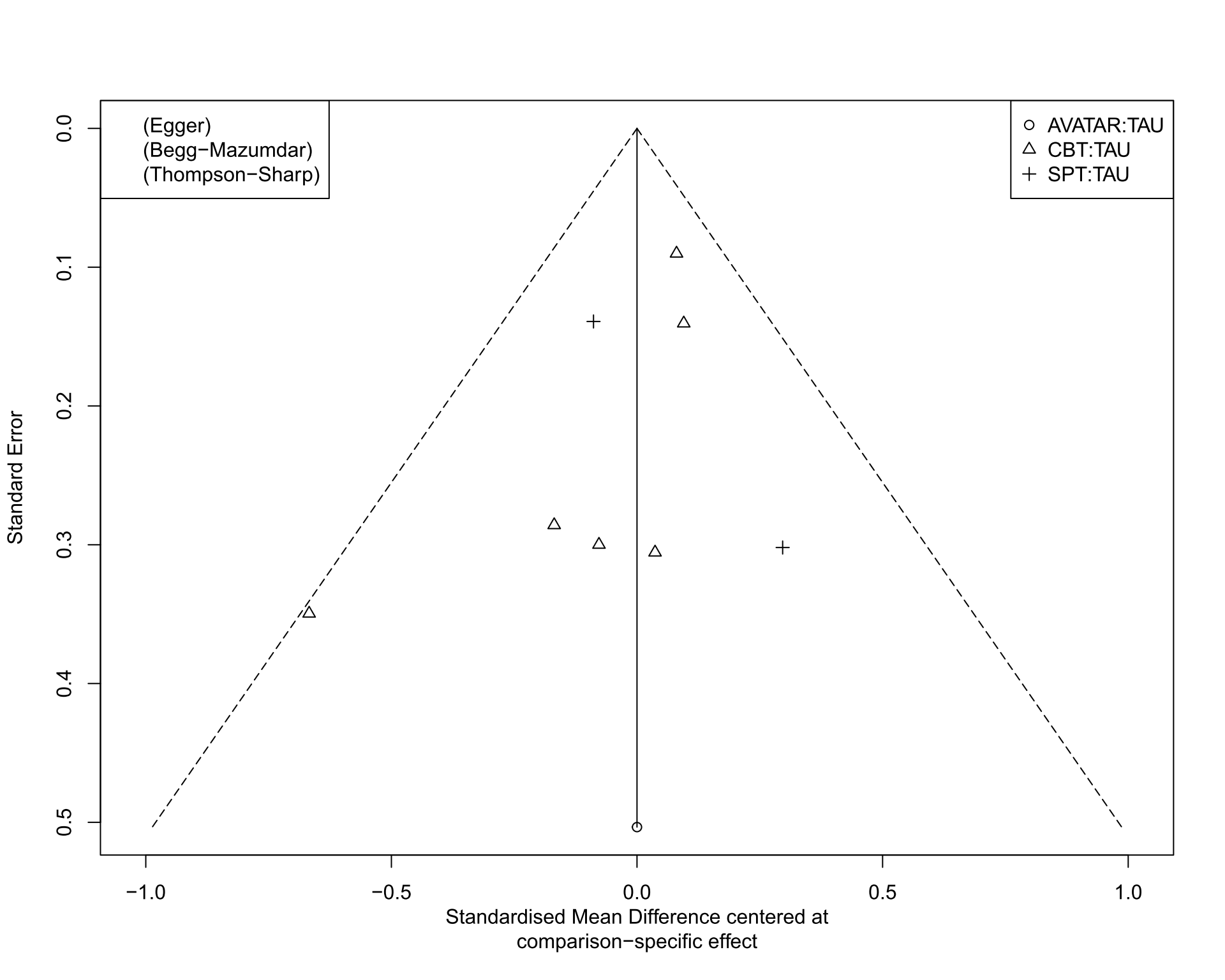


eFigure 29. Funnel plot of positive psychotic symptoms: order by treatment-as-usual


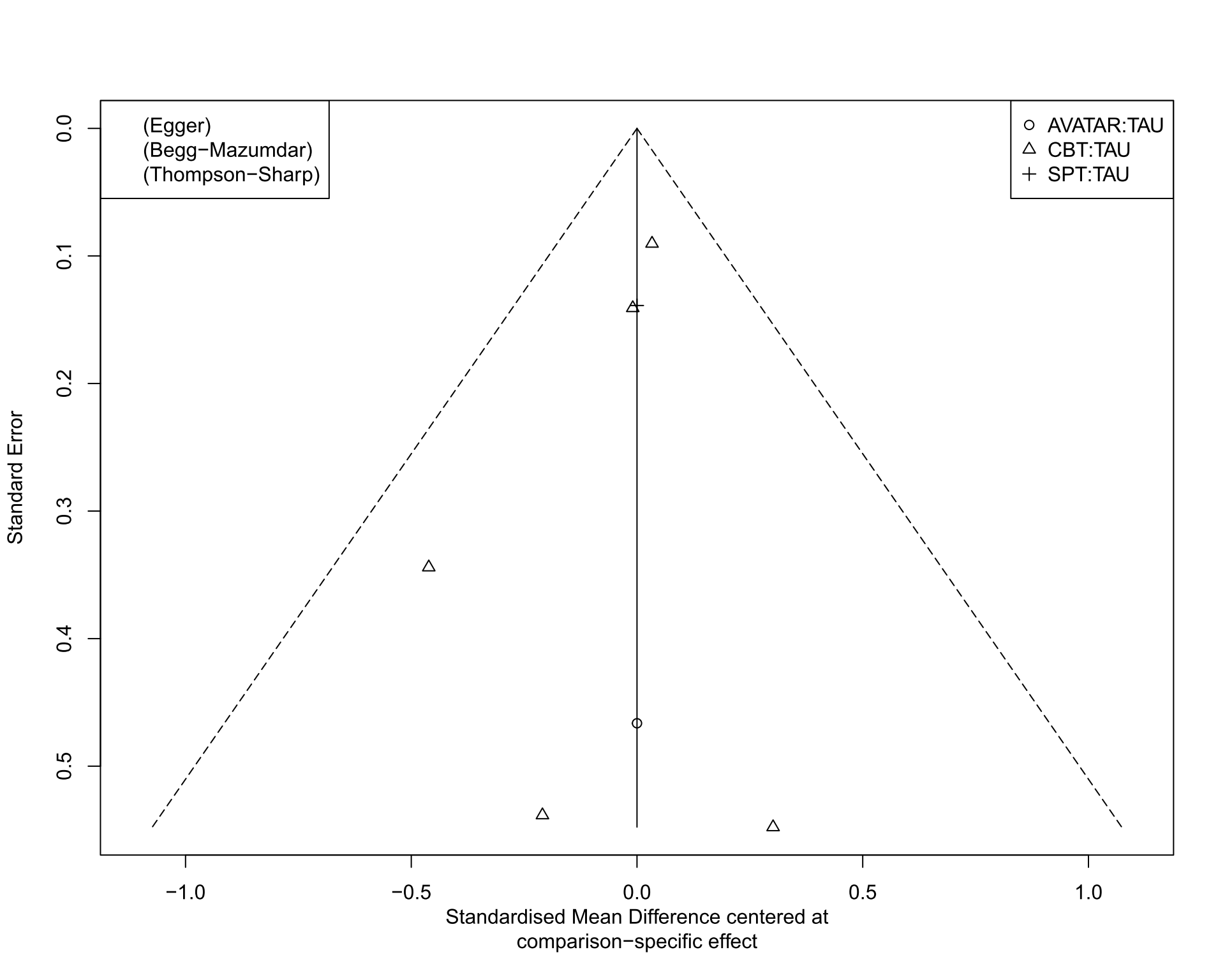


eFigure 30. Funnel plot of negative psychotic symptoms: order by treatment-as-usual


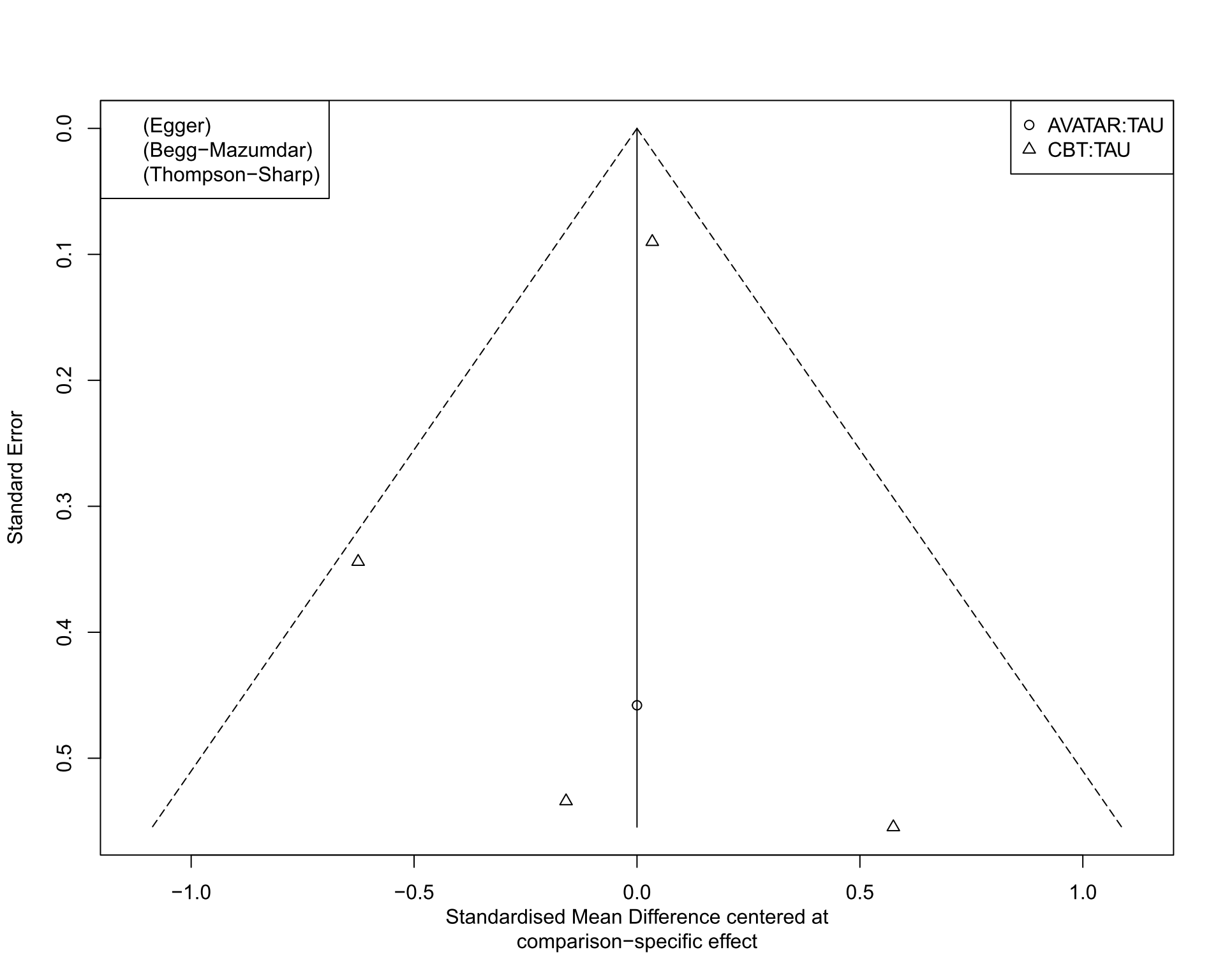


eFigure 31. Funnel plot of depressive symptoms: order by treatment-as-usual


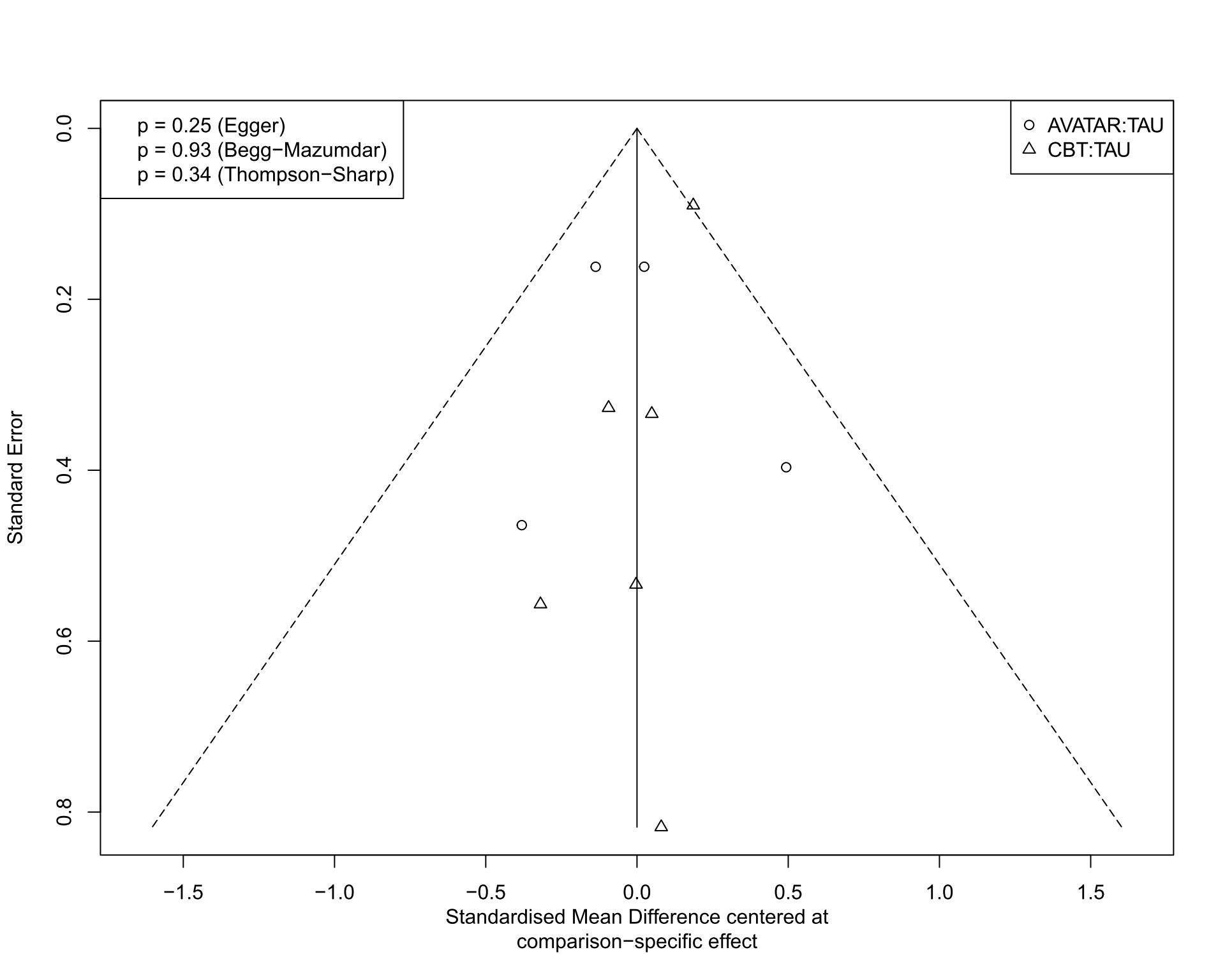


eFigure 32. Funnel plot of anxiety symptoms: order by treatment-as-usual


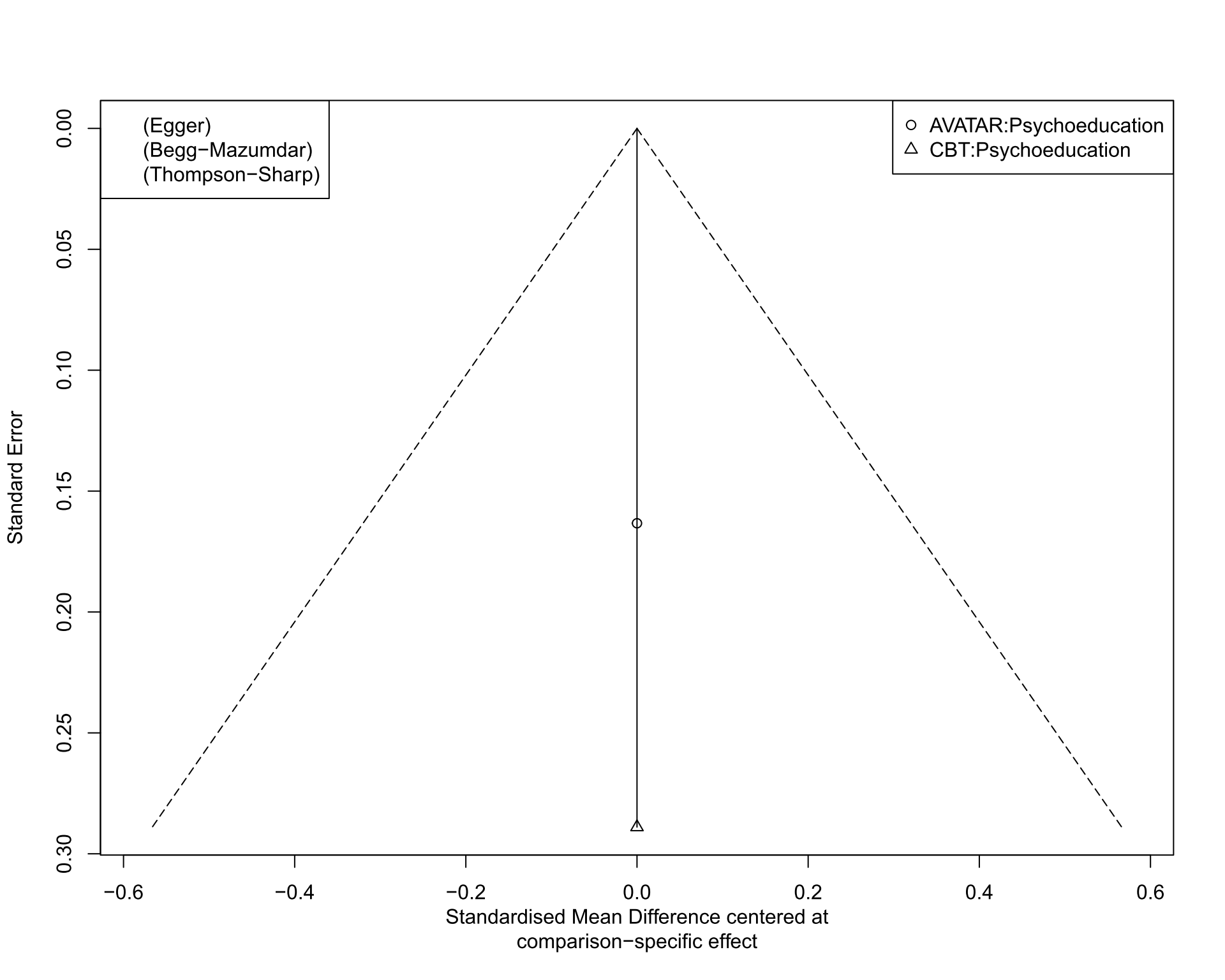


eFigure 33. Funnel plot of quality of life: order by treatment-as-usual


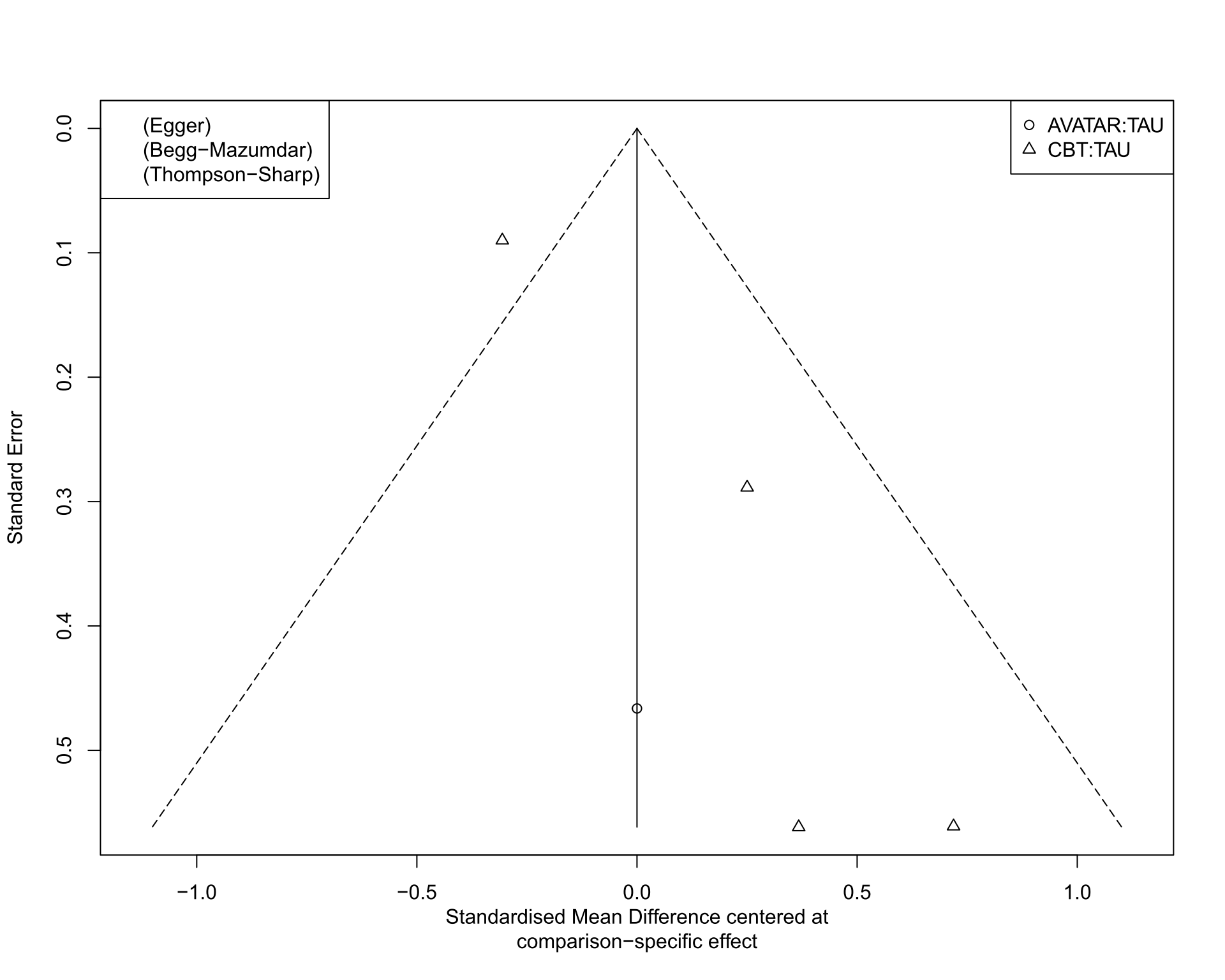


eFigure 34. Funnel plot of all-cause discontinuation: order by treatment-as-usual
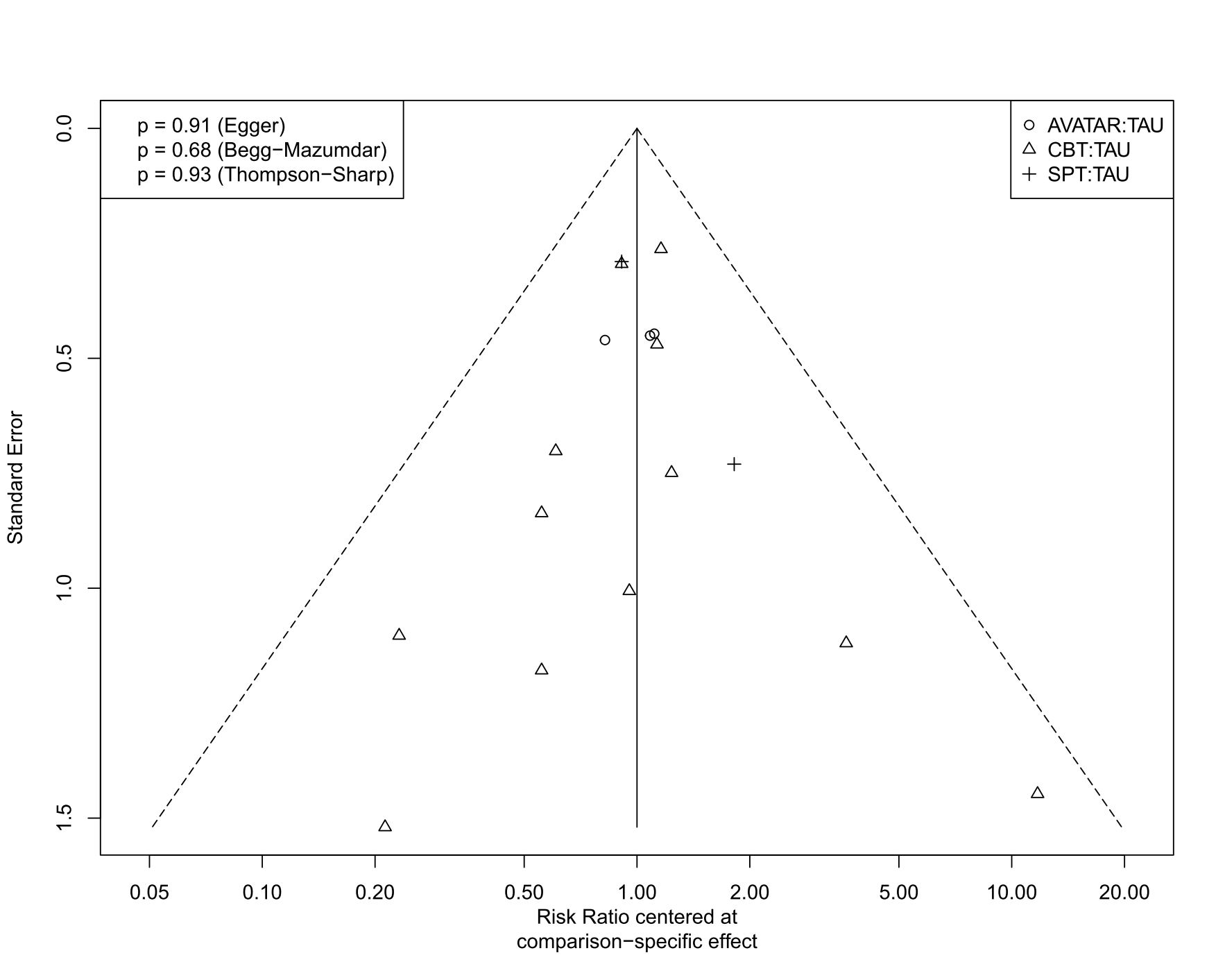


eFigure 35. Visual inspection of transitivity assumption for potential effect modifier: age


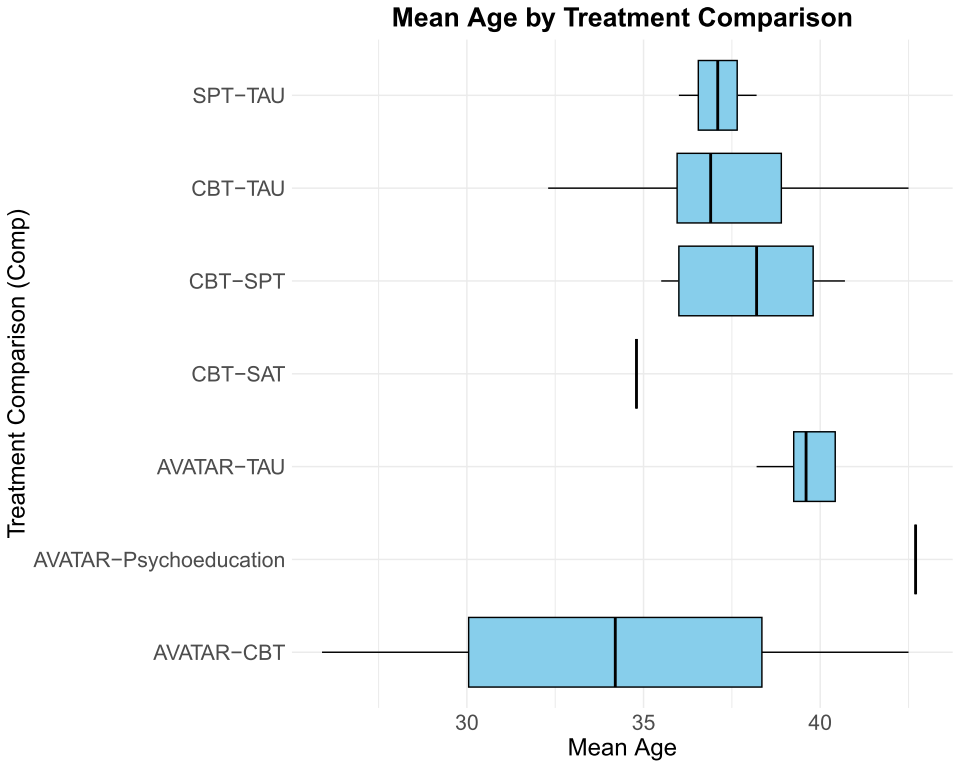


eFigure 36. Visual inspection of transitivity assumption for potential effect modifier: female proportion


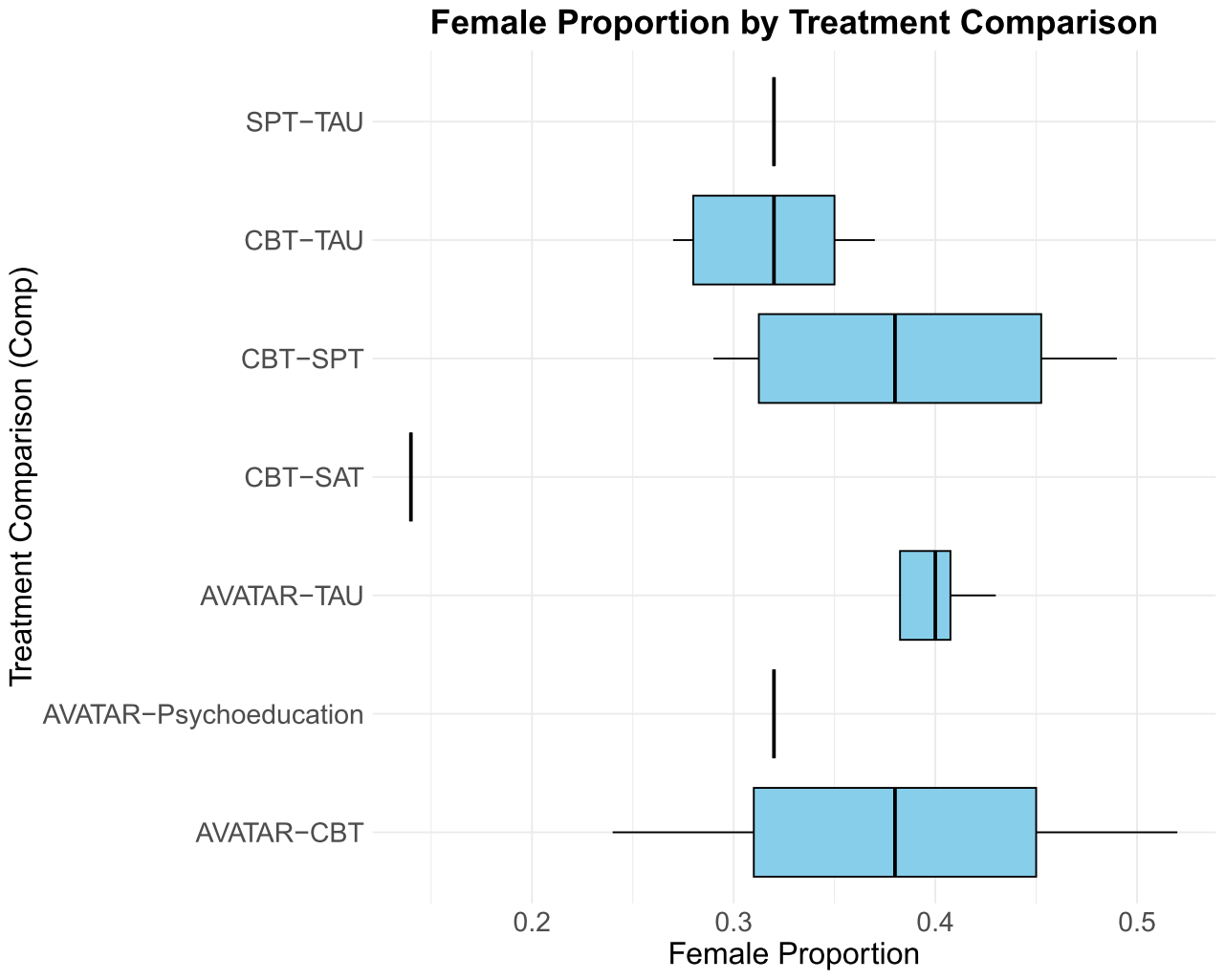


eFigure 37. Visual inspection of transitivity assumption for potential effect modifier: study duration


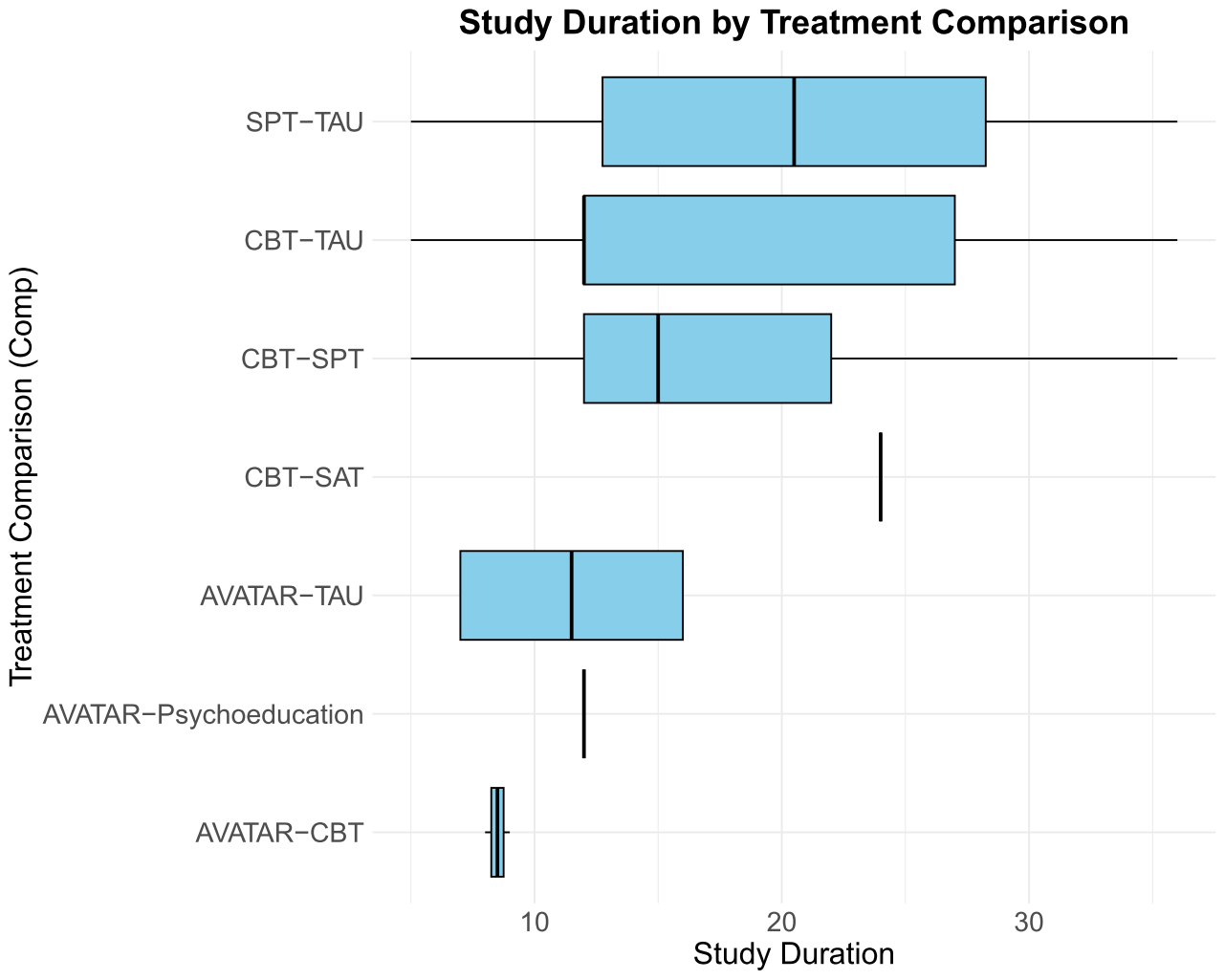


eFigure 38. Visual inspection of transitivity assumption for potential effect modifier: resistance level


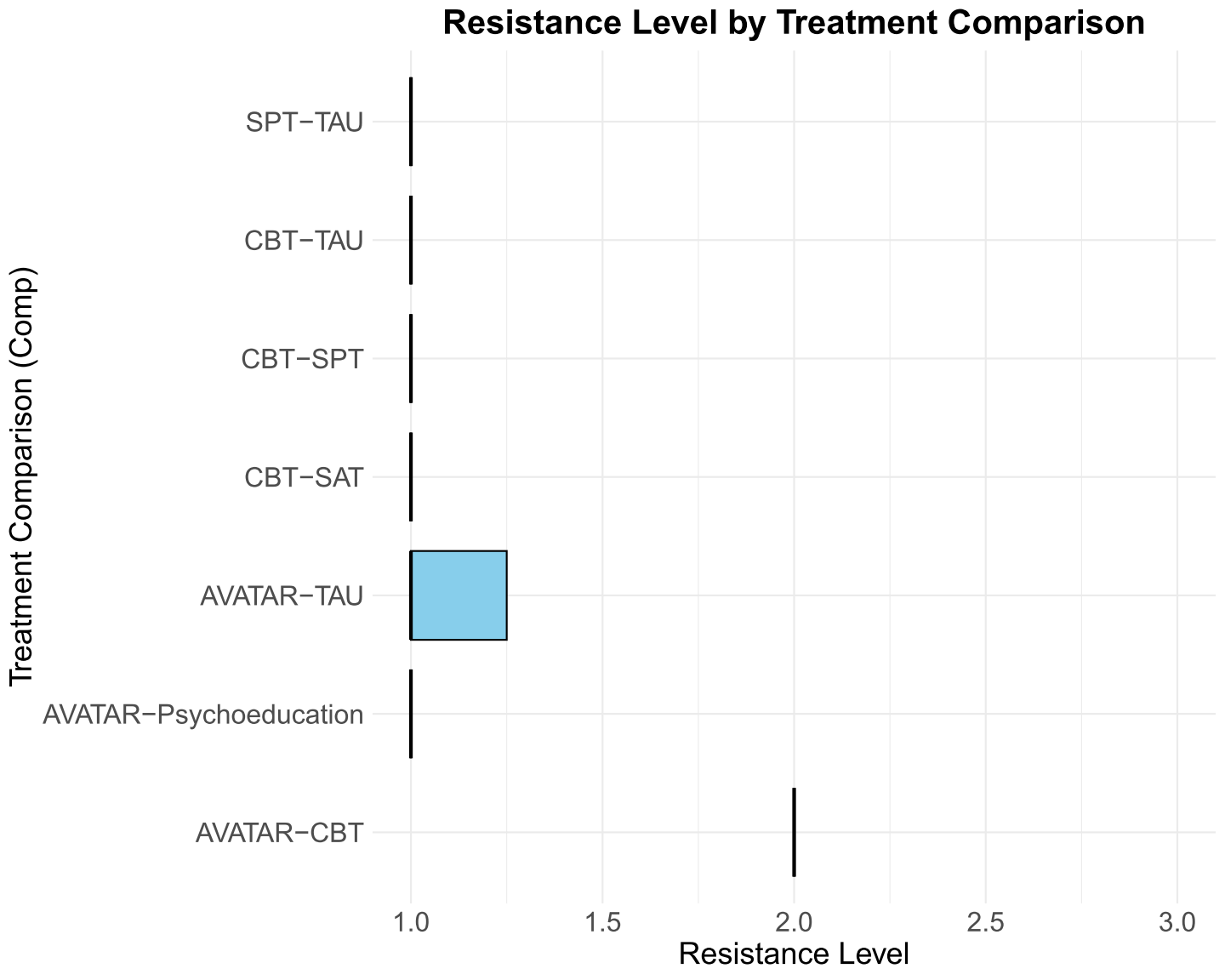


eFigure 39. Network meta-regression for potential effect modifier: age


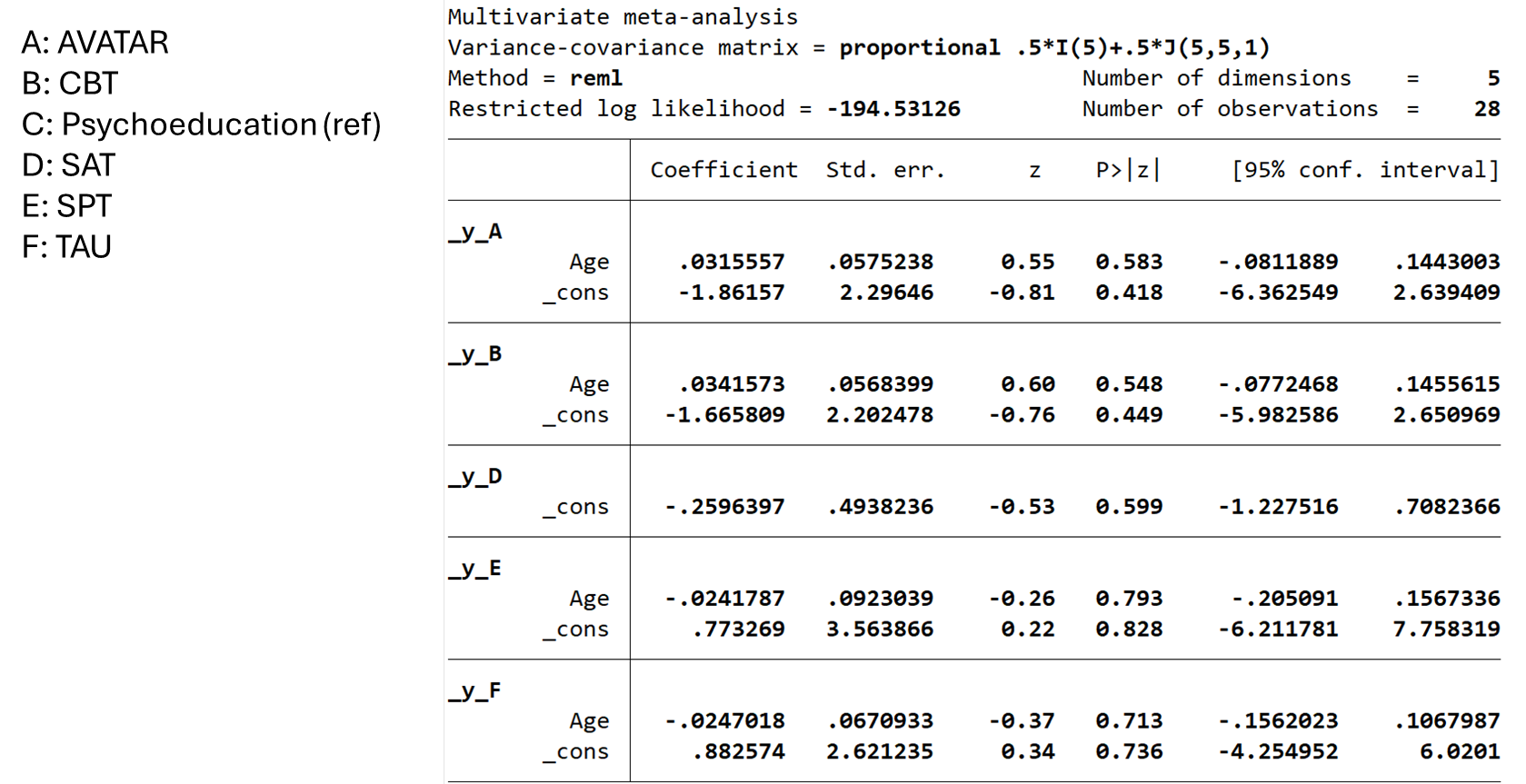


eFigure 40. Network meta-regression for potential effect modifier: female proportion


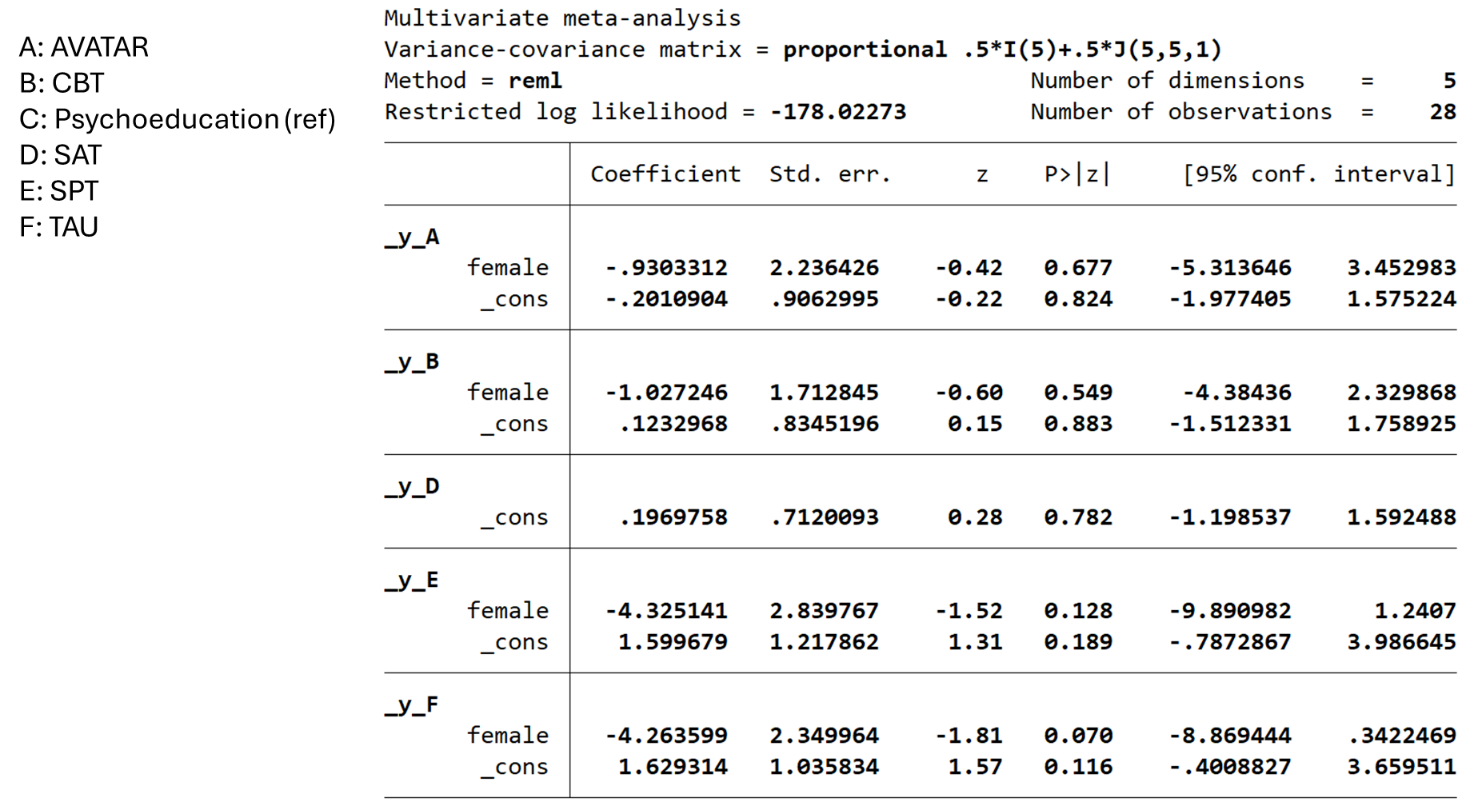


eFigure 41. Network meta-regression for potential effect modifier: study duration


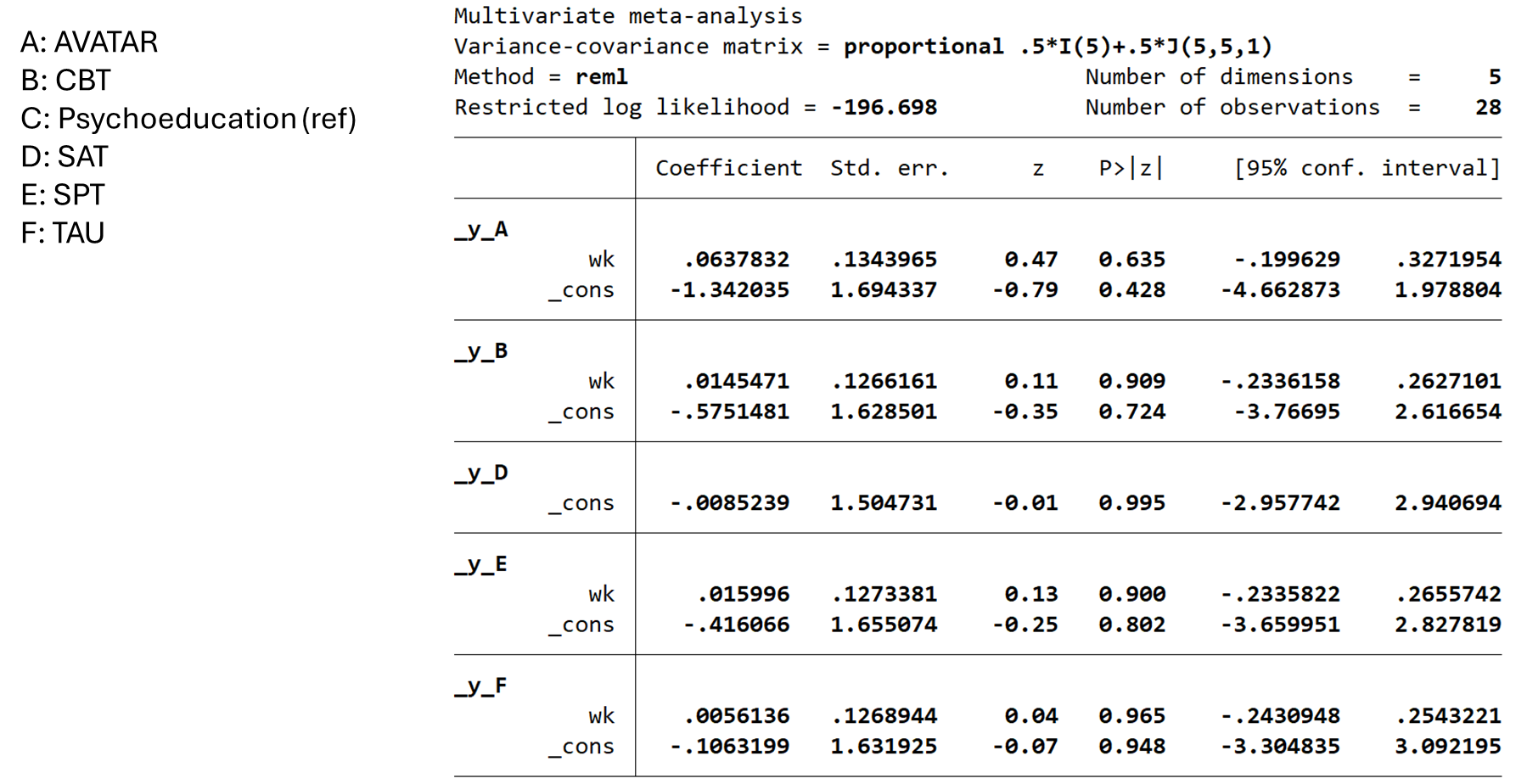


eFigure 42. Network meta-regression for potential effect modifier: resistance level


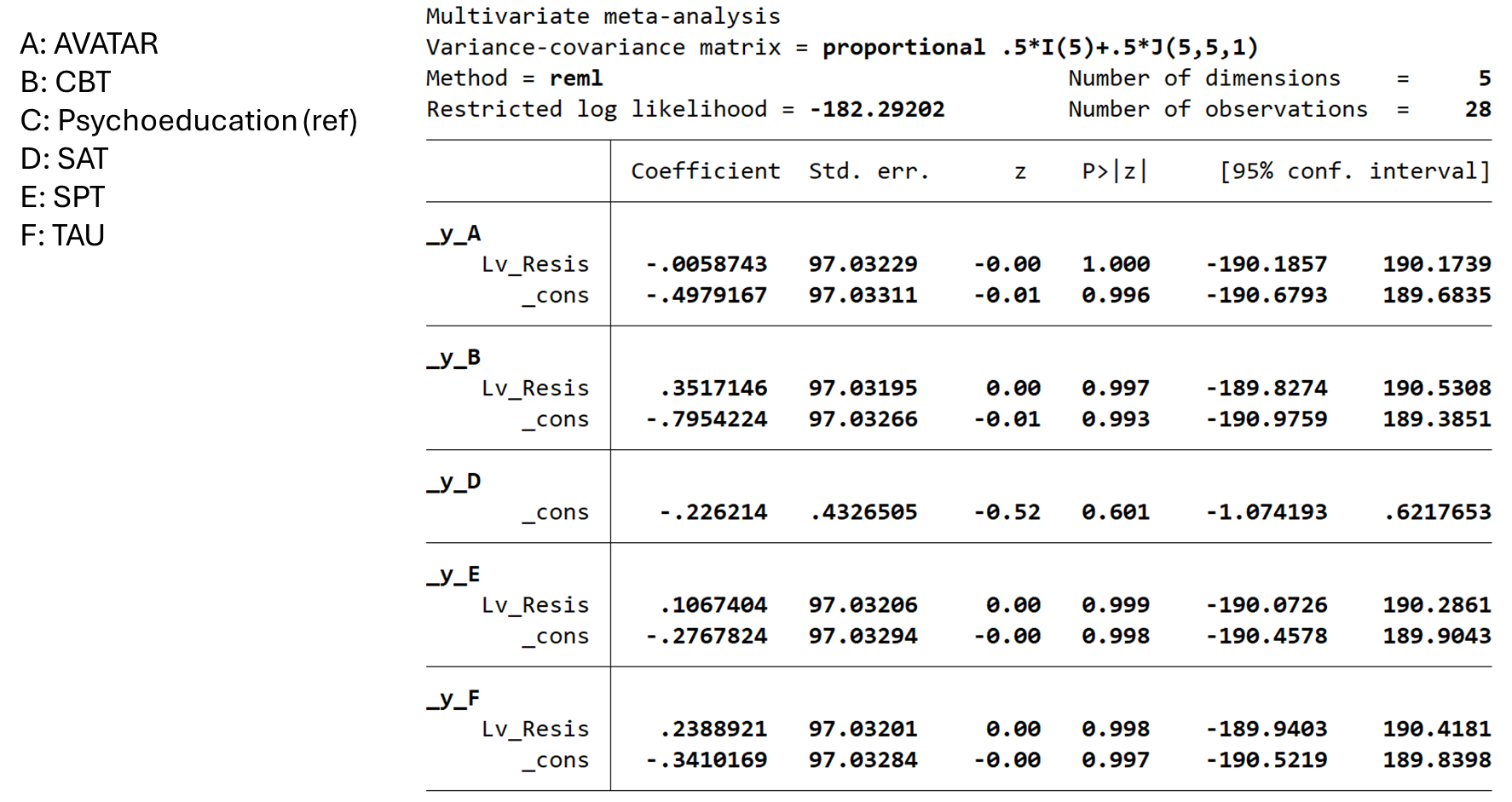


eFigure 43. The evaluation of risk of bias due to missing evidence for the primary outcome


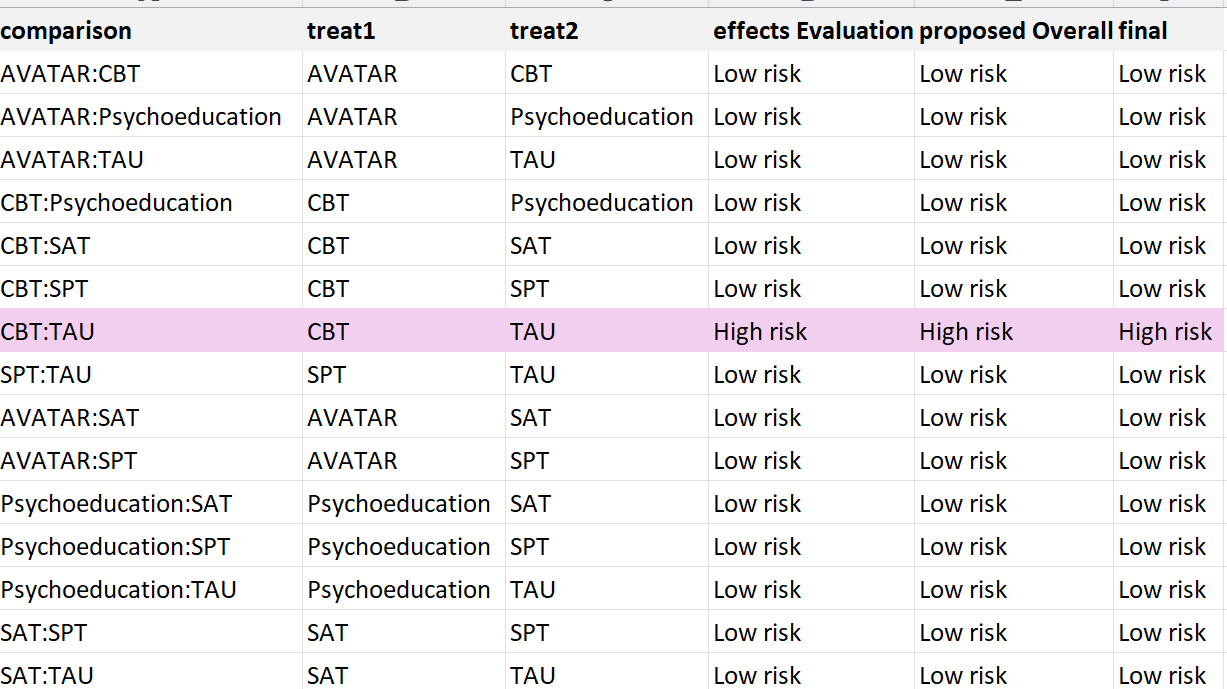


eFigure 44. Sensitivity analysis of excluding group therapy


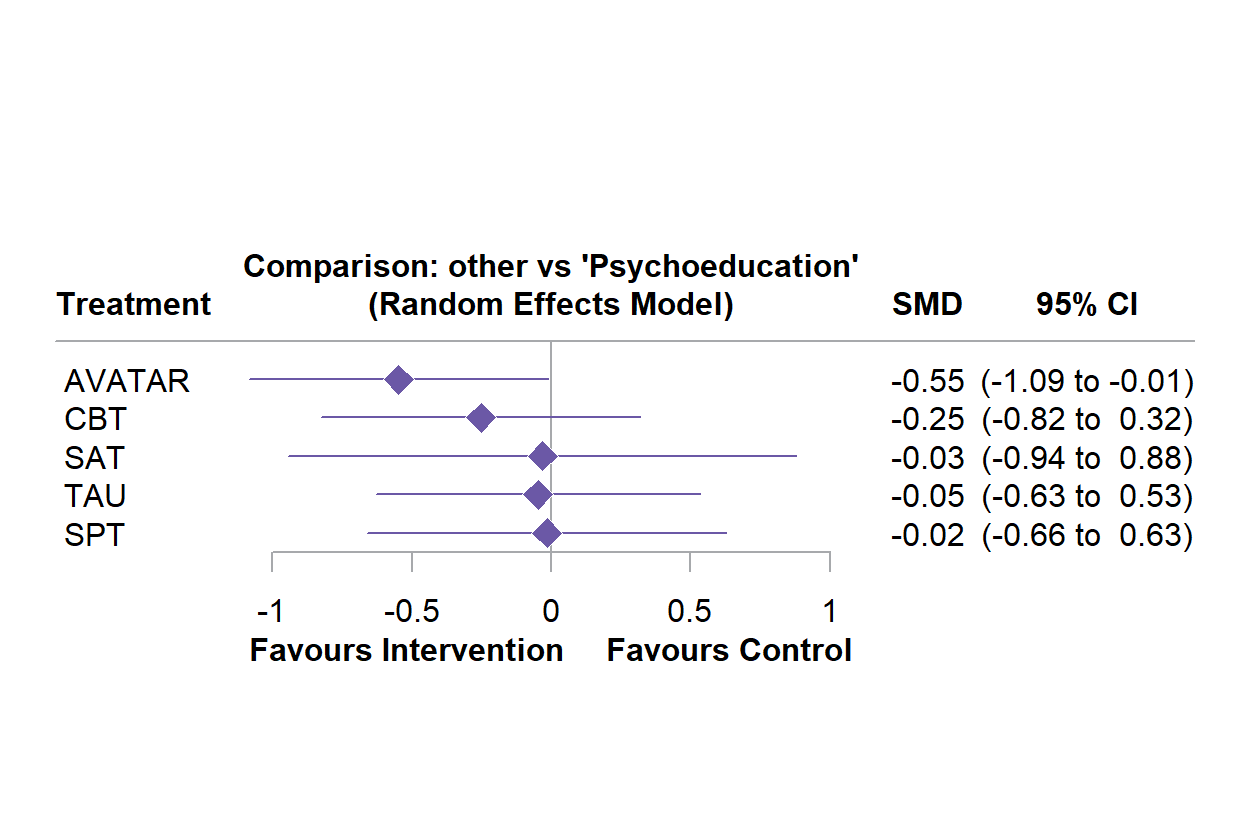


eFigure 45. Sensitiviy analysis of the studies with acutal 3-month follow-up data on the primary outcome


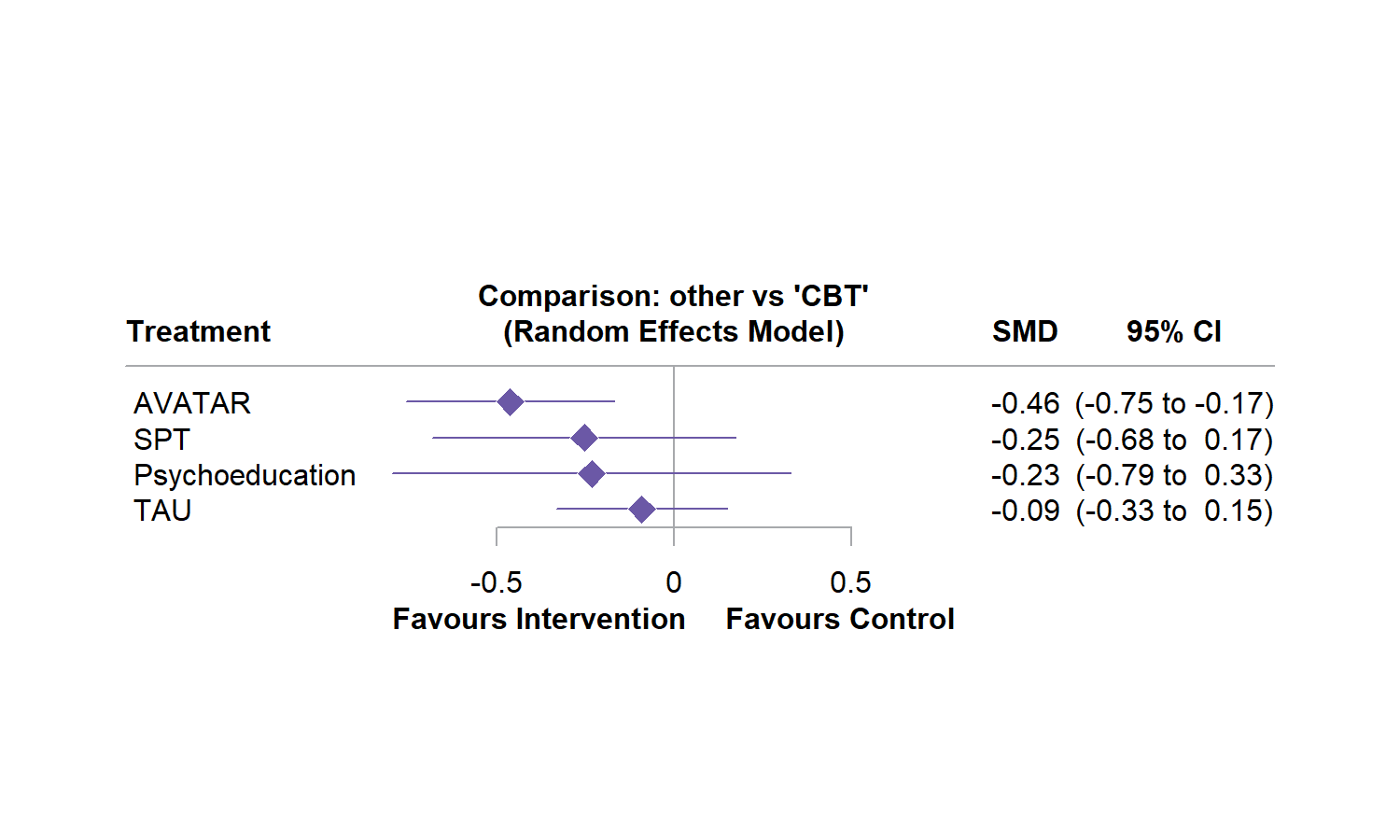


eTable 1. Description of included studies

| Study/ Country  / Study design | Interventions | n/ n of male/ mean age (year) | Total sessions/ Treatment duration | Diagnoses of participants | Diagnostic criteria | Baseline mean overall PANSS (SD) | Treatment-resistant definition level/  Group or Individual | % of participants treated with clozapine |
| --- | --- | --- | --- | --- | --- | --- | --- | --- |
| Wahaas 1997/ UK// OL | CBT | 3/ NA/ 31.3 | 24/ 9 weeks | Schizophrenia (100%) | ICD-10 | NA | 1/ Individual | NA |
|  | TAU | 3/ NA/ 34.0 |  |  |  |  |  |  |
| Handdock 1998/ UK/ assessor-blinded | CBT-focus | 14/ 8/ 36.0 | 18-20/ 18-20 weeks | Schizophrenia (100%) | DSM-III | NA | 1/ Individual | NA |
|  | CBT-distract | 11/ 6/ 46.0 |  |  |  |  |  |  |
|  | TAU | 8/ NA/ NA |  |  |  |  |  |  |
| Lewis 2002/ Canada/ assessor-blinded | CBT | 101/NA/ NA | Mean 16/ 5 weeks | Schizophrenia (39.8%)  Schizoaffective disorder (12.6%)  Schizophreniform (35.3%)  Delusional disorder (8.1%)  Psychosis NOS (4.2%) | DSM-IV | 87.47 (17.6) | 1/ Individual | NA |
|  | SPT | 106/NA/ NA |  |  |  | 89.22 (17.5) |  |  |
|  | TAU | 102/ NA/ NA |  |  |  | 87.01 (16.8) |  |  |
| Durham 2003/ UK/ assessor-blinded | CBT | 22/ 15/ 36.0 | 20/ 9 months | Schizophrenia (89.4%)  Schizoaffective disorder (7.6%)  Delusional disorder (3.0%) | DSM-IV, ICD-10 | 101.2 (14.7) | 1/ Individual | NA |
|  | SPT | 23/ 15/ 37.0 |  |  |  | 95 (17.7) |  |  |
|  | TAU | 21/ 15/ 36.0 |  |  |  | 92.4 (17.5) |  |  |
| Cather 2005/ UK/ assessor-blinded | CBT | 15/ NA/ 40.4 | 16/ 16 weeks | Schizophrenia (60.7%)  Schizoaffective disorder (39.3%) | DSM-IV | 51.1 (12.6) | 1/ Individual | NA |
|  | PE | 13/ NA/ 40.4 |  |  |  |  |  |  |
| Trower 2005/ UK/ SB | CBT | 18/ 10/ 36.6 | Median 16/ 6 months | Schizophrenia (71.1%)  Schizoaffective disorder (10.5%)  Psychotic depression (7.9%)  Personality disorder (7.9%) | ICD-10 | NA | 1/ Individual | 31.6% |
|  | TAU | 20/ 14/ 35.1 |  |  |  |  |  |  |
| Valmaggia 2005/ Netherlands/ assessor- blind | CBT | 35/ 27/ 35.4 | 16/ 22 weeks | Schizophrenia (100%) | DSM-IV | 65.49 (5.9) | 1/ Individual | 41% |
|  | SPT | 23/ 14/ 35.5 |  |  |  | 65.31 (5.4) |  |  |
| Wykes 2005 UK/ assessor-blinded | CBT | 45/ 24/ 39.7 | 7/ 10 weeks | Schizophrenia (100%) | DSM-IV | NA | 1/ Group | 28% |
|  | TAU | 40/ 26/ 39.7 |  |  |  |  |  |  |
| Mclead 2007/ UK/ OL | CBT | 10/ NA/ NA | 8/ 8 weeks | Schizophrenia (100%) | DSM-IV | NA | 1/ Group | NA |
|  | TAU | 10/ NA/ NA |  |  |  |  |  |  |
| Handdock 2009/ UK/ SBRCT | CBT | 38/ NA/ 35.7 | 17/ 6 months | Schizophrenia (89.6%)  Schizoaffective disorder (9.1%)  Psychosis NOS (1.3%) | DSM-IV | 60.0 (15.1) | 1/ Individual | NA |
|  | SAT | 39/ NA/ 33.9 |  |  |  | 62.8 (12.8) |  |  |
| Penn 2009/ USA/ assessor-blinded | CBT | 32/ 17/ 41.7 | 12/ 12 weeks | Schizophrenia (49.2%)  Schizoaffective disorder (50.8%) | Clinical diagnosis | 59.6 (10.8) | 3/ Group | 100% |
|  | SPT | 33/ 16/ 39.6 |  |  |  | 63.9 (9.7) |  |  |
| Shawyer 2012/ Australia/ assessor-blinded | CBT | 21/ 15/ 40.0 | 15/ 15 weeks | Schizophrenia (72.1%)  Schizoaffective disorder (20.9%)  Mood disorders with psychotic features (7.0%) | DSM-IV | 67.9 (13.6) | 1/ Individual | NA |
|  | SPT | 22/ 9/ 39.6 |  |  |  | 58.1 (8.4) |  |  |
| Krakvik 2013/ Norway/ OL | CBT | 23/ 15/ 35.3 | 20/ 6 months | Schizophrenia (61.8%)  Schizoaffective disorder (3.0%)  Delusional disorder (16.4%) | ICD-10 | NA | 1/ Individual | NA |
|  | TAU | 22/ 14/ 37.5 |  |  |  |  |  |  |
| Leff 2013  / UK/ SBCO | AVATAR | 26/ 16/ Na | 6/ 7 weeks | Schizophrenia (100%) | Clinical diagnosis | NA | 1/ Individual | NA |
|  | TAU |  |  |  |  |  |  |  |
| Rathod 2013/ UK/ SB | CBT | 17/ 10/ 31.4 | 16/ 16 weeks | Schizophrenia (NA)  Schizoaffective disorder (NA)  Delusional disorder (NA) | ICD-10 | NA | 1/ Individual | 18% |
|  | TAU | 18/ 10/ 35.6 |  |  |  |  |  |  |
| Lee 2014/ Korea/ assessor-blinded | CBT | 18/ 11/ 42.0 | 20/ 32 weeks | Schizophrenia (88%)  Schizoaffective disorder (12%) | DSM-IV | 62.2 (NA) | 1/ Individual | 2.7% |
|  | SPT | 19/ 10/ 40.5 |  |  |  | 60.3 (NA) |  |  |
| Freeman 2015/ UK/ assessor-blinded | CBT | 24/ 16/ 39.6 | 8/ 12 weeks | Schizophrenia (66%)  Schizoaffective disorder (20%)  Psychosis NOS (14%) | Clinical diagnosis | 83.6 (16.2) | 1/ Individual | NA |
|  | TAU | 26/ 18/ 42.2 |  |  |  | 79.7 (14.1) |  |  |
| Husain 2017/ Pakinstan/ assessor-blinded | CBT | 18/ 14/ 34.1 | 15/ 15 weeks | Schizophrenia spectrum disorder (F20-29) | ICD-10 | 61.4 (19.3) | 1/ Individual | NA |
|  | TAU | 18/ 10/ 30.5 |  |  |  | 60.9 (14.2) |  |  |
| Craig 2018  /UK/ SB | AVATAR | 75/ 57/ 42.5 | 6/ 12 weeks | Schizophrenia (77%)  Schizoaffective disorder (11%)  Bipolar disorder (5%)  Unspecific psychosis (5%)  Depression with psychotic symptoms (3%) | ICD-10 | NA | 1/ Individual | About 33% |
|  | SPT | 75/ 45/ 42.9 |  |  |  |  |  |  |
| Du Sert 2018  /Canada/ OL-D | AVATAR | 15/ 10/ 42.9 | 6/ 7 weeks | Schizophrenia (80%)  Schizoaffective disorder (20%) | DSM-5 | 76.7 (5.2) | 2/ Individual | 53.3% |
|  | TAU |  |  |  |  | 79.5 (5.8) |  |  |
| Morrison 2018/ UK/ assessor-blinded | CBT | 242/ 176/ 42.2 | 26/ 9 months | Schizophrenia (87.7%)  Schizoaffective disorder (9.9%)  Delusional disorder (1.4%)  Unspecific psychosis (0.6%) | ICD-10 | 82.8 (13.7) | 3/ Individual | 100% |
|  | TAU | 245/ 173/ 42.8 |  |  |  | 83.3 (14.0) |  |  |
| Wong 2018/ Hong Kong/ OL | CBT | 25/ 6/ 30.6 | 8/ 11 weeks | Schizophrenia (89.6%)  Schizoaffective disorder (2.1%)  Psychosis, NOS (8.3%) | DSM-5 | NA | 1/ Group | NA |
|  | PE | 23/ 11/ 35.1 |  |  |  |  |  |  |
| Mortan Sevi 2019/ Turkey/ assessor-blinded | CBT | 10/ NA/ 36.9 | 12 / 12 weeks | Schizophrenia (100%) | DSM-IV | NA | 1/ Group | NA |
|  | COPE-CBT | 12/ NA/ 36.9 |  |  |  |  |  |  |
|  | TAU | 10/ NA/ 36.9 |  |  |  |  |  |  |
| Dellazizzo 2021  / Canada/ OL | AVATAR | 37/ 29/ 43.6 | 9/ 9 weeks | Schizophrenia (77%)  Schizoaffective disorder (23%) | Clinical diagnosis | 79.0 (13.9) | 2/ Individual | 52.1% |
|  | CBT | 37/ 27/ 41.4 |  |  |  | 75.7 (15.7) |  |  |
| Liang 2022  / China/ OL | AVATAR | 32/ 14/ 25.3 | 6/ 7-9 weeks | Schizophrenia (100%) | Clinical diagnosis | 76.7 (15.4) | 2/ Individual | 43.1% |
|  | CBT | 33/ 17/ 26.5 |  |  |  | 74.9 (12.8) |  |  |
| Garaty 2024 /UK  / assessor-blinded | AVATAR-EX | 114/ 71/ 40.8 | 12/ 16 weeks | Schizophrenia (43.8%)  Schizoaffective disorder (7.8%)  Bipolar disorder (2.3%)  Unspecific psychosis (36.5%)  Depression with psychotic symptoms (9.6%) | ICD-10 | NA | 1/ Individual | About 25% |
|  | AVATAR-BR | 116/ 72/ 39.4 | 6/ 16 weeks |  |  |  |  |  |
|  | TAU | 115/ 79/ 38.7 | NA/ 16 weeks |  |  |  |  |  |

SBCO, Single-blinded cross-over; SB, Single-blinded; OL-D, open-label delayed; OL, open-label; SD, Standard deviation; AVATAR, Audio Visual Assisted Therapy Aid for Refractory auditory hallucinations; TAU, Treatment as usual; CBT, Cognitive behavioral therapy; SPT, Supportive psychotherapy; PE, Psychoeducation; SAT, Social activity therapy; EX, Extend; BR, Brief; NA, not applicable; DSM-(III, IV, 5), Diagnostic and Statistical Manual of Mental Disorders, (Third, Fourth, Fifth) Edition; ICD-10, International Classification of Diseases, Tenth Revision

Treatment resistant definition level 1: Persistence of psychotic symptoms, implying that the participants received some antipsychotics, without additional details.

Treatment resistant definition level 2: Failure of two or more antipsychotic treatments

Treatment resistant definition level 3: Failure of clozapine treatment

eTable 2. Details of network meta-analysis estimates for severity of treatment-resistant auditory hallucinations

Abbreviations: AVATAR = audio visual assisted therapy aid for refractory auditory hallucinations; CBT = cognitive behavioral therapy; SAT = social activity therapy; SPT = supportive psychotherapy; TAU = treatment-as-usual

eTable 3. Details of network meta-analysis estimates for long-term follow-up effects on treatment-resistant auditory hallucinations

Abbreviations: AVATAR = audio visual assisted therapy aid for refractory auditory hallucinations; CBT = cognitive behavioral therapy; SAT = social activity therapy; SPT = supportive psychotherapy; TAU = treatment-as-usual

eTable 4. Details of network meta-analysis estimates for overall psychotic symptoms measured by PANSS total score

Abbreviations: AVATAR = audio visual assisted therapy aid for refractory auditory hallucinations; CBT = cognitive behavioral therapy; PANSS = Positive and Negative Syndrome Scale for Schizophrenia; SAT = social activity therapy; SPT = supportive psychotherapy; TAU = treatment-as-usual

eTable 5. Details of network meta-analysis estimates for positive psychotic symptoms measured by PANSS

Abbreviations: AVATAR = audio visual assisted therapy aid for refractory auditory hallucinations; CBT = cognitive behavioral therapy; PANSS = Positive and Negative Syndrome Scale for Schizophrenia; SAT = social activity therapy; SPT = supportive psychotherapy; TAU = treatment-as-usua

eTable 6. Details of network meta-analysis estimates for negative psychotic symptoms measured by PANSS

Abbreviations: AVATAR = audio visual assisted therapy aid for refractory auditory hallucinations; CBT = cognitive behavioral therapy; PANSS = Positive and Negative Syndrome Scale for Schizophrenia; SAT = social activity therapy; SPT = supportive psychotherapy; TAU = treatment-as-usua

eTable 7. Details of network meta-analysis estimates for depressive symptoms

Abbreviations: AVATAR = audio visual assisted therapy aid for refractory auditory hallucinations; CBT = cognitive behavioral therapy; SAT = social activity therapy; SPT = supportive psychotherapy; TAU = treatment-as-usual

eTable 8. Details of network meta-analysis estimates for anxiety symptoms


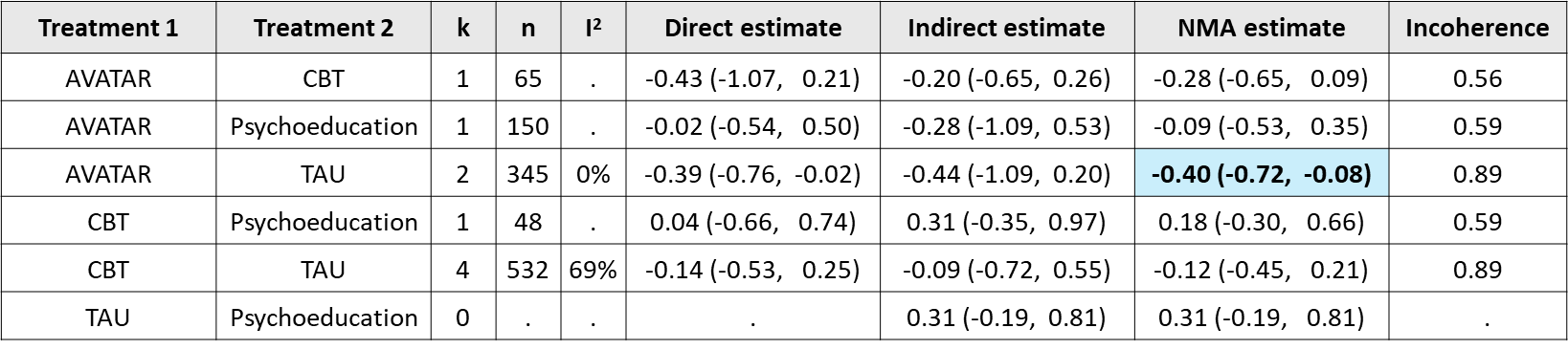


Abbreviations: AVATAR = audio visual assisted therapy aid for refractory auditory hallucinations; CBT = cognitive behavioral therapy; SAT = social activity therapy; TAU = treatment-as-usual

eTable 9. Details of network meta-analysis estimates for quality of life

Abbreviations: AVATAR = audio visual assisted therapy aid for refractory auditory hallucinations; CBT = cognitive behavioral therapy; SAT = social activity therapy; TAU = treatment-as-usual

eTable 10. Details of network meta-analysis estimates for all-cause discontinuation

Abbreviations: AVATAR = audio visual assisted therapy aid for refractory auditory hallucinations; CBT = cognitive behavioral therapy; SAT = social activity therapy; SPT = supportive psychotherapy; TAU = treatment-as-usual

eTable 11. SUCRA value of each treatment for severity of auditory hallucinations

|  | SUCRA |
| --- | --- |
| AVATAR | 0.95 |
| CBT | 0.73 |
| SAT | 0.43 |
| TAU | 0.42 |
| SPT | 0.26 |
| Psychoeducation | 0.22 |

Abbreviations: AVATAR = audio visual assisted therapy aid for refractory auditory hallucinations; CBT = cognitive behavioral therapy; SAT = social activity therapy; SPT = supportive psychotherapy; SUCRA = surface under the cumulative ranking curve; TAU = treatment-as-usual

eTable 12. SUCRA value of each treatment for long-term follow-up effects on treatment-resistant auditory hallucinations

|  | SUCRA |
| --- | --- |
| AVATAR | 0.89 |
| SPT | 0.73 |
| Psychoeducation | 0.57 |
| CBT | 0.39 |
| SAT | 0.21 |
| TAU | 0.21 |

Abbreviations: AVATAR = audio visual assisted therapy aid for refractory auditory hallucinations; CBT = cognitive behavioral therapy; SAT = social activity therapy; SPT = supportive psychotherapy; SUCRA = surface under the cumulative ranking curve; TAU = treatment-as-usual

eTable 13. SUCRA value of each treatment for overall psychotic symptoms measured by PANSS

|  | SUCRA |
| --- | --- |
| AVATAR | 0.98 |
| CBT | 0.60 |
| SAT | 0.46 |
| SPT | 0.40 |
| TAU | 0.07 |

Abbreviations: AVATAR = audio visual assisted therapy aid for refractory auditory hallucinations; CBT = cognitive behavioral therapy; PANSS = Positive and Negative Syndrome Scale for Schizophrenia; SAT = social activity therapy; SPT = supportive psychotherapy; SUCRA = surface under the cumulative ranking curve; TAU = treatment-as-usual

eTable 14. SUCRA value of each treatment for positive psychotic symptoms measured by PANSS

|  | SUCRA |
| --- | --- |
| AVATAR | 0.93 |
| Psychoeducation | 0.59 |
| SAT | 0.58 |
| CBT | 0.57 |
| SPT | 0.27 |
| TAU | 0.06 |

Abbreviations: AVATAR = audio visual assisted therapy aid for refractory auditory hallucinations; CBT = cognitive behavioral therapy; PANSS = Positive and Negative Syndrome Scale for Schizophrenia; SAT = social activity therapy; SPT = supportive psychotherapy; SUCRA = surface under the cumulative ranking curve; TAU = treatment-as-usual

eTable 15. SUCRA value of each treatment for negative psychotic symptoms measured by PANSS

|  | SUCRA |
| --- | --- |
| Psychoeducation | 0.68 |
| SPT | 0.68 |
| CBT | 0.61 |
| AVATAR | 0.47 |
| TAU | 0.32 |
| SAT | 0.23 |

Abbreviations: AVATAR = audio visual assisted therapy aid for refractory auditory hallucinations; CBT = cognitive behavioral therapy; PANSS = Positive and Negative Syndrome Scale for Schizophrenia; SAT = social activity therapy; SPT = supportive psychotherapy; SUCRA = surface under the cumulative ranking curve; TAU = treatment-as-usual

eTable 16. SUCRA value of each treatment for depressive symptoms

|  | SUCRA |
| --- | --- |
| CBT | 0.75 |
| AVATAR | 0.72 |
| TAU | 0.52 |
| Psychoeducation | 0.49 |
| SPT | 0.02 |

Abbreviations: AVATAR = audio visual assisted therapy aid for refractory auditory hallucinations; CBT = cognitive behavioral therapy; SPT = supportive psychotherapy; SUCRA = surface under the cumulative ranking curve; TAU = treatment-as-usual

eTable 17. SUCRA value of each treatment for anxiety symptoms

|  | SUCRA |
| --- | --- |
| AVATAR | 0.86 |
| Psychoeducation | 0.67 |
| CBT | 0.36 |
| TAU | 0.12 |

Abbreviations: AVATAR = audio visual assisted therapy aid for refractory auditory hallucinations; CBT = cognitive behavioral therapy; SUCRA = surface under the cumulative ranking curve; TAU = treatment-as-usual

eTable 18. SUCRA value of each treatment for quality of life

|  | SUCRA |
| --- | --- |
| AVATAR | 0.84 |
| CBT | 0.59 |
| Psychoeducation | 0.48 |
| SPT | 0.43 |
| TAU | 0.16 |

Abbreviations: AVATAR = audio visual assisted therapy aid for refractory auditory hallucinations; CBT = cognitive behavioral therapy; SPT = supportive psychotherapy; SUCRA = surface under the cumulative ranking curve; TAU = treatment-as-usual

eTable 19. SUCRA value of each treatment for all-cause discontinuation

|  | SUCRA |
| --- | --- |
| CBT | 0.72 |
| SPT | 0.60 |
| TAU | 0.57 |
| Psychoeducation | 0.33 |
| AVATAR | 0.28 |

Abbreviations: AVATAR = audio visual assisted therapy aid for refractory auditory hallucinations; CBT = cognitive behavioral therapy; SUCRA = surface under the cumulative ranking curve; TAU = treatment-as-usual

Appendix 1. The Preferred Reporting Items for Systematic Reviews and Meta­analyses (PRISMA) extension statement for reporting systematic reviews incorporating network meta-analysis

| **Section and Topic** | **Item #** | **Checklist item** | **Location where item is reported** |
| --- | --- | --- | --- |
| **TITLE** | | |  |
| Title | 1 | Identify the report as a systematic review incorporating a network meta-analysis | Title page |
| **ABSTRACT** | | |  |
| Abstract | 2 | Provide a structured summary including, as applicable:  Background: main objectives  Methods: data sources; study eligibility criteria, participants, and interventions; study appraisal;  and synthesis methods, such as network meta-analysis.  Results: number of studies and participants identified; summary estimates with corresponding  confidence/credible intervals; treatment rankings may also be discussed. Authors may choose  to summarize pairwise comparisons against a chosen treatment included in their analyses for  brevity.  Discussion/Conclusions: limitations; conclusions and implications of findings.  Other: primary source of funding; systematic review registration number with registry name | Abstract section |
| **INTRODUCTION** | | |  |
| Rationale | 3 | Describe the rationale for the review in the context of what is already known, including mention of  why a network meta-analysis has been conducted.. | 1^st^ – 3^rd^ paragraph of Introduction |
| Objectives | 4 | Provide an explicit statement of questions being addressed, with reference to participants,  interventions, comparisons, outcomes, and study design (PICOS). | 4^th^ paragraph of Introduction |
| **METHODS** | | |  |
| Protocol and  registration | 5 | Indicate whether a review protocol exists and if and where it can be accessed (e.g., Web address); and, if available, provide registration information, including registration number | PROSPERO (CRD42023469014) |
| Eligibility criteria | 6 | Specify study characteristics (e.g., PICOS, length of follow-up) and report characteristics (e.g.,  years considered, language, publication status) used as criteria for eligibility, giving rationale.  Clearly describe eligible treatments included in the treatment network, and note whether any  have been clustered or merged into the same node (with justification). | paragraph of “Study selection” of Method section |
| Information sources | 7 | Describe all information sources (e.g., databases with dates of coverage, contact with study authors to identify additional studies) in the search and date last searched | paragraph of Data sources and searches of Method section |
| Search | 8 | Present full electronic search strategy for at least one database, including any limits used, such that it could be repeated | paragraph of Data sources and searches of Method section |
| Selection process | 9 | State the process for selecting studies (i.e., screening, eligibility, included in systematic review,  and, if applicable, included in the meta-analysis). | paragraph of Study selection of Method section |
| Data collection process | 10 | Describe method of data extraction from reports (e.g., piloted forms, independently, in duplicate)  and any processes for obtaining and confirming data from investigators | paragraph of Study selection of Method section |
| Data items | 11 | List and define all variables for which data were sought (e.g., PICOS, funding sources) and any  assumptions and simplifications made | paragraph of Study selection of Method section |
| Geometry of the network | S1 | Describe methods used to explore the geometry of the treatment network under study and  potential biases related to it. This should include how the evidence base has been graphically  summarized for presentation, and what characteristics were compiled and used to describe  the evidence base to readers | paragraph of Definition of outcomes, data extraction, and risk of bias assessment of Method section |
| Study risk of bias within individual studies | 12 | Describe methods used for assessing risk of bias of individual studies (including specification of  whether this was done at the study or outcome level), and how this information is to be used  in any data synthesis. | paragraph of Definition of outcomes, data extraction, and risk of bias assessment of Method section |
| Summary measures | 13 | State the principal summary measures (e.g., risk ratio, difference in means). Also describe the use  of additional summary measures assessed, such as treatment rankings and surface under the  cumulative ranking curve (SUCRA) values, as well as modified approaches used to present  summary findings from meta-analyses. | paragraph of Data synthesis of Method section |
| Planned methods of analysis | 14 | Describe the processes used to decide which studies were eligible for each synthesis (e.g. tabulating the study intervention characteristics and comparing against the planned groups for each synthesis (item #5)). | paragraph of Data synthesis of Method section |
| Assessment of  inconsistency | S2 | Describe the statistical methods used to evaluate the agreement of direct and indirect evidence in  the treatment network(s) studied. Describe efforts taken to address its presence when found | paragraph of Assessment certainty of evidence for the primary outcome of Method section |
| Risk of bias across studies | 15 | Specify any assessment of risk of bias that may affect the cumulative evidence (e.g., publication  bias, selective reporting within studies). | paragraph of Definition of outcomes, data extraction, and risk of bias assessment of Method section |
| Additional analyses | 16 | Describe methods of additional analyses if done, indicating which were prespecified. This may  include, but not be limited to, the following: Sensitivity or subgroup analyses; Meta-regression analyses; Alternative formulations of the treatment network; and Use of alternative prior distributions for Bayesian analyses (if applicable). | paragraph of Data synthesis of Method section |
| **RESULTS** | | |  |
| Study selection | 17 | Give numbers of studies screened, assessed for eligibility, and included in the review, with reasons for exclusions at each stage, ideally with a flow diagram. | e-Figure 1 |
| Presentation of  network structure | S3 | Provide a network graph of the included studies to enable visualization of the geometry of the  treatment network. | Figure 1 |
| Summary of network geometry | S4 | Provide a brief overview of characteristics of the treatment network. This may include commentary  on the abundance of trials and randomized patients for the different interventions and pairwise comparisons in the network, gaps of evidence in the treatment network, and potential biases reflected by the network structure. | paragraph of Network meta-analysis of Result section |
| Study characteristics | 18 | For each study, present characteristics for which data were extracted (e.g., study size, PICOS,  follow-up period) and provide the citations. | e-Table 1 |
| Risk of bias within studies | 19 | Present data on risk of bias of each study and, if available, any outcome level assessment. | e-Figure 2, e-Figure 3 |
| Results of individual studies | 20 | For all outcomes considered (benefits or harms), present, for each study: 1) simple summary data  for each intervention group, and 2) effect estimates and confidence intervals. Modified approaches may be needed to deal with information from larger networks. | e-Figure 4 |
| Syntheses of results | 21 | Present results of each meta-analysis done, including confidence/credible intervals. In larger  networks, authors may focus on comparisons versus a particular comparator (e.g., placebo or  standard care), with full findings presented in an appendix. League tables and forest plots may  be considered to summarize pairwise comparisons. If additional summary measures were  explored (such as treatment rankings), these should also be presented | Figure 2, paragraph of Network meta-analysis of Result section |
| Reporting biases | 21 | Present assessments of risk of bias due to missing results (arising from reporting biases) for each synthesis assessed. | e-Figure 2, e-Figure 3, paragraph of Quality of the included studies of Result section |
| Exploration for  inconsistency | S5 | Describe results from investigations of inconsistency. This may include such information as  measures of model fit to compare consistency and inconsistency models, P values from  statistical tests, or summary of inconsistency estimates from different parts of the treatment  network. | paragraph of Transitivity assumption of Result section, eFigure 5-6 |
| Risk of bias across studies | 22 | Present results of any assessment of risk of bias across studies for the evidence base being  studied. | e-Figure 2, e-Figure 3 |
| Results of additional  analyses | 23 | Give results of additional analyses, if done (e.g., sensitivity or subgroup analyses, meta-regression analyses, alternative network geometries studied, alternative choice of prior distributions for Bayesian analyses, and so forth). | Figure 3, Figure 4, paragraph of Sensitivity analyses of Result section, Figure 3, eFigure 8-10 |
| **DISCUSSION** | | |  |
| Summary of evidence | 24 | Summarize the main findings, including the strength of evidence for each main outcome; consider their relevance to key groups (e.g., health care providers, researchers, and policymakers). | 1^st^ paragraph of Discussion |
| Limitations | 25 | Discuss limitations at study and outcome level (e.g., risk of bias), and at review level (e.g.,  incomplete retrieval of identified research, reporting bias). Comment on the validity of the  assumptions, such as transitivity and consistency. Comment on any concerns regarding  network geometry (e.g., avoidance of certain comparisons). | The paragraph of Strengths and limitations of this study of Discussion |
| Conclusions | 26 | Provide a general interpretation of the results in the context of other evidence, and implications  for future research. | The paragraph of Implications and conclusions Discussion |
| **OTHER INFORMATION** | | |  |
| Funding | 27 | Describe sources of funding for the systematic review and other support (e.g., supply of data); role  of funders for the systematic review. This should also include information regarding whether  funding has been received from manufacturers of treatments in the network and/or whether  some of the authors are content experts with professional conflicts of interest that could affect  use of treatments in the network. | Bottom of the manuscript |

Appendix 2. The complete search strategies

| Database |
| --- |
| PubMed search strategy |
| #1 "voice*"[All Fields] OR (("auditorially"[All Fields] OR "auditory"[All Fields]) AND ("verbal"[All Fields] OR "verbalization"[All Fields] OR "verbalizations"[All Fields] OR "verbalize"[All Fields] OR "verbalized"[All Fields] OR "verbalizer"[All Fields] OR "verbalizers"[All Fields] OR "verbalizing"[All Fields] OR "verbally"[All Fields]) AND ("hallucinations"[MeSH Terms] OR "hallucinations"[All Fields] OR "hallucinate"[All Fields] OR "hallucinated"[All Fields] OR "hallucinating"[All Fields] OR "hallucination"[All Fields] OR "hallucinative"[All Fields] OR "hallucinator"[All Fields] OR "hallucinators"[All Fields])) OR "AVH"[All Fields] OR ("schizophrenia"[MeSH Terms] OR "schizophrenia"[All Fields] OR "schizophrenias"[All Fields] OR "schizophrenia s"[All Fields]) OR "schizo*"[All Fields] OR ("psychotic disorders"[MeSH Terms] OR ("psychotic"[All Fields] AND "disorders"[All Fields]) OR "psychotic disorders"[All Fields] OR "psychosis"[All Fields]) (333,656)  #2 "behaviour therapy"[All Fields] OR "behavior therapy"[MeSH Terms] OR ("behavior"[All Fields] AND "therapy"[All Fields]) OR "behavior therapy"[All Fields] OR ("behaviour therapy"[All Fields] OR "behavior therapy"[MeSH Terms] OR ("behavior"[All Fields] AND "therapy"[All Fields]) OR "behavior therapy"[All Fields]) OR (("cognition"[MeSH Terms] OR "cognition"[All Fields] OR "cognitions"[All Fields] OR "cognitive"[All Fields] OR "cognitively"[All Fields] OR "cognitives"[All Fields]) AND ("behavior"[MeSH Terms] OR "behavior"[All Fields] OR "behavioral"[All Fields] OR "behavioural"[All Fields] OR "behavior s"[All Fields] OR "behaviorally"[All Fields] OR "behaviour"[All Fields] OR "behaviourally"[All Fields] OR "behaviours"[All Fields] OR "behaviors"[All Fields] OR "pattern"[All Fields] OR "pattern s"[All Fields] OR "patternability"[All Fields] OR "patternable"[All Fields] OR "patterned"[All Fields] OR "patterning"[All Fields] OR "patternings"[All Fields] OR "patterns"[All Fields])) OR (("cognition"[MeSH Terms] OR "cognition"[All Fields] OR "cognitions"[All Fields] OR "cognitive"[All Fields] OR "cognitively"[All Fields] OR "cognitives"[All Fields]) AND "behaviorial"[All Fields]) OR (("cognition"[MeSH Terms] OR "cognition"[All Fields] OR "cognitions"[All Fields] OR "cognitive"[All Fields] OR "cognitively"[All Fields] OR "cognitives"[All Fields]) AND ("intervention s"[All Fields] OR "interventions"[All Fields] OR "interventive"[All Fields] OR "methods"[MeSH Terms] OR "methods"[All Fields] OR "intervention"[All Fields] OR "interventional"[All Fields])) OR ("cognitive behavioral therapy"[MeSH Terms] OR ("cognitive"[All Fields] AND "behavioral"[All Fields] AND "therapy"[All Fields]) OR "cognitive behavioral therapy"[All Fields] OR ("cognitive"[All Fields] AND "therapy"[All Fields]) OR "cognitive therapy"[All Fields]) OR (("virtual"[All Fields] OR "virtuality"[All Fields] OR "virtualization"[All Fields] OR "virtualized"[All Fields] OR "virtualizing"[All Fields] OR "virtuals"[All Fields]) AND "real*"[All Fields]) OR ("avatar"[MeSH Terms] OR "avatar"[All Fields] OR "avatars"[All Fields] OR "avatar s"[All Fields]) OR (("virtual"[All Fields] OR "virtuality"[All Fields] OR "virtualization"[All Fields] OR "virtualized"[All Fields] OR "virtualizing"[All Fields] OR "virtuals"[All Fields]) AND "environ*"[All Fields]) OR (("virtual"[All Fields] OR "virtuality"[All Fields] OR "virtualization"[All Fields] OR "virtualized"[All Fields] OR "virtualizing"[All Fields] OR "virtuals"[All Fields]) AND "character*"[All Fields]) (860,616)  #3 #1 AND #2 (44,422)  #4 #3 AND (randomized controlled trial[Filter]) (2,912) |
| Cochrane Central Register of Controlled Trials (CENTRAL) search strategy |
| #1 (auditory verbal hallucinations):ti,ab,kw (Word variations have been searched) (207)  #2 MeSH descriptor: [Schizophrenia] explode all trees (9800)  #3 schizophrenia (21066)  #4 MeSH descriptor: [Psychotic Disorders] explode all trees (4042)  #5 (psychosis):ti,ab,kw (Word variations have been searched) (7852)  #6 MeSH descriptor: [Voice] explode all trees (593)  #7 (voice):ti,ab,kw (Word variations have been searched) (6153)  #8 #1 OR #2 OR #3 OR #4 OR #5 OR #6 OR #7 (31596)  #9 MeSH descriptor: [Cognitive Behavioral Therapy] explode all trees (14291)  #10 MeSH descriptor: [Avatar] explode all trees (7)  #11 (avatar):ti,ab,kw (Word variations have been searched) (532)  #12 MeSH descriptor: [Virtual Reality] explode all trees (1343)  #13 ("virtual reality therapy"):ti,ab,kw (Word variations have been searched) (164)  #14 (virtual environment):ti,ab,kw (Word variations have been searched) (2493)  #15 (virtual character):ti,ab,kw (Word variations have been searched) (94)  #16 #9 OR #10 OR #11 OR #12 OR #13 OR #14 OR #15 (18378)  #17 #8 AND #16 (1098) |
| Embase search strategy |
| #1. 'auditory verbal hallucinations' OR avh (2,007)  #2. 'schizophrenia'/exp OR 'schizophrenia' (272,849)  #3. 'psychosis'/exp OR 'psychosis' (389,494)  #4. 'voice'/exp OR 'voice' (71,491)  #5. #1 OR #2 OR #3 OR #4 (484,748)  #6. 'behavior therapy'/exp OR 'behavior therapy' (95,069)  #7. 'cognitive behavioral therapy'/exp OR 'cognitive behavioral therapy' (45,855)  #8. 'avatar'/exp OR avatar (3,041)  #9. 'virtual reality'/exp OR 'virtual reality' (41,106)  #10. 'virtual environment'/exp OR 'virtual environment' (4,555)  #11. 'virtual character' (106)  #12. #6 OR #7 OR #8 OR #9 OR #10 OR #11 (145,992)  #13. #5 AND #12 (8,455)  #14. #13 AND 'randomized controlled trial'/de (926) |
| PsycInfo search strategy |
| S1 (auditory verbal hallucinations OR AVH OR schizophrenia OR psychosis) AND (behavior therapy OR behaviour therapy OR cognitive behavior OR cognitive behaviorial OR cognitive intervention OR cognitive therapy OR virtual OR AVATAR) AND (randomized controlled trials or rtc or randomised control trials)  (571) |
| ClinicalTrials.gov (https://clinicaltrials.gov/) |
| auditory verbal hallucinations \| AVATAR (6)  auditory verbal hallucinations \| cognitive behavioral (5) |

CENTRAL: Cochrane Central Register of Controlled Trials

Appendix 3. Reasons for exclusion

**No outcome of interest (n=25)**

1. A Pilot Randomized Controlled Study to Compare Avatar Therapy and Cognitive Behavioral Therapy in Patients With Treatment-resistant Schizophrenia [Internet]. 2018. Available from: https://clinicaltrials.gov/study/NCT03585127.

2. Addington J, Epstein I, Liu L, French P, Boydell KM, Zipursky RB. A randomized controlled trial of cognitive behavioral therapy for individuals at clinical high risk of psychosis. Schizophr Res. 2011;125(1):54-61.

3. Beaudoin M, Potvin S, Phraxayavong K, Dumais A. Changes in Quality of Life in Treatment-Resistant Schizophrenia Patients Undergoing Avatar Therapy: A Content Analysis. Journal of Personalized Medicine. 2023;13(3).

4. Birchwood M, Dunn G, Meaden A, Tarrier N, Lewis S, Wykes T, et al. The COMMAND trial of cognitive therapy to prevent harmful compliance with command hallucinations: predictors of outcome and mediators of change. Psychol Med. 2018;48(12):1966-74.

5. Birchwood M, Mohan L, Meaden A, Tarrier N, Lewis S, Wykes T, et al. The COMMAND trial of cognitive therapy for harmful compliance with command hallucinations (CTCH): a qualitative study of acceptability and tolerability in the UK. BMJ Open. 2018;8(6):e021657.

6. Bortolon C, Dorahy MJ, Brand R, Dondé C, Slovak S, Raffard S. The effect of voice content and social context on shame: a simulation and vignette paradigm to evaluate auditory verbal hallucinations. Cogn Neuropsychiatry. 2022;27(2-3):122-38.

7. England M. Significance of cognitive intervention for voice hearers. Perspectives in Psychiatric Care. 2008;44(1):40-7.

8. Freeman D, Dunn G, Startup H, Pugh K, Cordwell J, Mander H, et al. Effects of cognitive behaviour therapy for worry on persecutory delusions in patients with psychosis (WIT): a parallel, single-blind, randomised controlled trial with a mediation analysis. Lancet Psychiatry. 2015;2(4):305-13.

9. Granholm E, Holden J, Link PC, McQuaid JR, Jeste DV. Randomized controlled trial of cognitive behavioral social skills training for older consumers with schizophrenia: defeatist performance attitudes and functional outcome. Am J Geriatr Psychiatry. 2013;21(3):251-62.

10. Gumley A, O'Grady M, McNay L, Reilly J, Power K, Norrie J. Early intervention for relapse in schizophrenia: results of a 12-month randomized controlled trial of cognitive behavioural therapy. Psychol Med. 2003;33(3):419-31.

11. Johnson DP, Penn DL, Bauer DJ, Meyer P, Evans E. Predictors of the therapeutic alliance in group therapy for individuals with treatment-resistant auditory hallucinations. Br J Clin Psychol. 2008;47(Pt 2):171-83.

12. Lecomte T, Leclerc C, Wykes T. Group cbt for early psychosis-Are there still benefits one year later? International Journal of Group Psychotherapy. 2012;62(2):309-21.

13. Lee BM, Kim SW, Lee BJ, Won SH, Park YH, Kang CY, et al. Effects and safety of virtual reality-based mindfulness in patients with psychosis: a randomized controlled pilot study. Schizophrenia. 2023;9(1).

14. Lee KH. A randomized controlled trial of mindfulness in patients with schizophrenia(✰). Psychiatry Res. 2019;275:137-42.

15. Li S, Liu R, Sun B, Wei N, Shen Z, Xu Y, et al. Effect of Virtual Reality on Cognitive Impairment and Clinical Symptoms among Patients with Schizophrenia in the Remission Stage: A Randomized Controlled Trial. Brain Sciences. 2022;12(11).

16. Lincoln TM, Ziegler M, Mehl S, Kesting ML, Lüllmann E, Westermann S, et al. Moving from efficacy to effectiveness in cognitive behavioral therapy for psychosis: a randomized clinical practice trial. J Consult Clin Psychol. 2012;80(4):674-86.

17. Rector NA, Seeman MV, Segal ZV. Cognitive therapy for schizophrenia: a preliminary randomized controlled trial. Schizophr Res. 2003;63(1-2):1-11.

18. Sensky T, Turkington D, Kingdon D, Scott JL, Scott J, Siddle R, et al. A randomized controlled trial of cognitive-behavioral therapy for persistent symptoms in schizophrenia resistant to medication. Arch Gen Psychiatry. 2000;57(2):165-72.

19. Simón-Expósito M, Felipe-Castaño E. Effects of Metacognitive Training on Cognitive Insight in a Sample of Patients with Schizophrenia. Int J Environ Res Public Health. 2019;16(22).

20. Tarrier N, Kinney C, McCarthy E, Humphreys L, Wittkowski A, Morris J. Two-year follow-up of cognitive--behavioral therapy and supportive counseling in the treatment of persistent symptoms in chronic schizophrenia. J Consult Clin Psychol. 2000;68(5):917-22.

21. Tarrier N, Yusupoff L, Kinney C, McCarthy E, Gledhill A, Haddock G, et al. Randomised controlled trial of intensive cognitive behaviour therapy for patients with chronic schizophrenia. Bmj. 1998;317(7154):303-7.

22. Tay JL, Qu Y, Lim L, Puthran R, Tan CLR, Rajendran R, et al. Impact of a Virtual Reality Intervention on Stigma, Empathy, and Attitudes Toward Patients With Psychotic Disorders Among Mental Health Care Professionals: Randomized Controlled Trial. JMIR Ment Health. 2025;12:e66925.

23. Wiersma D, Jenner JA, Nienhuis FJ, van de Willige G. Hallucination focused integrative treatment improves quality of life in schizophrenia patients. Acta Psychiatr Scand. 2004;109(3):194-201.

24. Wong DFK, Cheung YCH, Oades LG, Ye SS, Ng YP. Strength-based cognitive-behavioural therapy and peer-to-peer support in the recovery process for people with schizophrenia: A randomised control trial. Int J Soc Psychiatry. 2024;70(2):364-77.

25. Zhu X, Song H, Chang R, Chen B, Song Y, Liu J, et al. Combining compensatory cognitive training and medication self-management skills training, in inpatients with schizophrenia: A three-arm parallel, single-blind, randomized controlled trial. Gen Hosp Psychiatry. 2021;69:94-103.

**No outcome report or incomplete data (n=5)**

1. A Randomized Controlled Trial Comparing Avatar Therapy to Cognitive Behavioral Therapy in Schizophrenia With Treatment Refractory Hallucinations [Internet]. 2019. Available from: https://clinicaltrials.gov/study/NCT04054778.

2. Remotely Delivered Avatar-mediated Therapy Versus Cognitive Behavioural Therapy for Persisting Auditory Hallucinations: Randomised Controlled Superiority Trial [Internet]. 2023. Available from: https://clinicaltrials.gov/study/NCT05982158.

3. VR-based Avatar Therapy for Treatment of Auditory Hallucinations in Patients With Schizophrenia 2023-SUD-3446 [Internet]. 2024. Available from: <https://clinicaltrials.gov/study/NCT06505564>.

4. Isrctn. AVATAR_VRSocial therapy for auditory verbal hallucinations in early psychosis. https://trialsearchwhoint/Trial2aspx?TrialID=ISRCTN35980117. 2023.

5. Stefaniak I, Sorokosz K, Janicki A, Wciorka J. Therapy based on avatar-therapist synergy for patients with chronic auditory hallucinations: a pilot study. Schizophrenia research. 2019;211:115‐7.

**Conference abstract, protocol, review, or comment (n=19)**

1. Beaudoin M, Potvin S, Dumais A. Comparison of VR-Assisted Therapy to Cognitive- Behavioral Therapy in the treatment of verbal hallucinations in patient with treatment-resistant schizophrenia. European Psychiatry. 2023;66:S127.

2. Birchwood M, Peters E, Tarrier N, Dunn G, Lewis S, Wykes T, et al. A multi-centre, randomised controlled trial of cognitive therapy to prevent harmful compliance with command hallucinations. BMC Psychiatry. 2011;11:155.

3. Cavelti M, Kaeser JM, Lerch S, Bauer S, Moessner M, Berger T, et al. Smartphone-assisted guided self-help cognitive behavioral therapy for young people with distressing voices (SmartVoices): study protocol for a randomized controlled trial. Trials. 2022;23(1).

4. Clemmensen L, Lund CN, Andresen BS, Midtgaard J, Glenthøj LB. Study protocol for RUFUS-A randomized mixed methods pilot clinical trial investigating the relevance and feasibility of rumination-focused cognitive behavioral therapy in the treatment of patients with emergent psychosis spectrum disorders. PloS one. 2024;19(1):e0297118.

5. Craig T, Garety P, Ward T, Edwards C, Rus-Calafell M, Huckvale M, et al. The UK AVATAR 1 and 2 Trials for People with Distressing Voices - Findings and Learning from AVATAR1, and AVATAR2 Developments in Theory and Therapy. European psychiatry. 2022;65:S22‐S3.

6. Craig T, Garety P, Ward T, Rus-Calafell M, Williams G, Huckvale M, et al. Computer assisted therapy for auditory hallucinations: The avatar clinical trial. Schizophrenia Research. 2014;153:S74.

7. Craig TK, Rus-Calafell M, Ward T, Fornells-Ambrojo M, McCrone P, Emsley R, et al. The effects of an Audio Visual Assisted Therapy Aid for Refractory auditory hallucinations (AVATAR therapy): study protocol for a randomised controlled trial. Trials. 2015;16:349.

8. Dellazizzo L, Potvin S, O'Connor K, Dumais A. A randomized controlled trial comparing virtual reality therapy to cognitive behavioral therapy in schizophrenia with treatment refractory hallucinations: Preliminary results. Schizophrenia Bulletin. 2018;44:S346-S7.

9. Dzafic I, Spark J, Bell I, Wood S, Lavoie S, Whitford T, et al. PAPER: The hybrid study: Integrating neurofeedback, virtual reality, and cognitive behaviour therapy for the treatment of hearing voices. Early Intervention in Psychiatry. 2023;17:141.

10. Garety P, Edwards CJ, Ward T, Emsley R, Huckvale M, McCrone P, et al. Optimising AVATAR therapy for people who hear distressing voices: study protocol for the AVATAR2 multi-centre randomised controlled trial. Trials. 2021;22(1).

11. Garety P, Edwards CJ, Ward T, Emsley R, Huckvale M, McCrone P, et al. Correction: optimising AVATAR therapy for people who hear distressing voices: study protocol for the AVATAR2 multi-centre randomised controlled trial. Trials. 2024;25(1):803.

12. Geraets C, Snippe E, Van Beilen M, Pot-Kolder R, Wichers M, Van Der Gaag M, et al. The effect of virtual reality cognitive behavioral therapy on paranoia and mood states. Schizophrenia Bulletin. 2020;46:S270.

13. Glenthøj LB. PAPER: Using immersive virtual reality in treating paranoia and auditory hallucinations. Early Intervention in Psychiatry. 2023;17:141-2.

14. Hayward M, Berry K, Bremner S, Jones AM, Robertson S, Cavanagh K, et al. Increasing access to cognitive–behavioural therapy for patients with psychosis by evaluating the feasibility of a randomised controlled trial of brief, targeted cognitive–behavioural therapy for distressing voices delivered by assistant psychologists: the GiVE2 trial. BJPsych Open. 2021;7(5).

15. Hazell CM, Hayward M, Cavanagh K, Jones AM, Strauss C. Guided self-help cognitive behavioral intervention for VoicEs (GiVE): study protocol for a pilot randomized controlled trial. Trials. 2016;17(1):351.

16. Klingberg S, Wittorf A, Meisner C, Wölwer W, Wiedemann G, Herrlich J, et al. Cognitive behavioural therapy versus supportive therapy for persistent positive symptoms in psychotic disorders: the POSITIVE Study, a multicenter, prospective, single-blind, randomised controlled clinical trial. Trials. 2010;11:123.

17. Leff J, Williams G, Huckvale M, Arbuthnot M, Leff AP. Avatar therapy for persecutory auditory hallucinations: what is it and how does it work? Psychosis. 2014;6(2):166‐76.

18. Smith LC, Mariegaard L, Vernal DL, Christensen AG, Albert N, Thomas N, et al. The CHALLENGE trial: the effects of a virtual reality-assisted exposure therapy for persistent auditory hallucinations versus supportive counselling in people with psychosis: study protocol for a randomised clinical trial. Trials. 2022;23(1).

19. Ster AC, Emsley R, Landau S. COMBINING NON-ADHERENCE AND MEDIATION IN A UNIFIED CAUSAL ANALYSIS: A METHODOLOGICAL REVIEW AND APPLICATION TO THE AVATAR TRIAL. Clinical Trials. 2023;20:54.

**Not meet inclusive criteria (n=7)**

1. Dodgson G, Alderson-Day B, Smailes D, Ryles F, Mayer C, Glen-Davison J, et al. Tailoring cognitive behavioural therapy to subtypes of voice-hearing using a novel tabletised manual: a feasibility study. Behavioural and cognitive psychotherapy. 2021;49(3):287-301.

2. Moritz S, Veckenstedt R, Andreou C, Bohn F, Hottenrott B, Leighton L, et al. Sustained and "sleeper" effects of group metacognitive training for schizophrenia: a randomized clinical trial. JAMA Psychiatry. 2014;71(10):1103-11.

3. O'Brien C, Rus-Calafell M, Craig TK, Garety P, Ward T, Lister R, et al. Relating behaviours and therapeutic actions during AVATAR therapy dialogue: An observational study. Br J Clin Psychol. 2021;60(4):443-62.

4. Rus-Calafell M, Ehrbar N, Ward T, Edwards C, Huckvale M, Walke J, et al. Participants' experiences of AVATAR therapy for distressing voices: a thematic qualitative evaluation. BMC Psychiatry. 2022;22(1):356.

5. van Oosterhout B, Krabbendam L, de Boer K, Ferwerda J, van der Helm M, Stant AD, et al. Metacognitive group training for schizophrenia spectrum patients with delusions: a randomized controlled trial. Psychol Med. 2014;44(14):3025-35.

6. Xue T, Sheng J, Gao H, Gu Y, Dai J, Yang X, et al. Eight-month intensive meditation-based intervention improves refractory hallucinations and delusions and quality of life in male inpatients with schizophrenia: a randomized controlled trial. Psychiatry Clin Neurosci. 2024;78(4):248-58.

7. Zanello A, Mohr S, Merlo MC, Huguelet P, Rey-Bellet P. Effectiveness of a brief group cognitive behavioral therapy for auditory verbal hallucinations: a 6-month follow-up study. J Nerv Ment Dis. 2014;202(2):144-53.

**Not directly interact with therapists (n=4)**

1. Eisner E, Berry N, Morris R, Emsley R, Haddock G, Machin M, et al. Exploring engagement with the CBT-informed Actissist smartphone application for early psychosis. J Ment Health. 2023;32(3):643-54.

2. Gottlieb JD, Gidugu V, Maru M, Tepper MC, Davis MJ, Greenwold J, et al. Randomized controlled trial of an internet cognitive behavioral skills-based program for auditory hallucinations in persons with psychosis. Psychiatr Rehabil J. 2017;40(3):283-92.

3. Hazell CM, Hayward M, Cavanagh K, Jones AM, Strauss C. Guided self-help cognitive-behaviour Intervention for VoicEs (GiVE): Results from a pilot randomised controlled trial in a transdiagnostic sample. Schizophr Res. 2018;195:441-7.

4. Naeem F, Johal R, McKenna C, Rathod S, Ayub M, Lecomte T, et al. Cognitive Behavior Therapy for psychosis based Guided Self-help (CBTp-GSH) delivered by frontline mental health professionals: Results of a feasibility study. Schizophr Res. 2016;173(1-2):69-74.

**Duplicated data (n=1)**

1. Isrctn. Optimising AVATAR therapy for distressing voices. https://trialsearchwhoint/Trial2aspx?TrialID=ISRCTN55682735. 2020.

Appendix 5. Node-splitting and global inconsistency results for severity of auditory hallucinations

| Node-splitting | k | Prop | NMA | Direct | Indir. | Diff | z | p-value |
| --- | --- | --- | --- | --- | --- | --- | --- | --- |
| AVATAR:CBT | 2 | 0.38 | -0.2259 | -0.3299 | -0.1634 | -0.1665 | -0.49 | 0.63 |
| AVATAR:Psychoeducation | 1 | 0.53 | -0.5991 | -0.4815 | -0.7335 | 0.2521 | 0.51 | 0.61 |
| AVATAR:TAU | 4 | 0.63 | -0.5269 | -0.5105 | -0.5544 | 0.0439 | 0.13 | 0.89 |
| CBT:Psychoeducation | 2 | 0.59 | -0.3732 | -0.4755 | -0.2234 | -0.2521 | -0.51 | 0.61 |
| CBT:SPT | 6 | 0.91 | -0.1865 | -0.1768 | -0.285 | 0.1083 | 0.2 | 0.84 |
| CBT:TAU | 14 | 0.84 | -0.301 | -0.3335 | -0.1362 | -0.1973 | -0.66 | 0.51 |
| SPT:TAU | 2 | 0.45 | -0.1145 | 0.0287 | -0.2322 | 0.2609 | 0.75 | 0.45 |
| Global inconsistency | Q |  |  |  |  |  |  | p-value |
| Between designs | 1.35 |  |  |  |  |  |  | 0.97 |

Abbreviations: AVATAR = audio visual assisted therapy aid for refractory auditory hallucinations; CBT = cognitive behavioral therapy; NMA = network meta-analysis; SPT = supportive psychotherapy; SUCRA = surface under the cumulative ranking curve; TAU = treatment-as-usual

Appendix 6. Node-splitting and global inconsistency results for long-term follow-up effects on treatment-resistant auditory hallucinations

| Node-splitting | k | Prop | NMA | Direct | Indir. | Diff | z | p-value |
| --- | --- | --- | --- | --- | --- | --- | --- | --- |
| AVATAR:CBT | 2 | 0.45 | -0.3689 | -0.4195 | -0.3283 | -0.0912 | -0.28 | 0.78 |
| AVATAR:TAU | 4 | 0.71 | -0.4717 | -0.445 | -0.5362 | 0.0912 | 0.28 | 0.78 |
| SPT:CBT | 5 | 0.92 | -0.2269 | -0.2295 | -0.1985 | -0.031 | -0.06 | 0.96 |
| TAU:CBT | 8 | 0.81 | 0.1029 | 0.1468 | -0.0865 | 0.2333 | 0.77 | 0.44 |
| SPT:TAU | 2 | 0.5 | -0.3297 | -0.2149 | -0.4455 | 0.2306 | 0.66 | 0.51 |
| Global inconsistency | Q |  |  |  |  |  |  | p-value |
| Between design | 1.13 |  |  |  |  |  |  | 0.77 |

Abbreviations: AVATAR = audio visual assisted therapy aid for refractory auditory hallucinations; CBT = cognitive behavioral therapy; NMA = network meta-analysis; SPT = supportive psychotherapy; TAU = treatment-as-usual

Appendix 7. Node-splitting and global inconsistency results for overall psychotic symptoms measured by PANSS

| Node-splitting | k | Prop | NMA | Direct | Indir. | Diff | z | p-value |
| --- | --- | --- | --- | --- | --- | --- | --- | --- |
| AVATAR:CBT | 2 | 0.89 | -0.4079 | -0.3131 | -1.1501 | 0.837 | 1.51 | 0.13 |
| AVATAR:TAU | 1 | 0.14 | -0.6694 | -1.3904 | -0.5534 | -0.837 | -1.51 | 0.13 |
| CBT:SPT | 6 | 0.91 | -0.0828 | -0.064 | -0.2758 | 0.2117 | 0.59 | 0.55 |
| CBT:TAU | 6 | 0.93 | -0.2616 | -0.2254 | -0.7179 | 0.4926 | 1.47 | 0.14 |
| TAU:SPT | 2 | 0.58 | 0.1787 | 0.1786 | 0.1789 | -0.0002 | 0 | 1 |
| Global inconsistency | Q |  |  |  |  |  |  | p-value |
| Between designs | 3.19 |  |  |  |  |  |  | 0.36 |

Abbreviations: AVATAR = audio visual assisted therapy aid for refractory auditory hallucinations; CBT = cognitive behavioral therapy; NMA = network meta-analysis; PANSS = Positive and Negative Syndrome Scale for Schizophrenia; SPT = supportive psychotherapy; TAU = treatment-as-usual

Appendix 8. Node-splitting and global inconsistency results for positive psychotic symptoms measured by PANSS

| Node-splitting | k | Prop | NMA | Direct | Indir. | Diff | z | p-value |
| --- | --- | --- | --- | --- | --- | --- | --- | --- |
| AVATAR:CBT | 2 | 0.77 | -0.2471 | -0.2857 | -0.1187 | -0.167 | -0.47 | 0.64 |
| AVATAR:Psychoeducation | 1 | 0.86 | -0.2043 | -0.1599 | -0.4866 | 0.3268 | 0.74 | 0.46 |
| AVATAR:TAU | 1 | 0.12 | -0.5197 | -0.5921 | -0.5097 | -0.0824 | -0.17 | 0.87 |
| CBT:Psychoeducation | 1 | 0.27 | 0.0428 | -0.1968 | 0.13 | -0.3268 | -0.74 | 0.46 |
| CBT:SPT | 5 | 0.9 | -0.1524 | -0.1602 | -0.0852 | -0.075 | -0.25 | 0.8 |
| CBT:TAU | 5 | 0.94 | -0.2725 | -0.262 | -0.4417 | 0.1797 | 0.6 | 0.55 |
| SPT:TAU | 1 | 0.56 | -0.1202 | -0.1666 | -0.0619 | -0.1047 | -0.5 | 0.62 |
| Global inconsistency | Q |  |  |  |  |  |  | p-value |
| Between designs | 0.93 |  |  |  |  |  |  | 0.92 |

Abbreviations: AVATAR = audio visual assisted therapy aid for refractory auditory hallucinations; CBT = cognitive behavioral therapy; NMA = network meta-analysis; PANSS = Positive and Negative Syndrome Scale for Schizophrenia; SPT = supportive psychotherapy; SUCRA = surface under the cumulative ranking curve; TAU = treatment-as-usual

Appendix 9. Node-splitting and global inconsistency results for negative psychotic symptoms measured by PANSS

| Node-splitting | k | Prop | NMA | Direct | Indir. | Diff | z | p-value |
| --- | --- | --- | --- | --- | --- | --- | --- | --- |
| AVATAR:CBT | 2 | 0.77 | 0.0438 | 0.0367 | 0.067 | -0.0303 | -0.09 | 0.93 |
| AVATAR:Psychoeducation | 1 | 0.86 | 0.0958 | 0.1082 | 0.0185 | 0.0896 | 0.2 | 0.84 |
| AVATAR:TAU | 1 | 0.13 | -0.053 | -0.0977 | -0.0461 | -0.0516 | -0.1 | 0.92 |
| CBT:Psychoeducation | 1 | 0.27 | 0.052 | -0.0135 | 0.0761 | -0.0896 | -0.2 | 0.84 |
| CBT:TAU | 4 | 0.97 | -0.0967 | -0.0951 | -0.1467 | 0.0516 | 0.1 | 0.92 |
| Global inconsistency | Q |  |  |  |  |  |  | p-value |
| Between design | 0.03 |  |  |  |  |  |  | 0.99 |

Abbreviations: AVATAR = audio visual assisted therapy aid for refractory auditory hallucinations; CBT = cognitive behavioral therapy; NMA = network meta-analysis; PANSS = Positive and Negative Syndrome Scale for Schizophrenia; TAU = treatment-as-usual

Appendix 10. Node-splitting and global inconsistency results for depressive symptoms

| Node-splitting | k | Prop | NMA | Direct | Indir. | Diff | z | p-value |
| --- | --- | --- | --- | --- | --- | --- | --- | --- |
| AVATAR:CBT | 2 | 0.49 | 0.0234 | -0.0502 | 0.095 | -0.1453 | -0.22 | 0.82 |
| AVATAR:TAU | 4 | 0.73 | -0.1733 | -0.1336 | -0.2788 | 0.1453 | 0.22 | 0.82 |
| CBT:TAU | 6 | 0.78 | -0.1967 | -0.2286 | -0.0833 | -0.1453 | -0.22 | 0.82 |
| Global inconsistency | Q |  |  |  |  |  |  | p-value |
| Between design | 0.04 |  |  |  |  |  |  | 0.84 |

Abbreviations: AVATAR = audio visual assisted therapy aid for refractory auditory hallucinations; CBT = cognitive behavioral therapy; NMA = network meta-analysis; TAU = treatment-as-usual

Appendix 11. Node-splitting and global inconsistency results for anxiety symptoms

| Node-splitting | k | Prop | NMA | Direct | Indir. | Diff | z | p-value |
| --- | --- | --- | --- | --- | --- | --- | --- | --- |
| AVATAR:CBT | 1 | 0.34 | -0.2763 | -0.4323 | -0.1975 | -0.2348 | -0.59 | 0.56 |
| AVATAR:Psychoeducation | 1 | 0.71 | -0.0926 | -0.0153 | -0.2803 | 0.265 | 0.54 | 0.59 |
| AVATAR:TAU | 2 | 0.76 | -0.4008 | -0.388 | -0.4402 | 0.0522 | 0.14 | 0.89 |
| CBT:Psychoeducation | 1 | 0.47 | 0.1837 | 0.0441 | 0.3091 | -0.265 | -0.54 | 0.59 |
| CBT:TAU | 4 | 0.73 | -0.1245 | -0.1387 | -0.0865 | -0.0522 | -0.14 | 0.89 |
| Global inconsistency | Q |  |  |  |  |  |  | p-value |
| Between design | 0.29 |  |  |  |  |  |  | 0.82 |

Abbreviations: AVATAR = audio visual assisted therapy aid for refractory auditory hallucinations; CBT = cognitive behavioral therapy; NMA = network meta-analysis; TAU = treatment-as-usual

Appendix 12. Node-splitting and global inconsistency results for quality of life

| Node-splitting | k | Prop | NMA | Direct | Indir. | Diff | z | p-value |
| --- | --- | --- | --- | --- | --- | --- | --- | --- |
| AVATAR:CBT | 2 | 0.84 | 0.188 | 0.1718 | 0.2758 | -0.1041 | -0.17 | 0.86 |
| AVATAR:TAU | 1 | 0.25 | 0.5129 | 0.5906 | 0.4865 | 0.1041 | 0.17 | 0.86 |
| CBT:TAU | 4 | 0.9 | 0.3249 | 0.3147 | 0.4188 | -0.1041 | -0.17 | 0.86 |
| Global inconsistency | Q |  |  |  |  |  |  | p-value |
| Between design | 0.01 |  |  |  |  |  |  | 0.91 |

Abbreviations: AVATAR = audio visual assisted therapy aid for refractory auditory hallucinations; CBT = cognitive behavioral therapy; NMA = network meta-analysis; TAU = treatment-as-usual

Appendix 13. Node-splitting and global inconsistency results for all-cause discontinuation

| Node-splitting | k | Prop | NMA | Direct | Indir. | Diff | z | p-value |
| --- | --- | --- | --- | --- | --- | --- | --- | --- |
| AVATAR:CBT | 2 | 0.24 | 1.28 | 1.413 | 1.2401 | 1.1394 | 0.23 | 0.82 |
| AVATAR:Psychoeducation | 1 | 0.77 | 0.986 | 0.8571 | 1.5834 | 0.5413 | -0.82 | 0.41 |
| AVATAR:TAU | 3 | 0.73 | 1.1948 | 1.252 | 1.0515 | 1.1907 | 0.35 | 0.73 |
| CBT:Psychoeducation | 1 | 0.35 | 0.7703 | 1.15 | 0.6225 | 1.8473 | 0.82 | 0.41 |
| CBT:SPT | 5 | 0.85 | 0.9531 | 0.9253 | 1.134 | 0.8159 | -0.37 | 0.71 |
| CBT:TAU | 11 | 0.85 | 0.9335 | 0.9082 | 1.0903 | 0.833 | -0.44 | 0.66 |
| SPT:TAU | 2 | 0.61 | 0.9794 | 0.9061 | 1.1041 | 0.8207 | -0.46 | 0.65 |
| Global inconsistency | Q |  |  |  |  |  |  | p-value |
| Between design | 0.95 |  |  |  |  |  |  | 0.92 |

Abbreviations: AVATAR = audio visual assisted therapy aid for refractory auditory hallucinations; CBT = cognitive behavioral therapy; NMA = network meta-analysis; SPT = supportive psychotherapy; TAU = treatment-as-usual
